# Differential gene retention as an evolutionary mechanism to generate biodiversity and adaptation in yeasts

**DOI:** 10.1038/srep11571

**Published:** 2015-06-25

**Authors:** Guillaume Morel, Lieven Sterck, Dominique Swennen, Marina Marcet-Houben, Djamila Onesime, Anthony Levasseur, Noémie Jacques, Sandrine Mallet, Arnaux Couloux, Karine Labadie, Joëlle Amselem, Jean-Marie Beckerich, Bernard Henrissat, Yves Van de Peer, Patrick Wincker, Jean-Luc Souciet, Toni Gabaldón, Colin R. Tinsley, Serge Casaregola

**Affiliations:** 1 INRA UMR1319, Micalis Institute, CIRM-Levures, 78850 F-Thiverval-Grignon, France; 2 AgroParisTech UMR1319, Micalis Institute, 78850 F-Thiverval-Grignon, France; 3 Department of Plant Systems Biology VIB, Technologiepark 927, 9052 Gent, Belgium; 4 Department of Plant Biotechnology and Bioinformatics, Ghent University, Technologiepark 927, 9052 Gent, Belgium; 5 Bioinformatics and Genomics Programme, Centre for Genomic Regulation, Dr. Aiguader 88, Barcelona 08003, Spain; 6 Universitat Pompeu Fabra (UPF), Barcelona 08003, Spain; 7 INRA UMR1163, Biotechnologie des Champignons Filamenteux, Aix-Marseille Université, Polytech Marseille, 163 avenue de Luminy, CP 925, 13288 Marseille Cedex 09, France; 8 CEA, Institut de Génomique, Genoscope, 2 Rue Gaston Crémieux, Évry F-91000, France; 9 INRA UR1164, Unité de Recherche Génomique – Info, 78000 Versailles, France; 10 CNRS, UMR 7257, Aix-Marseille Université, 13288 Marseille, France; 11 Genomics Research Institute, University of Pretoria, Hatfield Campus, Pretoria 0028, South Africa; 12 CNRS UMR 8030, 2 Rue Gaston Crémieux, Évry, 91000, France; 13 Université d’Evry, Bd François Mitterand, Evry,91025, France; 14 Université de Strasbourg, CNRS UMR7156, Strasbourg, 67000, France

## Abstract

The evolutionary history of the characters underlying the adaptation of microorganisms to food and biotechnological uses is poorly understood. We undertook comparative genomics to investigate evolutionary relationships of the dairy yeast *Geotrichum candidum* within Saccharomycotina. Surprisingly, a remarkable proportion of genes showed discordant phylogenies, clustering with the filamentous fungus subphylum (Pezizomycotina), rather than the yeast subphylum (Saccharomycotina), of the Ascomycota. These genes appear not to be the result of Horizontal Gene Transfer (HGT), but to have been specifically retained by *G. candidum* after the filamentous fungi–yeasts split concomitant with the yeasts’ genome contraction. We refer to these genes as SRAGs (Specifically Retained Ancestral Genes), having been lost by all or nearly all other yeasts, and thus contributing to the phenotypic specificity of lineages. SRAG functions include lipases consistent with a role in cheese making and novel endoglucanases associated with degradation of plant material. Similar gene retention was observed in three other distantly related yeasts representative of this ecologically diverse subphylum. The phenomenon thus appears to be widespread in the Saccharomycotina and argues that, alongside neo-functionalization following gene duplication and HGT, specific gene retention must be recognized as an important mechanism for generation of biodiversity and adaptation in yeasts.

Comparative genomics is a powerful tool for the investigation of yeast evolution^1^,^2^. Genome sequences are now available for a large number of Saccharomycetaceae and Debaryomycetaceae species within the subphylum Saccharomycotina^3^,^4^,^5^,^6^,^7^,^8^,^9^,^10^,^11^,^12^. Species associated with the *Pichia*/*Ogatea* clade such as *Dekkera bruxellensis*, *Komagataella pastoris*, *Ogataea polymorpha* and *Kuraicha capitulata* have also attracted a great deal of attention^13^,^14^,^15^,^16^, but the basal lineages of the Saccharomycotina remain poorly studied. To date the sequences of only two genomes of basal species, *Yarrowia lipolytica*^6^ and *Blastobotrys adeninivorans*^17^, have been reported.

The ubiquitous species, *Geotrichum candidum* (teleomorph = *Galactomyces candidus*), a member of the basal family the Dipodascaceae, can be found in a wide range of habitats from plant tissue and silage, to soil, air, water, milk and cheese^18^,^19^,^20^. *G. candidum* is well-known as an important component of the surface microbiota of soft cheeses and has also been used as a starter in the cheese industry^21^. It is also involved in beer making^22^ and industrial enzyme production^23^. In addition, *G. candidum* presents unusual characteristics that have complicated its taxonomic classification. For instance, it displays high morphological variability and wide phenotypic diversity, and has many features generally associated with filamentous fungi. Although initially classified as yeast by the two major yeast taxonomic monographs^24^,^25^, it was later reclassified as a mould or filamentous yeast-like fungi^18^,^26^.

Saccharomycotina yeasts have greatly contributed to the understanding of major molecular evolutionary mechanisms leading to functional diversity such as gene duplication followed by neo- or sub-functionalization^4^,^9^,^17^,^27^,^28^,^29^,^30^,^31^,^32^,^33^. Recent developments have shown that horizontal gene transfers (HGT) also contributes to the diversity between species^34^,^35^,^36^. However, these two gene-gain processes alone cannot account for most of the major and rapid transitions during yeast evolution such as the split between Pezizomycotina (filamentous fungi) and Saccharomycotina (yeasts) that was associated with genome contraction in the Saccharomycotina subphylum. Based on our whole genome comparisons between *G. candidum* and the other ascomycetes, we show that significant differential gene loss has occurred in lineages associated to major evolutionary transitions in yeasts, underscoring this evolutionary mechanism as an important force shaping genomic and functional diversity.

## Results

### Overall characteristics of the *G. candidum* CLIB 918 genome

A draft genomic sequence of high-quality of *Geotrichum candidum* strain CLIB 918 ( = ATCC 204307) was obtained by combining 454 pysosequencing of an 8 kb mate-pair library, Illumina/Solexa sequencing of genomic fragments, and a single whole genome shotgun 454 pyrosequencing run. The final assembly yielded 134 scaffolds with 1416 sized gaps, as highly repeated sequences such as transposable elements are typically missing from the assembly. We estimated the number of transposons and related elements to be of the order of 1000, corresponding to the gaps in the sequence assembly (Supplementary Note). A preliminary analysis based on scaffold size and presence of genes shortlisted the 27 largest scaffolds, totaling 24.2 Mb, *i.e.* 97.5% of the assembly. The 107 remaining scaffolds were merged into the artificial scaffold 32 with a size of 620.6 kb. The genome had a GC content of 48% and its size was estimated to be 24.8 Mb by the Newbler assembler. As such, it constitutes the largest Saccharomycotina yeast genome described to date, 25% larger than that of *Y. lipolytica* with 20.5 Mb^6^. The overall number of protein-coding genes in CLIB 918 is 6804 (excluding transposons and pseudogenes). The data are summarized in Table 1, Supplementary Table S1 , and Supplementary Note. In addition to the nuclear genome, the mitochondrial genome was also sequenced, assembled and annotated (Supplementary Fig. S1), producing a single, circular contig of length 29 kb and with 27.6% GC.

Automated annotation followed by manual curation identified 4713 genes presenting unambiguous sequence similarity to *Saccharomyces cerevisiae* and 1245 genes coding for conserved hypothetical proteins with similarity to fungal proteins but no clear ortholog in *S. cerevisiae*. The latter set of genes included 371 ORFs to which functions could be tentatively assigned based on comparison against annotated genomes and conserved domains, 34 genes encoding subunits of the NADH-ubiquinone oxidoreductase complex 1 (Supplementary Table S2), 27 genes with unique fungal homologs. Further, we found 846 genes with no similarity to any gene outside *G. candidum*. Finally, we identified three cases of bacterial HGT (Supplementary Data 1).

**Figure 1:**
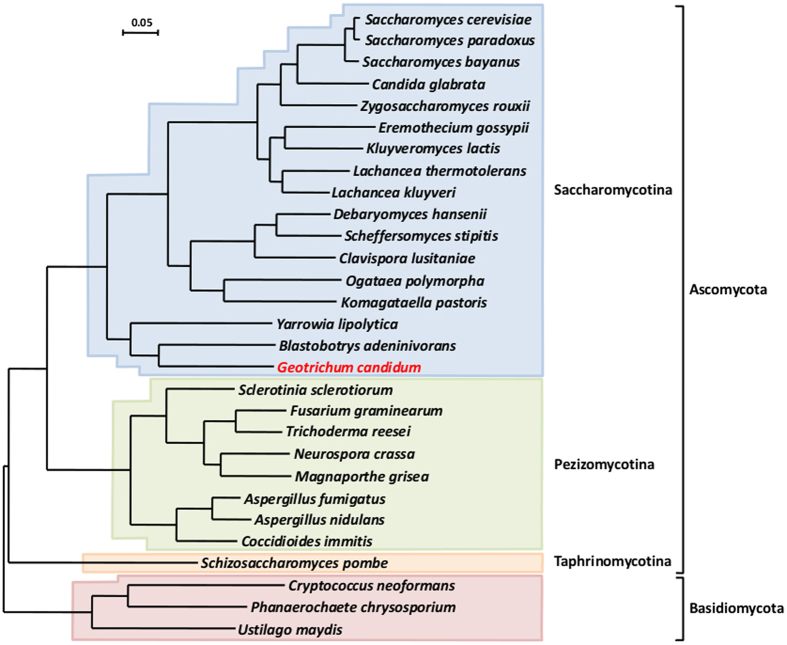
Phylogenetic position of *G. candidum*. Maximum likelihood phylogenomic reconstruction of 29 fungal species based on 246 concatenated gene sequences. The analysis was based on 64,105 informative positions remaining after curation of the 176,113 original aligned amino acids. Percentage bootstrap values for 100 replicates were 100% at each node. The bar represents 5 amino acid changes per 100 amino acids.

Phylogenomic analysis performed on the 246 genes previously identified by Aguileta and coworkers^37^, unambiguously placed *G. candidum* within the Saccharomycotina subphylum, with *B. adeninivorans* and *Y. lipolytica* as its closest neighbors. However, the branch lengths indicate that these species are not closely related (Fig. 1). This observation was confirmed by the reduced synteny existing between *G. candidum* and the two other basal species (Supplementary Fig. S2). As little as 778 and 511 syntenic blocks were identified between *G. candidum* and *B. adeninivorans* or *Y. lipolytica*, respectively (Supplementary Table S3). The large majority of these blocks comprised only 2 genes (50% of the blocks of synteny with *B. adeninivorans* and 64% of these with *Y. lipolytica*) or 3 genes (31% and 26%, respectively).

*G. candidum* genes are characterized by an average of 0.56 introns per protein-coding gene (3830 introns in 6804 ORFs). Thirty-five percent (2414) of the genes have at least one intron. This high intron content and the short intron size (71 nt median) depart from the situation in other yeasts. (Supplementary Fig. S3, Supplementary Table S4). Indeed, the number of introns in *G. candidum* is 12.9-fold higher than in *S. cerevisiae* and 3.4-fold higher than in *Y. lipolytica*, the most intron-rich Saccharomycotina yeast described to date (Table 1). Finally, a striking feature of the spliceosomal introns in *G. candidum* is the poor conservation of the 5’ splice site and the branch point when compared to other yeast within Saccharomycotina^38^ (Supplementary Fig. S4; Supplementary Note).

*G. candidum* has a sexual state^39^. A single gene (GECA02s02545g) coding for a protein of 281 amino acids that we have named *MATA* was identified on the basis of its sequence similarity with other fungal *MAT* genes and its position in a chromosomal region sharing a conserved organization with that of mating type loci in other yeasts and fungal species (Supplementary Fig. S5). In a survey of *G. candidum* strains we identified the *MATB* idiomorph, indicating that this species is heterothallic (Supplementary Note).

### Functional analysis and gene family expansion

To gain insight into the evolutionary dynamics of *G. candidum* genes and compare this to other yeasts, we reconstructed the phylome (*i.e.* complete set of individual gene phylogenies) for *G. candidum* as described in Materials and Methods. The resulting phylogenies, stored in phylomeDB^40^; (www.phylomedb.org), span the evolution of yeasts across the main Dikarya groups (Ascomycota and Basidiomycota). The phylome was analyzed to bring to light *G. candidum*-specific duplications, and infer orthology and paralogy relationships.

This analysis showed that *G. candidum* has 56 amplified gene families, that is, groups of paralogs containing three or more genes (Supplementary Data 2). The most highly amplified gene family (unknown function) with 21 copies has no counterpart in any other genome. The second largest expansion contains 16 members in a *GRE2*-like gene family, *GRE2* being a pleiotropic gene involved in ergosterol biosynthesis and control of filamentous growth in *S. cerevisiae*^41^,^42^. This gene family is also amplified in most other yeasts, but to a lesser extent. Finally, the category of transporters and permeases is also highly amplified in *G. candidum*, both general permeases and, more specifically, allantoate permeases and transporters for bile acid, nicotinic acid and monocarboxylate.

The number of genes involved in chitin metabolism is striking, as many of the genes of this pathway are present in more than one copy. Interestingly, six copies of the ortholog encoding chitin synthase III (*CHS3*-like), necessary for the majority of cell wall chitin synthesis, are found. This analysis also revealed six co-orthologs (including a pseudogene) of the activator of chitin synthase III (*SKT5*). Indeed, the closely-related *Y. lipolytica*, a dimorphic species with a strong tendency to form filaments, contains only three chitin synthase-related genes and a single *SKT5* regulator (Supplementary Table S5). The high number of genes involved in chitin metabolism compared with other yeasts correlates with the phenotype of high production of hyphae and pseudo-hyphae in *G. candidum*.

*G. candidum* is a major component of the microbiota of soft cheeses. In agreement with its propensity for growth in the dairy ecosystem, an expanded family with a total of four carboxylesterase/type B lipase genes was identified, of which two have previously been cloned and sequenced^23^,^43^ (Supplementary Table S6). Interestingly, none of these genes had an equivalent in the Saccharomycotina subphylum, but had homologs in the Pezizomycotina (see later section on specific gene retention). These lipases were predicted from their sequence to be secreted extracellular enzymes, in accordance with the first step of triacylglycerol catabolism in the dairy matrix involving secreted lipases. Volatile sulfur compounds, key to cheese aroma, are produced from the catabolism of methionine and cysteine by yeasts^44^. *S*even of the genes in this pathway are duplicated in *G. candidum* (Supplementary Fig. S6), in accordance with its known preeminent role in the cheese ripening process^45^, and a putative domestication of this yeast.

The most surprising gene amplification concerned gene families involved in the degradation of plant polysaccharides which are typically associated with filamentous fungi. *G. candidum* has undergone amplification of three distinct families of cellulolytic enzymes (Supplementary Data 2). These, included four copies of an endogluconase GH45, five copies of a lytic polysaccharide monooxygenase and five copies of an endo-polygalacturonase. Such functions have not been described in yeasts, except for a single gene encoding an endo-gluconase GH45 in *K. pastoris*^46^ and one distantly related polygalacturonase in *S. cerevisiae*^47^,^48^. These enzymes, whose presence greatly varies among fungi, are responsible for plant cell wall polysaccharide degradation, leading to cell-wall decomposition in a saprophytic or pathogenic context^49^. The gene complement of carbohydrate degrading enzymes is unique in *G. candidum* among yeasts (Supplementary Note. Supplementary Data 3). Further experimental investigations will be necessary to validate the hypothesis that this permits the use of a broad range of carbon and energy sources. The overall distribution of the annotated gene functions is shown in Supplementary Fig. S7a,b,c,d.

### Specifically retained ancestral genes in *G. candidum*

Functional annotation of the *G. candidum* genome was performed using the proteome of *S. cerevisiae* as well as those of other taxa of Saccharomycotina, Pezizomycotina and Basidiomycota. An initial analysis by BlastP, showed that there exist a set of few hundred *G. candidum* genes which do not have any orthologs in any sequenced Saccharomycotina species, but which display a good level of sequence conservation with predicted proteins from filamentous fungi (Pezizomycotina and Basidiomycota).

**Figure 2:**
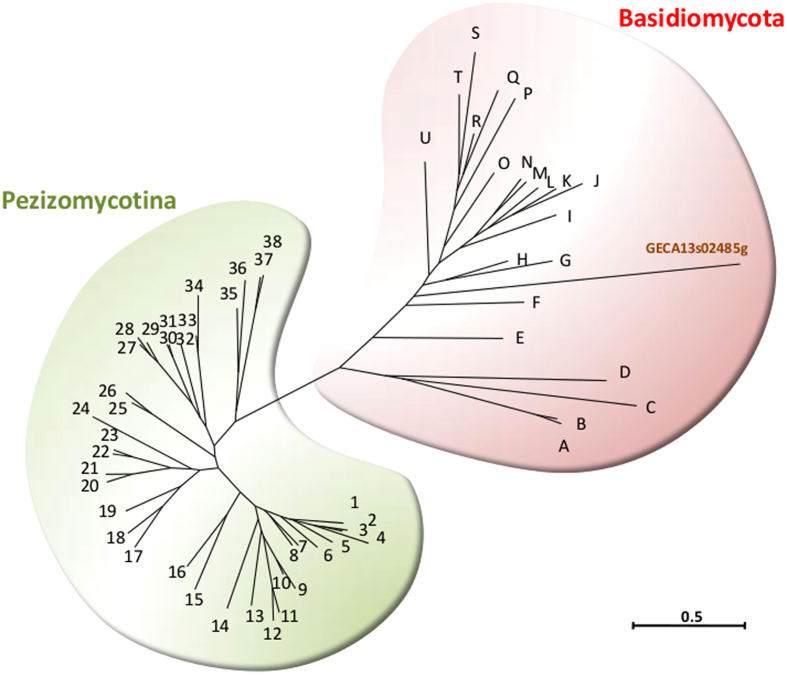
Phylogenetic position of the *G. candidum* gene GECA13s02485g potentially encoding a spermine synthase among Pezizomycota and Basidiomycota orthologs. Sequences of the fungal genes most closely related to GECA13s02485g were retrieved from NCBI after Blast comparison to Pezizomycotina and to Basidiomycota. Sequences were aligned using MUSCLE, the alignment was curated using Gblocks and the phylogenetic reconstruction was performed using Phyml with default settings as implemented in phylogeny.fr (http://www.phylogeny.fr/). The list of species can be found in Supplementary Data 6.

A detailed analysis of the topology of the phylogenies for each of the predicted proteins (phylome analysis) showed that 280 genes (4.1% of the 6804 *G. candidum* genes) presented discordant phylogenies. The simplest explanation, and that most often put forward, for the presence of such genes is that they are the result of horizontal gene transfer (HGT), which has been shown to occur, albeit infrequently, between eukaryotes^35^,^50^,^51^. In this respect, we identified a total of 17 clear cases of HGT from filamentous fungi, where the *G. candidum* gene grouped outside the Saccharomycotina, either within the sister subphylum Pezizomycotina (16 genes; Table 2 and Supplementary Fig. 8) or outside the Ascomycota (1 gene). In this latter case, the *G. candidum* gene (GECA13s02485g, putatively involved in polyamine metabolism) grouped within the Basidiomycota (Fig. 2). To the best of our knowledge, this is the first report of a gene horizontally transferred from the Basidiomycota to a Saccharomycotina species (Supplementary Note).

However, the remaining 263 of the 280 discordant genes did not appear to be due de HGT, grouping phylogenetically neither within the Saccharomycotina, nor within the Pezizomycotina. Further analysis revealed that 141 of these 263 genes had no orthologs within the Saccharomycotina, but counterparts in Ascomycota or in Ascomycota and in Basidiomycota (131 in Pezizomycotina subphylum, of which 45 were also present in the basidiomycetes). We call this group of genes set A (Supplementary Data 4). The other 122 genes were associated with a homolog in *S. cerevisiae*, presenting in contrast a phylogeny which followed that of the species tree. We denote this second group of genes as set B (Supplementary Data 4).

**Figure 3:**
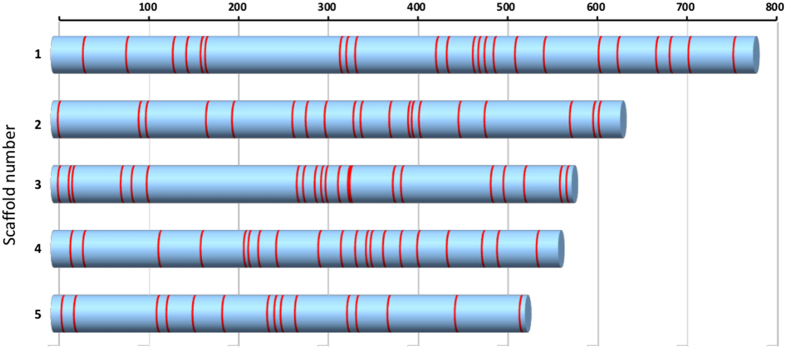
Distribution of the phylogenetically discordant sets A and B genes on the five largest scaffolds of the *G. candidum* genome. Scaffolds are represented as horizontal bars, numbered at the left, and red lines show the position of SRAGs. The scale indicates gene number.

In order to elucidate the origins and history of these genes of discordant phylogeny, we compared their characteristics with those that would be expected of horizontally-transferred genes. In most cases of HGT described in yeasts, the genes involved were exclusively clustered and had resulted from introgressions^13^,^52^,^53^. In filamentous fungi, HGT affects few single genes, but mostly larger regions of DNA, typically containing functionally related groups of genes^54^. In contrast, the set A and B *G. candidum* genes were found to be scattered through the genome sequence and did not cluster together as part of larger regions of transferred DNA (Fig. 3). In addition, these genes were distributed in the scaffolds independently of functional class.

**Figure 4:**
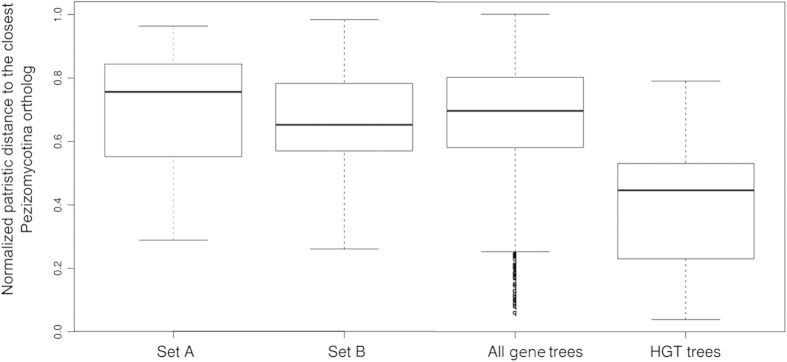
Phylogenetic distance of HGT and sets A and B genes from *G. candidum* to Pezizomycotina. Normalized distances between each *G. candidum* gene and its closest ortholog in the *Pezizomycotina* are represented as box plots. The graphs show the maximum, minimum and median values and the first and third quartiles. The points at the bottom of the “All gene trees” box plot are outliers, whose phylogenetic distance from the traced box is greater than 1.5 times the interquartile distance.

HGT can usually be detected because the phylogenetic position of the transferred genes with respect to homologs in related species differs from that of the other genes within the genome. Patristic distances (i.e. sum of branch lengths separating two tree nodes) between each *G. candidum* gene and their counterparts in the Pezizomycotina species were calculated from the phylome. Figure 4 presents the normalized patristic distances of the *G. candidum* genes, including the set A genes, the set B genes, all the *G. candidum* genes and the hypothetical HGT genes, from their closest Pezizomycotina orthologs. This analysis shows that the genes showing discordant phylogenies, both set A and set B, are not distinguishable from the entire gene complement of *G. candidum* in terms of their distances to Pezizomycotina orthologs. On the other hand, the normalized patristic distance between the HGT genes and their Pezizomycotina orthologs is clearly reduced. Genes originating from lateral transfers would be expected to display a reduced distance from their Pezizomycotina orthologs, since they are more or less recently diverged. The fact that distances between Pezizomycotina and set A and set B genes are not different from distances between Pezizomycotina and the *G. candidum* genes rules out the possibility that the set A and B genes were the result of HGT.

**Figure 5:**
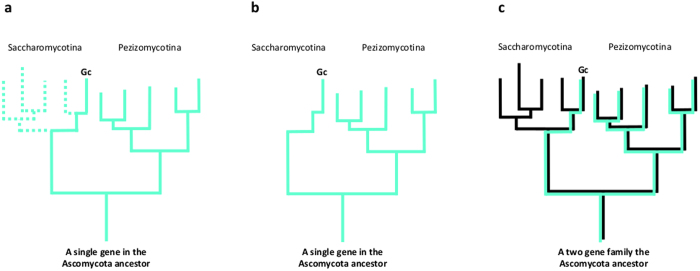
Schematic representation of the origin of SRAGs. (**a**) The hypothetical fate of a gene transmitted vertically to the Pezizomycotina and the Saccharomycotina lineages from the Ascomycota ancestor is represented by a continuous green line. The dotted line indicates the lineages in which the gene is lost, (**b**), resulting in a situation where the gene is found in the Pezizomycotina lineage and only in *G. candidum* where it has been retained (set A genes). (**c**) Transmission of members of a duplicated gene family in the Ascomycota ancestor to the Pezizomycotina and the Saccharomycotina lineages (set B genes). The green line indicates that one paralog has been lost in the entire Saccharomycotina lineage, except in *G. candidum* where it has been retained (similarly to (**a**) and (**b**)). The black line indicates that the second paralog has been transmitted to the Saccharomycotina lineage. Whereas only one paralog is present in the Saccharomycotina, both paralogs are present in *G. candidum*.

For all these reasons, it seems highly unlikely that the genes of sets A and B result from HGT events. Rather, a more plausible explanation considering the above observations would be that they had been specifically retained during the radiation after the separation of the Pezizomycotina and Saccharomycotina. We therefore propose to designate this type of gene as a Specifically Retained Ancestral Gene (SRAG). Figure 5 presents the proposed scheme leading to the occurrence of SRAGs in a present day yeast species such as *G. candidum* (Fig. 5).

**Figure 6:**
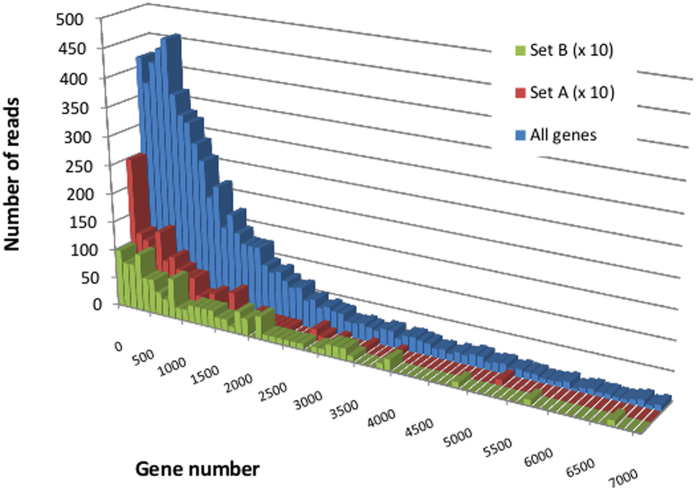
Expression of genes with discordant phylogenies. The distribution of the RNA sequence reads was plotted against the genes of setA, setB and against the whole genome. The number of genes in sets A and B are shown multiplied by a factor of 10 to facilitate comparison.

The expression of genes with a discordant phylogeny was compared to the rest of the genes using data from high throughput RNA sequencing. We observed that the overall expression level of the set A was reduced compared to the rest of the genes in the genome (Reduction of 1.4-fold, P < 10^−7^). The overall gene expression of set B genes was not significantly different to that of the other genes (P = 0.84) (Table 3; Fig. 6). This reduced expression may be due to a higher specificity of the genes in the set, including lignocellulolytic enzymes and a number of transcription factors, which might not be expressed under the chosen laboratory growth conditions.

### SRAGs are a common feature in yeasts

We examined other well-characterized yeast genomes to investigate whether such genes could also be found. To this end, we reconstructed the phylomes of three other species: *S. cerevisiae*, *Debaryomyces hansenii* and *Y. lipolytica.* A search in PhylomeDB for genes with discordant phylogeny permitted the identification of putative SRAGS in these species. Again we detected genes with orthologs in Pezizomycotina only as well as genes with discordant phylogeny which were present in the Pezizomycotina and absent from a majority of Saccharomycotina (Supplementary Data 5).

*S. cerevisiae* was found to have 15 genes presenting discordant phylogenies (Table 4, see www.phylomedb.org/phylome_236). These *S. cerevisiae* genes are involved in a variety of pathways (respiration, cell wall, post-transcriptional quality control, protein translation, sterol uptake); two of them are of unknown function. Interestingly, none of these 15 genes are essential for growth under normal conditions (*PDR11*, a sterol uptake protein, is however required for anaerobic growth, where sterol biosynthesis is compromised^55^; they are all expressed in either unusual or stressful conditions for *S. cerevisiae* (http://www.yeastgenome.org). The *IRC7* gene, encoding a putative cystathionine beta-lyase, was proposed to be the result of HGT, originating in bacteria^56^; however, this gene proved unambiguously closer to Pezizomycota than to bacterial counterparts (data not shown).

**Figure 7:**
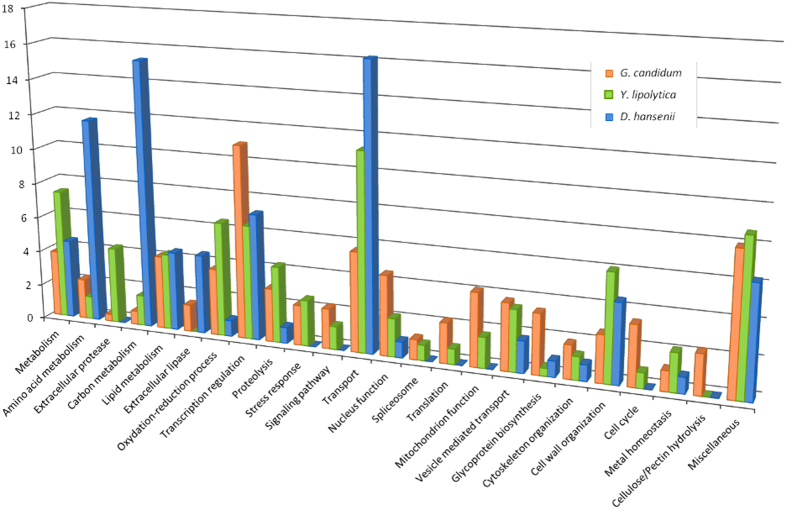
Functional distribution of SRAGs in three yeasts species. The SRAGs of *D. hansenii*, *Y. lipolytica* and *G. candidum*, as listed in Supplementary Data 6, were assigned to functional categories. For each species, the distribution of SRAGs by category is expressed as a percentage of the total number of SRAGs. Orange, *G. candidum*; blue, *D. hansenii*; green, *Y. lipolytica*.

Functional analysis of the genes in the *G. candidum, D. hansenii* and *Y. lipolytica* revealed that SRAGs are associated with diverse functional classes and that they are responsible for at least part of the specificity, but functional classes are shared between these yeasts. A functional classification of the SRAGS highlighted differences between *D. hansenii* and the two other basal yeasts *G. candidum* and *Y. lipolytica* (Fig. 7).

The halophilic and psychrophilic yeast *D. hansenii* is found in environments such as seawater, brine and salted foods and is a major component of cheese surface microbiota^57^. The functional classes overrepresented in the SRAG gene set are those of *Amino acid metabolism* (13 genes), *Carbon metabolism* (with seven SRAGs involved glycosidic bond hydrolysis) and *Transport* (with nine SRAGs involved in sugar transport). There are also five extracellular lipases that hydrolyze triacylglycerols in this lipid-rich environment to fatty acids and to glycerol, which is the main compatible osmolyte accumulated by *D. hansenii* as osmoprotectant on the highly saline cheese-surface^58^. Thus, *D. hansenii* SRAGs are representative of functions needed to grow under these conditions.

*Y. lipolytica* has long been a focus of research for its lipid metabolisms and its capacities for protein secretion^59^,^60^. It is encountered on the surface of ripened cheese^61^,^62^. The functions that are over-represented in *Y. lipolytica* SRAGs are *Lipid metabolism* (10 genes) and *Proteolysis* (20 genes, of which 10 encode extracellular proteases). *Y. lipolytica* and *G. candidum* are both dimorphic yeasts, whose transition from budding to hyphal growth involves complex subcellular processes. We built an inventory of the *Y. lipolytica* and *G. candidum* genes homologous to *N. crassa* genes necessary for filamentous growth^63^ (Supplementary Table S8). Among the 55 *Y. lipolytica* genes and 70 *G. candidum* genes in the inventory, respectively 29 and 37 SRAGs were found. Thus, over 50% of the *Y. lipolytica* and *G. candidum* genes necessary for filamentous growth are SRAGs, contrasting with the proportion of SRAGs in the whole genomes, (3.7% and 3.9% in *Y. lipolytica* and *G. candidum*, respectively), and highlights the strong association of SRAGs with filamentous growth.

In the case of *G. candidum*, with the exception of functions related to filamentous growth, the presence of SRAGs in the various functional categories is generally low, varying from 1 to 4%. The exception of the large number of *G. candidum* SRAGs in the *Transcription regulation* (11%) category is an indication that the reactivity and adaptability of this yeast to environmental changes may be carried by SRAGs. Our analysis of the functional classification of these SRAGs highlighted the specific properties of these yeasts according to their natural morphology and ecological niche. SRAGs contribute to phenotypic specificity of these yeasts. An over-representation of the *Transcription regulation* and *Transport* categories is expected in wild yeasts as they have to adapt to various environments by being able to use a wide variety of nutrients and to reorganize gene expression in response to environmental changes. We also noted that each of the three yeasts examined, *D. hansenii*, *Y. lipolytica* and *G. candidum*, possess SRAGs associated with lipid metabolism, which may be linked to their presence in dairy products. It is important to note that the genes in the “Lipid metabolism” category in all three species are phylogenetically unrelated, suggesting a parallel evolution. Indeed the same is true for most of the SRAGs, suggesting that these genes are interesting candidates for the analysis of species-specific technological properties.

## Discussion

The genome sequence of *G. candidum* permits new insights into the genome structure of yeasts and their evolution. In particular, its relative basal position among Saccharomycotina and its unusually large genome for a yeast, makes it ideal to investigate the ancestral genomic repertoire of this subphylum. Comparative genomics between *G. candidum* and other Saccharomycotina yeasts demonstrated the existence of groups of genes specific to *G. candidum* and greatly-amplified gene families which appear to contribute to the known phenotypic specificity of this yeast, while the significance of others, such as the large repertoire of carbohydrate hydrolases otherwise only found in filamentous fungi, can only be hypothesized. We were interested to study whether the origins of these genes specifically present in *G. candidum* could be explained by HGT or another mechanism, and therefore undertook further analyses based on individual gene phylogenies. This brought to light a larger group of genes with discordant phylogenies, of which some had no homologs within the Saccharomycotina. When such analysis was extended to other species representative of different lineages of the yeast phylogenetic tree it was seen that the presence of such genes is common to all the yeasts examined. We propose that such genes have been specifically retained after the split between Pezizomycotina and Saccharomycotina and during the subsequent genome reduction of the latter clade; we would therefore denote them Specifically Retained Ancestral Genes (SRAG). Several lines of evidence argue for this explanation, and against the simplest hypothesis, acquisition through HGT, for the presence of these genes in *G. candidum*: (i) The large evolutionary distance, similar to that of clear vertically-inherited genes, of the putative participants makes HGT unlikely. HGT between eukaryotes usually result from interspecific or intergeneric hybridization^64^,^65^,^66^, but, to the best of our knowledge (and excepting the case of HGT that we describe here with GECA13s024858g), inter-subphylum transfers between filamentous fungi and yeasts have not been documented. (ii) The phylogenetic distances separating the SRAGs from their orthologs were similar to those separating the other genes from their respective orthologs, whereas a hallmark of HGT is the phylogenetic closeness of the orthologs thus transferred. This is illustrated by the position of SRAGs being outside the Pezizomycotina clade in the phylogenetic trees. (iii) The number, and relative frequencies of SRAGs, present in the different species argues for specific retention rather than HGT. Indeed numerous SRAGs were found in each of the four yeasts examined (almost 4% of gene content in the case of *G. candidum*). It is unlikely that HGT events would occur at such a frequency. Furthermore the distribution of the numbers of SRAGs in the different yeasts is intriguing: of the species studied here, *G. candidum*, *Y. lipolytica* and *D. hansenii* possess a higher number of SRAGs than does *S. cerevisiae* (263, 230 and 111, respectively, compared to 15). Whereas we might expect a fairly constant frequency of genes with discordant phylogenies if their presence were due to HGT, there is a clear difference in their number, which may be due to their different evolutionary histories. This variability is also seen by the recent detection, in *B. adeninivorans*^17^, of 121 genes with orthologs only in Pezizomycotina, and in *Zygosaccharomyces bailii*^67^ , of 27 genes with similarity to filamentous fungal genes or highly divergent from yeasts, though the latter group attributed these to HGT.

Lineage-specific gene retention described following mitochondrial endosymbiosis in crown group eukaryotes^68^, and the co-occurrence of genes could be used to predict their functional links. Lineage-specific losses of genes associated with gain or loss of function have been reported in widely separated lineages^6^,^69^,^70^,^71^,^72^. In addition, a number of metabolic pathways present in the Pezizomycotina are not found in Saccharomycotina^73^,^74^,^75^. The latter authors observed a differential presence or absence of peroxysomal and non-peroxysomal pathways of β-oxidation in some yeasts and fungi, and proposed that the pathway has been duplicated in the ancestor and differentially lost or retained in the studied species. We expand this observation by a global comparison of four yeast genomes within the same subphylum. We define two categories of *G. candidum*-specific genes, based on their distributions:

1) One group of genes have orthologs within the Saccharomycotina, but are derived from the paralog in the common ancestor of Saccharomycotina and Pezizomycotina lost by the other yeasts. Lineage-specific gene retention following Whole Genome Duplication is well-known in organisms including *Saccharomyces* species^32^, filamentous fungi^76^, alveolates^77^, seed plants^78^ and vertebrates^79^. However, no such WGD has been described in the ascomycete ancestor, so the above-mentioned paralogs have probably resulted from gene duplications in the ancestor. This situation corresponds to that of the beta-oxidation genes described^75^; *G. candidum* has retained one of the paralogs, while the other Saccharomycotina species kept the other (Fig. 5). In some cases *G. candidum* had retained both genes of the ancestral duplication, for instance some snRNPs.

2) In *G. candidum*, in addition to the cases of gene retention after ancestral gene duplications, we discovered a second set of 141 genes in single copy in the Ascomycota ancestor, which was lost in the other Saccharomycotina species. Cases of specifically retained genes not derived from genomic duplication are rarely documented, although some have been proposed to play an important role in species differentiation^80^,^81^,^82^. Our analysis suggests however that this may be an important mechanism of generation of biodiversity, at least in the yeast subphylum studied.

The above discussion is limited to genes that were unique in each studied yeast species, but we also noted the existence of SRAGs present in two or more species. Further work on this class of SRAGs to determine their distribution within the subphylum, will certainly greatly increase our understanding of the evolution and biodiversity of the yeasts.

Thus, evolution by differential gene retention is widespread in a broad but well-defined clade, the Saccharomycotina. The distribution of SRAGs in distantly-related yeast species argues for a mechanism of a sustained loss throughout the yeast tree permitting adaptation of yeast species to various ecological niches and resulting in the genome reduction characteristic of yeasts, rather than a massive genome contraction in one branch of the Ascomycota.

Saccharomycotina yeasts use a combination of various mechanisms such as WGD^4^,^6^,^9^,^17^,^83^, gene duplication^6^,^83^ and HGT^6^,^36^,^56^,^84^,^85^,^86^,^87^, which contribute to generating biodiversity to a variable extent. To date, the major genetic mechanisms proposed to affect adaptation of fungi are duplication or gene amplification followed by neospecialization^28^,^32^,^33^ and HGT, the bacterial nitrate assimilation cluster is suggested to have contributed to the success of the Dikarya on land^88^ and the acquisition of genes to increase efficiency of alcoholic fermentation by *S. cerevisiae*^53^,^89^. Here we highlight the importance of another mechanism; yeasts that we have analyzed and probably others^17^,^67^ contain different proportions of SRAGs, which are associated with biochemical or growth characteristics of the species concerned, thus contributing to the great biodiversity shown by this group of organisms.

## Material and methods

### Strains

The sequenced *G. candidum* strain was isolated by Micheline Gueguen (University of Caen) from Pont-L’Evêque cheese in Normandy (France) in 1975. It has been shown to produce compounds that inhibit the growth of *Listeria* and has been extensively studied^90^,^91^,^92^,^93^,^94^,^95^. The strains used in this study, CLIB 918 (=ATCC 204307), CLIB 1368^NT^ (=CBS 615.84^NT^) and 61 *G. candidum* isolates are preserved at the CIRM-Levures (http://www6.inra.fr/cirm/Levures). They were routinely propagated on complete medium (YPD: yeast extract 10 g/L, peptone 20 g/L, glucose 20 g/L) at 28 °C.

### Preparation of DNA and RNA

DNA was extracted as previously described (Jacques *et al.*, 2009) from strain CLIB 918 grown in YNB_N5000_ (1.7 g/L Yeast Nitrogen Base, 20 g/L glucose, 5 g/L ammonium sulfate) at 28 °C to increase the yeast-like form and promote cell lysis. For RNA preparation, strain CLIB 918 was grown at 28 °C with agitation on three different media, i.e. complete medium (YPD), minimal medium (YNB_N5000_) and Synthetic Cheese Medium, SCM, described in^96^) to maximize the diversity of gene expression. Total RNAs were extracted using the method described by Mansour *et al.*^97^ from cultures grown in the three different conditions, and then pooled.

### 454 libraries preparation and sequencing

The single 454 library was constructed on genomic DNA (500 ng) according to the Roche standard procedure using RL adaptators (GS FLX Titanium Rapid Library Preparation Kit, Roche Diagnostic, USA). The 8 kb mate pair library was constructed following Roche 454 protocol. Briefly, 15 μg of genomic DNA was sheared to about 8 kb using HydroShear Instrument. Fragments were end-repaired and extremities were ligated with 454 circularization adaptors. After gel size selection of 8 kb bands and fill in, DNA fragments were circularized by Cre recombinase and remaining linear DNA digested by Plasmid Safe ATP dependent DNAse (Epicentre) and exonuclease I. Circular DNA was refragmented by nebulization. Fragments were end-repaired and ligated with library adaptors used for downstream processes. Mate pair library was amplified and purified. Both single and mate pair libraries were isolated, then bound to capture beads and amplified in an oil emulsion (emPCR). They were then sequenced using 1/2 Pico Titer Plate on 454 GSFlx instrument with Titanium chemistry (Roche Diagnostic, USA) according to the manufacturer protocol.

### Illumina GA library preparation and sequencing

The genomic DNA and cDNA were sonicated separately to a 150- to 1000-bp size-range using the Covaris E210 (Covaris Inc., MA). Fragments were end-repaired then 3‘-adenylated, and Illumina adapters were added using NEBNext Sample Reagent Set (New England Biolabs). Ligation products were purified and DNA fragments (>200 bp) were PCR-amplified using Illumina adapter-specific primers. After library profile analysis on an Agilent 2100 Bioanalyzer (Agilent Technologies, USA) for genomic DNA and Qubit quantification for cDNA, the respective libraries were sequenced using 76 base-length read chemistry in a single or paired-end flow cell on the Illumina GAIIx (Illumina, USA).

### Genome assembly and automatic error corrections with Solexa/Illumina reads

All 454 reads were assembled with Newbler version 2.3. From the initial 3,322,644 reads, 92.2% were assembled, yielding 1688 contigs that were linked into 134 scaffolds. The contig N50 (the contig size cut-off above which 50% of the total length of the draft sequence assembly is included) was 26.7 kb, and the scaffold N50 was 1.159 Mb. Cumulative scaffold size was 24.865 Mb. Sequence quality of scaffolds from the Newbler assembly was improved as described in Aury *et al.*^98^ by automatic error correction with Solexa/Illumina reads which have a different bias in error type compared to 454 reads. Following the correction process, we fixed 3415 mismatches and 6559 indels.

### Genome annotation

Gene models were predicted using Eugene pipeline^99^ on the URGI platform (http://urgi.versailles.inra.fr/). Eugene relies on combination of *ab initio* gene predictions (Eugene_IMM, SpliceMachine^100^ and Fgenesh http://www.softberry.com/berry.phtml) and similarity (BlastX against Swissprot and Trembl) evidences. All the gene models were then manually curated with the help of RNAseq data previously assembled with SOAP on the ORCAE platform (http://bioinformatics.psb.ugent.be/orcae/101) and visualized on GenomeView (http://genomeview.org^102^) and Artemis (http://www.sanger.ac.uk/resources/software/artemis/). All regions potentially coding for peptides of over 100 amino acids (aa) were annotated. CDS of less than 100 aa were only annotated when they presented sequence similarity with known proteins and/or associated with spliceosomal introns and were represented in the RNAseq library. The genes encoding tRNA were predicted using tRNAscan-SE (http://lowelab.ucsc.edu/tRNAscan-SE/) using default parameters. The protein coding genes were first functionally annotated by comparison with the *S. cerevisiae* genome. Genes that failed to show sufficient sequence similarity with *S. cerevisiae* genes were annotated by comparison against other available yeast genomes, filamentous fungal genomes and Swissprot; they received the annotation “conserved hypothetical protein” when their sequence showed similarity with that of proteins from several species. When a functional annotation was available in the databanks, it was associated to the “conserved hypothetical protein” annotation. Nomenclature for naming genes is the following: species name GECA, scaffold number from 1 to 27 and 32, s for scaffold, gene number with an incrementing step of 11, g for protein coding gene (for example, GECA01s00065g encodes a protein similar to *Saccharomyces* cerevisiae YNR018W), r for RNA coding gene (for example, GECA01s00238r encodes tRNA-Asp).

### Assembly and annotation of the mitochondrial genome

A total of four mtDNA contigs were identified. Ordering of contigs and junction was performed using PCR. Protein coding genes and ribosomal genes were detected using blastX against the available Saccharomycotina mtDNAs. tRNA genes were detected using tRNAscan-SE with default parameters and the mitochondrial search model (http://lowelab.ucsc.edu/tRNAscan-SE/).

### Phylogenomic analysis

Orthologs were first selected using blast with a P-value of 10^−5^ against proteomes of strains listed in Supplementary Table S9. Single-copy *G. candidum* genes were verified using ORCAE and homology was verified using Fungipath^103^. Sequences were concatenated and were aligned using MUSCLE v3.8^104^ with default settings. Alignments were curated using GBlocks v0.91b^105^. Species trees were reconstructed using PhyML v2.4.4^106^ with the WAG model. Bootstrap analysis was used to obtain branch support. Trees were visualized with *njplot*^107^.

### Synteny analysis

Conserved synteny blocks were defined using Synchro with default settings^108^. First, reciprocal blast hits were computed with a similarity threshold of 40% and length ratio between the two protein sequences smaller than 1.3. Second, syntenic homologs, which were not involved reciprocal blast hits, were added to the synteny blocks when they shared at least 30% of similarity over at least 50% of their length.

### Phylome reconstruction

A phylome comprises the collection of phylogenetic trees for each gene encoded in a genome. We reconstructed the *G. candidum* phylome in the context of 21 additional fungal species ranging across the main dikarya groups, i.e. 10 Saccharomycotina, 8 Pezizomycotina, one Taphrinomycotina and two Basidiomycota (Supplementary Table S9). An automatic pipeline described previously was used to reconstruct the phylome^109^. This pipeline includes the standard tree reconstruction steps: homology search, multiple sequence alignment and finally reconstructing the maximum likelihood tree. The homology search was performed using a Smith-Waterman search for each gene (seed gene) in the *G. candidum* genome (seed genome) against the protein database that contained the proteomes of interest. Results were filtered to select only sequences with an e-value below 10^−5^ and a continuous overlap of 0.5. A maximum of 150 sequences for each protein were considered. Homologous sequences were then aligned using three different alignment algorithms: MUSCLE v3.8^104^, MAFFT v6.712b^110^, and kalign^111^. Alignments were performed in forward and reverse direction using the head-or-tail approach^112^ and the 6 resulting alignments were combined with M-COFFEE^113^. TrimAl v1.3^114^ was used to clean the alignment (consistency-score cut-off 0.1667, gap-score cut-off 0.9). To reconstruct maximum likelihood trees, an evolutionary model needed to be selected. This was done by reconstructing a neighbor joining tree for each alignment using BioNJ^115^. The likelihood of the resulting topology according to one of 7 different models (JTT, LG, WAG, Blosum62, MtREV, VT and Dayhoff) was computed. The model best fitting the data, as determined by the AIC criterion^116^, was used to derive ML trees using phyML v 3.0 with four rate categories and inferring invariant positions from the data^117^. Branch support was computed using an aLRT (approximate likelihood ratio test) based on a chi^2^ distribution. Three additional phylomes were reconstructed using the same proteome set but with different species as seeds: *Saccharomyces cerevisiae*, *Y. lipolytica* and *Debaryomyces hansenii*. The resulting trees and alignments are stored in phylomeDB (http://phylomedb.org) with phylome IDs 233 (*G. candidum* phylome), 234 (*Y. lipolytica* phylome), 235 (*D. hansenii* phylome) and 236 (*S. cerevisiae* phylome).

### Species tree reconstruction

Proteins with a one-to-one orthology relationship to all the considered species were selected from the *G. candidum* phylome. The 302 protein alignments were concatenated into a multiple sequence alignment. The alignment was trimmed using trimAl v1.3^114^ to discard columns with more than 50% gaps (-gt 0.5 -cons 50). RAxML v8.0 was used to reconstruct the species tree^118^ using the PROTGAMMLG model (Supplementary Fig. S9). Additionally, a super-tree based species tree was derived from the *G. candidum* phylome using DupTree^119^.

### Phylome analysis

Trees in the phylome were scanned using ETE v2^109^ Trees were scanned to detect duplications that had occurred specifically in *G. candidum* by searching for clades that contained exclusively *G. candidum* sequences. Orthology and paralogy relations were inferred from the phylome trees using a species overlap algorithm^120^. Briefly, for each node in the tree, the algorithm tries to detect overlapping species at either side of the node. If there are overlapping species, the node is considered a duplication node and therefore the sequences are paralogs. If there are no overlapping species, then the node is considered a speciation node and sequences are orthologs. Finally, we used the phylome to assess phyletic distribution of genes, based on homology or orthology, and selected genes that had only homologs in each of the following six clades: i) the family Saccharomycetaceae (*S. cerevisiae*, *Zygosaccharomyces rouxii*, *Candida glabrata*, *Kluyveromyces lactis* and *Lachancea thermotolerans*), ii) the Saccharomycetales incertae sedis clade (*K. pastoris* and *O. angusta*), iii) the CTG clade (*D. hansenii* and *Clavispora lusitaniae*), iv) other fungi (*Ajellomyces capsulata*, *Aspergillus oryzae*, *Penicillium chrysogenum*, *Neurospora crassa*, *Cryptococcus neoformans*, *Ustilago maydis*, *Schizosaccharomyces pombe*, *Botrytis fuckeliana*, *Trichoderma reesei*, *Magnaporthe grisea*, and *Mycosphaerella graminicola*), v) *Y. lipolytica*, or vi) *G. candidum*. The same analysis was performed using the orthology predictions obtained from the phylomes (see above).

In order to calculate the patristic distances, trees that contained at least one ortholog in Pezizomycotina and at least one in any of the outgroup species (*S. pombe*, *U. maydis* and *C. neoformans*) were selected. For each of those trees the patristic distance was calculated between the *G. candidum* protein and its closest Pezizomycotina ortholog. This distance was then normalized by dividing it by the patristic distance between the same *G. candidum* sequence and its farthest orthologous outgroup.

### Gene expression analysis

Available RNAseq reads were mapped against the produced reference genome using the GSNAP software^121^ with default parameters. The resulting alignment files were transformed into raw read counts for each gene making use of htseq-count^122^ and the predicted G. candidum gene-models. To obtain the final expression values the raw read counts were normalized for CDS length. Afterwards subset of genes (and expression values) were created based on whether the gene has an ortholog in other Saccharomycotina (141 genes) or not (122 genes). The expression of the genes in these two subsets was then compared to the expression of all other genes in the genome. To investigate the potential difference in expression between the gene sets a Wilcoxon rank-sum test was applied.

## Additional Information

**Accession codes:** *Geotrichum candidum* genome sequence data have been deposited at EMBL under the accession number PRJEB4557, the mitochondrial genome of strain CLIB 918 and the *MATB* gene of strain CBS 615.84 were deposited under accession numbers HG530139 and HF558449, respectively.

**How to cite this article**: Morel, G. *et al.* Differential gene retention as an evolutionary mechanism to generate biodiversity and adaptation in yeasts. *Sci. Rep.* **5**, 11571; doi: 10.1038/srep11571 (2015).

## Supplementary Material

Supplementary Information

Supplementary Information

Supplementary Information

Supplementary Information

Supplementary Information

Supplementary Information

Supplementary Information

## Figures and Tables

**Table 1:** Genome characteristics comparison

**Species**	**Strain**	**No. of chromosomes**	**Genome size (Mb)**	**Average G + C content (%)**	**Total no. of CDS**	**Genome coding coverage (%)**	**Gene density (no. of CDS per 10 kb)**	**Average CDS size (codons)**	**Genes with introns**	**Total intron number**	**Total tRNA genes**
*S. cerevisiae*	S288c	16	12.1	38.3	5769	70.0	4.8	488	287	296	274
*Z. rouxii*	CBS 732	7	9.8	39.1	4992	76.1	5.1	497	158	162	272
*K. lactis*	CLIB 210	6	10.7	38.8	5076	69.2	4.8	485	129	129	163
*D. hansenii*	CBS 767	7	12.2	36.3	6395	74.2	5.2	479	420	467	200
*H. polymorpha*	DL1	8	9.0	47.8	5325	84,4	5.8	469	452	457	80
*Y. lipolytica*	CLIB 122	6	20.5	49.0	6580	46.0	3.1	489	984	1,119	510
***G. candidum***	**CLIB 918**	8*	**24.8**	**48.0**	**6804**	**44,9**	**2.7**	**516**	2415	**3,830**	**352**

Data from *S. cerevisiae* were taken from SGD (http://www.yeastgenome.org/)*; Z. rouxii*, *K.lactis*, *D. hansenii* and *Y. lipolytica* from Genolevures (http://www.genolevures.org and *H. polymorpha* from Ravin *et al.* (2013). Annotations for *G. candidum* are part of this work and are available from the ORCAE online database (http://bioinformatics.psb.ugent.be/orcae/). nd, not determined. *obtained from cytological analysis (Gente *et al.* 2002)

**Table 2:** List of putative HGTs from Pezizomycotina species to *G. candidum.*

**Phylome Id**	**Gene Id**	**Putative function**	**Closest relative species (blastp)**
Phy0064BLX_GEOCN	GECA03s05631g	unknown	*Arthroderma otae*
Phy0064BQT_GEOCN	GECA05s03486g	cyclin	Yarrowia lipolytica**
Phy0064BVF_GEOCN	GECA01s04828g	hydrolase, carbon-nitrogen family member	*Pyrenophora teres f. teres*
Phy0064BWG_GEOCN	GECA04s00318g	MFS sugar transporter	*Penicillium marneffei*
Phy0064C2U_GEOCN	GECA20s00758g	adipose-regulatory protein	*Penicillium chrysogenum*
Phy0064C4V_GEOCN	GECA13s00252g	Plasma membrane metalloid/H+ antiporter	*Aspergillus fumigatus*
Phy0064C92_GEOCN	GECA01s00538g	succinyl-CoA:3-ketoacid-coenzyme A transferase	*Talaromyces stipitatus*
Phy0064CD0_GEOCN	GECA04s06742g	golgi matrix protein	Yarrowia lipolytica**
Phy0064CEU_GEOCN	GECA12s03497g	unknown	*Aspergillus clavatus*
Phy0064CGG_GEOCN	GECA03s01220g	unknown	*Serpula lacrymans*
Phy0064D19_GEOCN	GECA24s00301g*	translation elongation factor	Komagataella pastoris**
Phy0064EQK_GEOCN	GECA07s00274g	COPI-coated vesicle protein	*Chaetomium thermophilum*
Phy0064FET_GEOCN	GECA05s00197g	MFS multidrug transporter	*Penicillium marneffei*
Phy0064FFJ_GEOCN	GECA08s04762g	Beta-isopropylmalate dehydrogenase (*LEU2*)***	Ogataea parapolymorpha**
Phy0064FX3_GEOCN	GECA20s00043g	Glutamine amidotransferase	*Fusarium oxysporum*
Phy0064G6V_GEOCN	GECA20s00065g	pyridoxine biosynthesis protein	*Gibberella zeae*

^*^partial gene sequence

^**^Saccharomycotina

^***^in addition to a Saccharomycotina ortholog (GECA07s02881g)

**Table 3:** Gene expression of SRAGs in *G. candidum.*

**Dataset**	**Rank sum**			**P-value**
	**Gene number**	**observed**	**expected**	
A + B	263	776 286	898 671	9.4.10^−5^
C	6 571	22 578 908	22 453 107	
				
A	141	346 497	473 196	<10^−7^
C	6 571	22 182 330	22 052 276	
				
B	122	412 587	408 273	8.4. 10^−1^
C	6 571	21 988 884	21 989 851	

**Table 4:** List of SRAGs in *S. cerevisiae.*

**Uniprot number**	**Gene name**	**Systematic name**	**Protein name**	**Function**
P40507	*AIR1*	YIL079C	Arginine methyltransferase-Interacting RING finger protein 1	Zinc knuckle protein; involved in nuclear RNA processing and degradation as a component of the TRAMP complex
Q07500	*NDE2*	YDL085W	External NADH-ubiquinone oxidoreductase 2, mitochondrial	External NADH dehydrogenase required for optimum cellular growth with a number of nonfermentable carbon sources, including ethanol
Q03081	*MET31*	YPL038W	Transcriptional regulator	Auxiliary transcriptional regulator of sulfur amino acid metabolism
Q06817	*GRS2*	YPR081C	Glycine--tRNA ligase 2	Catalyzes the attachment of glycine to tRNA(Gly)
P53059	*MNT2*	YGL257C	Alpha-1,3-mannosyltransferase	Mannosyltransferase involved in adding the 4th and 5th mannose residues of O-linked glycans
P38360	*PCA1*	YBR295W	P-type cation-transporting ATPase	Cadmium transporting P-type ATPase which plays a critical role in cadmium resistance by extruding intracellular cadmium
P43623	*IRC7*	YFR055W	Putative cystathionine beta-lyase	Beta-lyase involved in the production of thiols
P38143	*GPX2*	YBR244W	Glutathione peroxidase 2	May constitute a glutathione peroxidase-like protective system against oxidative stresses
Q12177	*na*	YLL056C	Uncharacterized protein	
P38150	*na*	YBR284W	Inactive deaminase	
Q03125	*NRG1*	YDR043C	Transcriptional regulator	Transcriptional repressor involved in regulation of glucose repression
Q08182	*YAP7*	YOL028C	AP-1-like transcription factor	Probable transcription activator linked to cell cycle that induces transcription activation of genes in the environmental stress response and metabolism control pathways, like the closely related YAP5
P53745	*MNT4*	YNR059W	Probable alpha-1,3-mannosyltransferase	
P39941	*DSF1*	YEL070W	Uncharacterized protein	Putative mannitol dehydrogenase
Q12390	*GTT2*	YLL060C	Glutathione S-transferase 2	Glutathione S-transferase capable of homodimerization
P40550	*PDR11*	YIL013C	ATP-dependent permease	Transporter involved in the uptake of sterol
P40186	*PCL7*	YIL050W	PHO85 cyclin-7	Cyclin partner of the cyclin-dependent kinase (CDK) PHO85

## References

[b1] Dujon B. Yeast evolutionary genomics. Nat Rev Genet 11, 512–24 (2010). 20559329 10.1038/nrg2811

[b2] Souciet J. *et al.* Genomic exploration of the hemiascomycetous yeasts: 1. A set of yeast species for molecular evolution studies. FEBS Lett 487, 3–12 (2000). 11152876 10.1016/s0014-5793(00)02272-9

[b3] Butler G. *et al.* Evolution of pathogenicity and sexual reproduction in eight *Candida* genomes. Nature 459, 657–62 (2009). 19465905 10.1038/nature08064 PMC2834264

[b4] Dietrich F. S. *et al.* The *Ashbya gossypii* genome as a tool for mapping the ancient Saccharomyces cerevisiae genome. Science 304, 304–7 (2004). 15001715 10.1126/science.1095781

[b5] Dietrich F. S., Voegeli S., Kuo S. & Philippsen P. Genomes of *Ashbya* fungi isolated from insects reveal four mating-type loci, numerous translocations, lack of transposons, and distinct gene duplications. G3 (Bethesda) 3, 1225–39 (2013). 23749448 10.1534/g3.112.002881 PMC3737163

[b6] Dujon B. *et al.* Genome evolution in yeasts. Nature 430, 35–44 (2004). 15229592 10.1038/nature02579

[b7] Gordon J. L. *et al.* Evolutionary erosion of yeast sex chromosomes by mating-type switching accidents. Proc Natl Acad Sci USA 108, 20024–9 (2011). 22123960 10.1073/pnas.1112808108 PMC3250169

[b8] Jeffries T. W. *et al.* Genome sequence of the lignocellulose-bioconverting and xylose-fermenting yeast *Pichia stipitis*. Nat Biotechnol 25, 319–26 (2007). 17334359 10.1038/nbt1290

[b9] Kellis M., Birren B. W. & Lander E. S. Proof and evolutionary analysis of ancient genome duplication in the yeast *Saccharomyces cerevisiae*. Nature 428, 617–24 (2004). 15004568 10.1038/nature02424

[b10] Kellis M., Patterson N., Endrizzi M., Birren B. & Lander E. S. Sequencing and comparison of yeast species to identify genes and regulatory elements. Nature 423, 241–54 (2003). 12748633 10.1038/nature01644

[b11] Scannell D. R. *et al.* The awesome power of yeast evolutionary genetics: new genome sequences and strain resources for the *Saccharomyces sensu stricto* genus. G3 (Bethesda) 1, 11–25 (2011). 22384314 10.1534/g3.111.000273 PMC3276118

[b12] Wendland J. & Walther A. Genome evolution in the *eremothecium* clade of the *Saccharomyces* complex revealed by comparative genomics. G3 (Bethesda) 1, 539–48 (2011). 22384365 10.1534/g3.111.001032 PMC3276169

[b13] Morales L. *et al.* Complete DNA sequence of *Kuraishia capsulata* illustrates novel genomic features among budding yeasts (Saccharomycotina). Genome Biol Evol 5, 2524–39 (2013). 24317973 10.1093/gbe/evt201 PMC3879985

[b14] Ramezani-Rad M. *et al.* The *Hansenula polymorpha* (strain CBS 4732) genome sequencing and analysis. FEMS Yeast Res 4, 207–15 (2003). 14613885 10.1016/S1567-1356(03)00125-9

[b15] Ravin N. V. *et al.* Genome sequence and analysis of methylotrophic yeast *Hansenula polymorpha* DL1. BMC Genomics 14, 837 (2013). 24279325 10.1186/1471-2164-14-837 PMC3866509

[b16] Woolfit M., Rozpedowska E., Piskur J. & Wolfe K. H. Genome survey sequencing of the wine spoilage yeast *Dekkera* (*Brettanomyces*) *bruxellensis*. Eukaryot Cell 6, 721–33 (2007). 17277171 10.1128/EC.00338-06 PMC1865652

[b17] Wolfe K. H. & Shields D. C. Molecular evidence for an ancient duplication of the entire yeast genome. Nature 387, 708–13 (1997). 9192896 10.1038/42711

[b18] De Hoog G. & Smith M. Ribosomal gene phylogeny and species delimitation in *Geotrichum* and its teleomorphs Studies in Mycologie 50, 489–515 (2004).

[b19] Kurtzman C. P. & Robnett C. J. Relationships among genera of the Saccharomycotina (Ascomycota) from multigene phylogenetic analysis of type species. FEMS Yeast Res 13, 23–33 (2013). 22978764 10.1111/1567-1364.12006

[b20] Pottier I., Gente S., Vernoux J. P. & Gueguen M. Safety assessment of dairy microorganisms: Geotrichum candidum. Int J Food Microbiol 126, 327–32 (2008). 10.1016/j.ijfoodmicro.2007.08.021 17869364

[b21] Boutrou R. & Gueguen M. Interests in *Geotrichum candidum* for cheese technology. Int J Food Microbiol 102, 1–20 (2005). 15924999 10.1016/j.ijfoodmicro.2004.12.028

[b22] Linko M., Haikara A., Ritala A. & Penttilä M. Recent advances in the malting and brewing industry. J Biotechnol 65, 85–98 (1998).

[b23] Bertolini M. C. *et al.* Polymorphism in the lipase genes of *Geotrichum candidum* strains. Eur J Biochem 219, 119–25 (1994). 8306978 10.1111/j.1432-1033.1994.tb19921.x

[b24] Barnett J. A., Payne R. W. & Yarrow D. Yeasts: characteristics and Identification, (Cambridge University Press, Cambridge, 2000).

[b25] Kurtzman C. P. & Fell J. W. (eds.) . The yeasts, a taxonomic study, (Elsevier, Amsterdam, 1998).

[b26] Wouters J., Ayad E., Hugenholtz J. & Smit G. Microbes from raw milk for fermented dairy products. Inter Dairy J 12, 91–109 (2002).

[b27] DeLuna A. *et al.* Exposing the fitness contribution of duplicated genes. Nat Genet 40, 676–81 (2008). 18408719 10.1038/ng.123

[b28] Fares M. A., Keane O. M., Toft C., Carretero-Paulet L. & Jones G. W. The roles of whole-genome and small-scale duplications in the functional specialization of *Saccharomyces cerevisiae* genes. PLoS Genet 9, e1003176 (2013). 23300483 10.1371/journal.pgen.1003176 PMC3536658

[b29] Grassi L. *et al.* Identity and divergence of protein domain architectures after the yeast whole-genome duplication event. Mol Biosyst 6, 2305–15 (2010). 20820472 10.1039/c003507f

[b30] Kaganovich M. & Snyder M. Phosphorylation of yeast transcription factors correlates with the evolution of novel sequence and function. J Proteome Res 11, 261–8 (2012). 22141333 10.1021/pr201065k PMC4077355

[b31] Presser A., Elowitz M. B., Kellis M. & Kishony R. The evolutionary dynamics of the *Saccharomyces cerevisiae* protein interaction network after duplication. Proc Natl Acad Sci U S A 105, 950–4 (2008). 18199840 10.1073/pnas.0707293105 PMC2242688

[b32] Scannell D. R. & Wolfe K. H. A burst of protein sequence evolution and a prolonged period of asymmetric evolution follow gene duplication in yeast. Genome Res 18, 137–47 (2008). 18025270 10.1101/gr.6341207 PMC2134778

[b33] van Hoek M. J. & Hogeweg P. Metabolic adaptation after whole genome duplication. Mol Biol Evol 26, 2441–53 (2009). 19625390 10.1093/molbev/msp160

[b34] Fitzpatrick D. A. Horizontal gene transfer in fungi. FEMS Microbiol Lett 329, 1–8 (2012). 22112233 10.1111/j.1574-6968.2011.02465.x

[b35] Keeling P. J. & Palmer J. D. Horizontal gene transfer in eukaryotic evolution. Nat Rev Genet 9, 605–18 (2008). 18591983 10.1038/nrg2386

[b36] Marcet-Houben M. & Gabaldon T. Acquisition of prokaryotic genes by fungal genomes. Trends Genet 26, 5–8 (2010). 19969385 10.1016/j.tig.2009.11.007

[b37] Aguileta G. *et al.* Assessing the performance of single-copy genes for recovering robust phylogenies. Syst Biol 57, 613–27 (2008). 18709599 10.1080/10635150802306527

[b38] Neuveglise C., Marck C. & Gaillardin C. The intronome of budding yeasts. C R Biol 334, 662–70 (2011). 21819948 10.1016/j.crvi.2011.05.015

[b39] Butler E. E. & Petersen L. J. Sexual reproduction on *Geotrichum candidum*. Science 169, 481–2 (1970). 5463862 10.1126/science.169.3944.481

[b40] Huerta-Cepas J., Capella-Gutierrez S., Pryszcz L. P., Marcet-Houben M. & Gabaldon T. PhylomeDB v4: zooming into the plurality of evolutionary histories of a genome. Nucleic Acids Res 42, D897–902 (2014). 24275491 10.1093/nar/gkt1177 PMC3964985

[b41] Hauser M. *et al.* A transcriptome analysis of isoamyl alcohol-induced filamentation in yeast reveals a novel role for Gre2p as isovaleraldehyde reductase. FEMS Yeast Res 7, 84–92 (2007). 16999827 10.1111/j.1567-1364.2006.00151.x

[b42] Warringer J. & Blomberg A. Involvement of yeast YOL151W/*GRE2* in ergosterol metabolism. Yeast 23, 389–98 (2006). 16598690 10.1002/yea.1363

[b43] Shimada Y., Sugihara A., Tominaga Y., Iizumi T. & Tsunasawa S. cDNA molecular cloning of *Geotrichum candidum* lipase. J Biochem 106, 383–8 (1989). 2481674 10.1093/oxfordjournals.jbchem.a122862

[b44] Hebert A., Casaregola S. & Beckerich J. M. Biodiversity in sulfur metabolism in hemiascomycetous yeasts. FEMS Yeast Res 11, 366–78 (2011). 21348937 10.1111/j.1567-1364.2011.00725.x

[b45] Arfi K., Landaud S. & Bonnarme P. Evidence for distinct L-methionine catabolic pathways in the yeast *Geotrichum candidum* and the bacterium *Brevibacterium linens*. Appl Environ Microbiol 72, 2155–62 (2006). 16517666 10.1128/AEM.72.3.2155-2162.2006 PMC1393222

[b46] Couturier M. *et al.* A thermostable GH45 endoglucanase from yeast: impact of its atypical multimodularity on activity. Microb Cell Fact 10, 103 (2011). 22145993 10.1186/1475-2859-10-103 PMC3247070

[b47] Blanco P., Sieiro C., Reboredo N. M. & Villa T. G. Cloning, molecular characterization, and expression of an endo-polygalacturonase-encoding gene from *Saccharomyces cerevisiae* IM1-8b. FEMS Microbiol Lett 164, 249–55 (1998). 9682473 10.1111/j.1574-6968.1998.tb13094.x

[b48] Gognies S., Gainvors A., Aigle M. & Belarbi A. Cloning, sequence analysis and overexpression of a *Saccharomyces cerevisiae* endopolygalacturonase-encoding gene (*PGL1*). Yeast 15, 11–22 (1999). 10028181 10.1002/(SICI)1097-0061(19990115)15:1<11::AID-YEA336>3.0.CO;2-O

[b49] van den Brink J. & de Vries R. P. Fungal enzyme sets for plant polysaccharide degradation. Appl Microbiol Biotechnol 91, 1477–92 (2011). 21785931 10.1007/s00253-011-3473-2 PMC3160556

[b50] Andersson J. O. Gene transfer and diversification of microbial eukaryotes. Annu Rev Microbiol 63, 177–93 (2009). 19575565 10.1146/annurev.micro.091208.073203

[b51] Syvanen M. Evolutionary implications of horizontal gene transfer. Annu Rev Genet 46, 341–58 (2012). 22934638 10.1146/annurev-genet-110711-155529

[b52] Liti G., Barton D. B. & Louis E. J. Sequence diversity, reproductive isolation and species concepts in *Saccharomyces*. Genetics 174, 839–50 (2006). 16951060 10.1534/genetics.106.062166 PMC1602076

[b53] Novo M. *et al.* Eukaryote-to-eukaryote gene transfer events revealed by the genome sequence of the wine yeast Saccharomyces cerevisiae EC1118. Proc Natl Acad Sci U S A 106, 16333–8 (2009). 19805302 10.1073/pnas.0904673106 PMC2740733

[b54] Gladieux P. *et al.* Fungal evolutionary genomics provides insight into the mechanisms of adaptive divergence in eukaryotes. Mol Ecol 23, 753–73 (2014). 24341913 10.1111/mec.12631

[b55] Wilcox L. J. *et al.* Transcriptional profiling identifies two members of the ATP-binding cassette transporter superfamily required for sterol uptake in yeast. J Biol Chem 277, 32466–72 (2002). 12077145 10.1074/jbc.M204707200

[b56] Hall C., Brachat S. & Dietrich F. S. Contribution of horizontal gene transfer to the evolution of *Saccharomyces cerevisiae*. Eukaryot Cell 4, 1102–15 (2005). 15947202 10.1128/EC.4.6.1102-1115.2005 PMC1151995

[b57] Breuer U. & Harms H. *Debaryomyces hansenii*--an extremophilic yeast with biotechnological potential. Yeast 23, 415–37 (2006). 16652409 10.1002/yea.1374

[b58] Gori K., Mortensen H. D., Arneborg N. & Jespersen L. Expression of the *GPD1* and *GPP2* orthologues and glycerol retention during growth of *Debaryomyces hansenii* at high NaCl concentrations. Yeast 22, 1213–22 (2005). 16278930 10.1002/yea.1306

[b59] Beopoulos A., Nicaud J. M. & Gaillardin C. An overview of lipid metabolism in yeasts and its impact on biotechnological processes. Appl Microbiol Biotechnol 90, 1193–206 (2011). 21452033 10.1007/s00253-011-3212-8

[b60] Swennen D. & Beckerich J. M. *Yarrowia lipolytica* vesicle-mediated protein transport pathways. BMC Evol Biol 7, 219 (2007). 17997821 10.1186/1471-2148-7-219 PMC2241642

[b61] Chebenova-Turcovska V., Zenisova K., Kuchta T., Pangallo D. & Brezna B. Culture-independent detection of microorganisms in traditional Slovakian bryndza cheese. Int J Food Microbiol 150, 73–8 (2011). 21849217 10.1016/j.ijfoodmicro.2011.07.020

[b62] Giannino M. L., Buffoni J. N., Massone E. & Feligini M. Internal transcribed spacer as a target to assess yeast biodiversity in Italian Taleggio PDO cheese. J Food Sci 76, M511–4 (2011). 22417557 10.1111/j.1750-3841.2011.02288.x

[b63] Riquelme M. *et al.* Architecture and development of the *Neurospora crassa* hypha -- a model cell for polarized growth. *Fungal* Biol 115, 446–74 (2011). 10.1016/j.funbio.2011.02.008 21640311

[b64] Casaregola S., Weiss S. & Morel G. New perspectives in hemiascomycetous yeast taxonomy. C R Biol 334, 590–8 (2011). 21819939 10.1016/j.crvi.2011.05.006

[b65] Morales L. & Dujon B. Evolutionary role of interspecies hybridization and genetic exchanges in yeasts. Microbiol Mol Biol Rev 76, 721–39 (2012). 23204364 10.1128/MMBR.00022-12 PMC3510521

[b66] Sipiczki M. Interspecies hybridization and recombination in *Saccharomyces* wine yeasts. FEMS Yeast Res 8, 996–1007 (2008). 18355270 10.1111/j.1567-1364.2008.00369.x

[b67] Mira N. P. *et al.* The genome sequence of the highly acetic acid-tolerant *Zygosaccharomyces bailii*-derived interspecies hybrid strain ISA 1307, isolated from a sparkling wine plant. DNA Res (2014). 10.1093/dnares/dst058 PMC4060950 24453040

[b68] Gabaldon T. & Huynen M. A. Lineage-specific gene loss following mitochondrial endosymbiosis and its potential for function prediction in eukaryotes. Bioinformatics 21 Suppl 2 ii144–50 (2005). 16204094 10.1093/bioinformatics/bti1124

[b69] Aravind L., Watanabe H., Lipman D. J. & Koonin E. V. Lineage-specific loss and divergence of functionally linked genes in eukaryotes. Proc Natl Acad Sci U S A 97, 11319–24 (2000). 11016957 10.1073/pnas.200346997 PMC17198

[b70] Cisse O. H., Pagni M. & Hauser P. M. Comparative genomics suggests that the human pathogenic fungus *Pneumocystis jirovecii* acquired obligate biotrophy through gene loss. Genome Biol Evol 6, 1938–48 (2014). 25062922 10.1093/gbe/evu155 PMC4159005

[b71] On T. *et al.* The evolutionary landscape of the chromatin modification machinery reveals lineage specific gains, expansions, and losses. Proteins 78, 2075–89 (2010). 20455264 10.1002/prot.22723

[b72] Spanu P. D. *et al.* Genome expansion and gene loss in powdery mildew fungi reveal tradeoffs in extreme parasitism. Science 330, 1543–6 (2010). 21148392 10.1126/science.1194573

[b73] Arvas M. *et al.* Comparison of protein coding gene contents of the fungal phyla Pezizomycotina and Saccharomycotina. BMC Genomics 8, 325 (2007). 17868481 10.1186/1471-2164-8-325 PMC2045113

[b74] Braun E. L., Halpern A. L., Nelson M. A. & Natvig D. O. Large-scale comparison of fungal sequence information: mechanisms of innovation in *Neurospora crassa* and gene loss in *Saccharomyces cerevisiae*. Genome Res 10, 416–30 (2000). 10779483 10.1101/gr.10.4.416

[b75] Cornell M. J. *et al.* Comparative genome analysis across a kingdom of eukaryotic organisms: specialization and diversification in the fungi. Genome Res 17, 1809–22 (2007). 17984228 10.1101/gr.6531807 PMC2099590

[b76] Wapinski I., Pfeffer A., Friedman N. & Regev A. Natural history and evolutionary principles of gene duplication in fungi. Nature 449, 54–61 (2007). 17805289 10.1038/nature06107

[b77] McGrath C. L., Gout J. F., Johri P., Doak T. G. & Lynch M. Differential retention and divergent resolution of duplicate genes following whole-genome duplication. Genome Res 24, 1665–75 (2014). 25085612 10.1101/gr.173740.114 PMC4199370

[b78] Donoghue M. T., Keshavaiah C., Swamidatta S. H. & Spillane C. Evolutionary origins of Brassicaceae specific genes in Arabidopsis thaliana. BMC Evol Biol 11, 47 (2011). 21332978 10.1186/1471-2148-11-47 PMC3049755

[b79] Blomme T. *et al.* The gain and loss of genes during 600 million years of vertebrate evolution. Genome Biol 7, R43 (2006). 16723033 10.1186/gb-2006-7-5-r43 PMC1779523

[b80] Foret S. *et al.* New tricks with old genes: the genetic bases of novel cnidarian traits. Trends Genet 26, 154–8 (2010). 20129693 10.1016/j.tig.2010.01.003

[b81] Milde S. *et al.* Characterization of taxonomically restricted genes in a phylum-restricted cell type. Genome Biol 10, R8 (2009). 19161630 10.1186/gb-2009-10-1-r8 PMC2687796

[b82] Johnson B. R. & Tsutsui N. D. Taxonomically restricted genes are associated with the evolution of sociality in the honey bee. BMC Genomics 12, 164 (2011). 21447185 10.1186/1471-2164-12-164 PMC3072959

[b83] Hughes T. R. *et al.* Widespread aneuploidy revealed by DNA microarray expression profiling. Nat Genet 25, 333–7 (2000). 10888885 10.1038/77116

[b84] Fitzpatrick D. A., Logue M. E. & Butler G. Evidence of recent interkingdom horizontal gene transfer between bacteria and *Candida parapsilosis*. BMC Evol Biol 8, 181 (2008). 18577206 10.1186/1471-2148-8-181 PMC2459174

[b85] Hall C. & Dietrich F. S. The reacquisition of biotin prototrophy in *Saccharomyces cerevisiae* involved horizontal gene transfer, gene duplication and gene clustering. Genetics 177, 2293–307 (2007). 18073433 10.1534/genetics.107.074963 PMC2219469

[b86] Rolland T., Neuveglise C., Sacerdot C. & Dujon B. Insertion of horizontally transferred genes within conserved syntenic regions of yeast genomes. PLoS One 4, e6515 (2009). 19654869 10.1371/journal.pone.0006515 PMC2715888

[b87] Strope P. K., Nickerson K. W., Harris S. D. & Moriyama E. N. Molecular evolution of urea amidolyase and urea carboxylase in fungi. BMC Evol Biol 11, 80 (2011). 21447149 10.1186/1471-2148-11-80 PMC3073912

[b88] Slot J. C. & Hibbett D. S. Horizontal transfer of a nitrate assimilation gene cluster and ecological transitions in fungi: a phylogenetic study. PLoS One 2, e1097 (2007). 17971860 10.1371/journal.pone.0001097 PMC2040219

[b89] Galeote V. *et al.* FSY1, a horizontally transferred gene in the *Saccharomyces cerevisiae* EC1118 wine yeast strain, encodes a high-affinity fructose/H+ symporter. Microbiology 156, 3754–61 (2010). 20705659 10.1099/mic.0.041673-0

[b90] Dieuleveux V. & Gueguen M. Antimicrobial effects of D-3-phenyllactic acid on *Listeria monocytogenes* in TSB-YE medium, milk, and cheese. J Food Prot 61, 1281–5 (1998). 9798142 10.4315/0362-028x-61.10.1281

[b91] Dieuleveux V., Lemarinier S. & Gueguen M. Antimicrobial spectrum and target site of D-3-phenyllactic acid. Int J Food Microbiol 40, 177–83 (1998). 9620125 10.1016/s0168-1605(98)00031-2

[b92] Dieuleveux V., Van Der Pyl D., Chataud J. & Gueguen M. Purification and characterization of anti-*Listeria* compounds produced by *Geotrichum candidum*. Appl Environ Microbiol 64, 800–3 (1998). 9464426 10.1128/aem.64.2.800-803.1998 PMC106124

[b93] Gente S. *et al.* Intra-species chromosome-length polymorphism in *Geotrichum candidum* revealed by pulsed field gel electrophoresis. Int J Food Microbiol 76, 127–34 (2002). 12038569 10.1016/s0168-1605(02)00023-5

[b94] Gente S., Desmasures N., Panoff J. M. & Gueguen M. Genetic diversity among *Geotrichum candidum* strains from various substrates studied using RAM and RAPD-PCR. J Appl Microbiol 92, 491–501 (2002). 11872125 10.1046/j.1365-2672.2002.01553.x

[b95] Gente S., Sohier D., Coton E., Duhamel C. & Gueguen M. Identification of *Geotrichum candidum* at the species and strain level: proposal for a standardized protocol. J Ind Microbiol Biotechnol 33, 1019–31 (2006). 16855820 10.1007/s10295-006-0130-3

[b96] Leclercq-Perlat M. N., Oumer A., Bergere J. L., Spinnler H. E. & Corrieu G. Behavior of *Brevibacterium linens* and *Debaryomyces hansenii* as ripening flora in controlled production of smear soft cheese from reconstituted milk: growth and substrate consumption dairy foods. J Dairy Sci 83, 1665–73 (2000). 10984141 10.3168/jds.s0022-0302(00)75035-1

[b97] Mansour S., Beckerich J. M. & Bonnarme P. Lactate and amino acid catabolism in the cheese-ripening yeast *Yarrowia lipolytica*. Appl Environ Microbiol 74, 6505–12 (2008). 18776032 10.1128/AEM.01519-08 PMC2576680

[b98] Aury J. M. *et al.* High quality draft sequences for prokaryotic genomes using a mix of new sequencing technologies. BMC Genomics 9, 603 (2008). 19087275 10.1186/1471-2164-9-603 PMC2625371

[b99] Foissac S. *et al.* Genome Annotation in Plants and Fungi: EuGene as a Model Platform. Current Bioinformatics 3 (2008).

[b100] Degroeve S., Saeys Y., De Baets B., Rouze P. & Van de Peer Y. SpliceMachine: predicting splice sites from high-dimensional local context representations. Bioinformatics 21, 1332–8 (2005). 15564294 10.1093/bioinformatics/bti166

[b101] Sterck L., Billiau K., Abeel T., Rouze P. & Van de Peer Y. ORCAE: online resource for community annotation of eukaryotes. Nat Methods 9, 1041 (2012). 23132114 10.1038/nmeth.2242

[b102] Abeel T., Van Parys T., Saeys Y., Galagan J. & Van de Peer Y. GenomeView: a next-generation genome browser. Nucleic Acids Res 40, e12 (2012). 22102585 10.1093/nar/gkr995 PMC3258165

[b103] Grossetete S., Labedan B. & Lespinet O. FUNGIpath: a tool to assess fungal metabolic pathways predicted by orthology. BMC Genomics 11, 81 (2010). 20122162 10.1186/1471-2164-11-81 PMC2829015

[b104] Edgar R. C. MUSCLE: multiple sequence alignment with high accuracy and high throughput. Nucleic Acids Res 32, 1792–7 (2004). 15034147 10.1093/nar/gkh340 PMC390337

[b105] Castresana J. Selection of conserved blocks from multiple alignments for their use in phylogenetic analysis. Mol Biol Evol 17, 540–52 (2000). 10742046 10.1093/oxfordjournals.molbev.a026334

[b106] Guindon S. & Gascuel O. A simple, fast, and accurate algorithm to estimate large phylogenies by maximum likelihood. Syst Biol 52, 696–704 (2003). 14530136 10.1080/10635150390235520

[b107] Perriere G. & Gouy M. WWW-query: an on-line retrieval system for biological sequence banks. Biochimie 78, 364–9 (1996). 8905155 10.1016/0300-9084(96)84768-7

[b108] Drillon G., Carbone A. & Fischer G. SynChro: a fast and easy tool to reconstruct and visualize synteny blocks along eukaryotic chromosomes. PLoS One 9, e92621 (2014). 24651407 10.1371/journal.pone.0092621 PMC3961402

[b109] Huerta-Cepas J. *et al.* PhylomeDB v3.0: an expanding repository of genome-wide collections of trees, alignments and phylogeny-based orthology and paralogy predictions. Nucleic Acids Res 39, D556–60 (2011). 21075798 10.1093/nar/gkq1109 PMC3013701

[b110] Katoh K., Kuma K., Toh H. & Miyata T. MAFFT version 5: improvement in accuracy of multiple sequence alignment. Nucleic Acids Res 33, 511–8 (2005). 15661851 10.1093/nar/gki198 PMC548345

[b111] Lassmann T. & Sonnhammer E. L. Kalign--an accurate and fast multiple sequence alignment algorithm. BMC Bioinformatics 6, 298 (2005). 16343337 10.1186/1471-2105-6-298 PMC1325270

[b112] Landan G. & Graur D. Heads or tails: a simple reliability check for multiple sequence alignments. Mol Biol Evol 24, 1380–3 (2007). 17387100 10.1093/molbev/msm060

[b113] Wallace I. M., O’Sullivan O., Higgins D. G. & Notredame C. M-Coffee: combining multiple sequence alignment methods with T-Coffee. Nucleic Acids Res 34, 1692–9 (2006). 16556910 10.1093/nar/gkl091 PMC1410914

[b114] Capella-Gutierrez S., Silla-Martinez J. M. & Gabaldon T. trimAl: a tool for automated alignment trimming in large-scale phylogenetic analyses. Bioinformatics 25, 1972–3 (2009). 19505945 10.1093/bioinformatics/btp348 PMC2712344

[b115] Gascuel O. BIONJ: an improved version of the NJ algorithm based on a simple model of sequence data. Mol Biol Evol 14, 685–95 (1997). 9254330 10.1093/oxfordjournals.molbev.a025808

[b116] Akaike H. Information theory and extension of the maximum likelihood principle. Proceedings of the 2nd international symposium on information theory, 267–281 (1973).

[b117] Guindon S. *et al.* New algorithms and methods to estimate maximum-likelihood phylogenies: assessing the performance of PhyML 3.0. Syst Biol 59, 307–21 (2010). 20525638 10.1093/sysbio/syq010

[b118] Stamatakis A., Ludwig T. & Meier H. RAxML-III: a fast program for maximum likelihood-based inference of large phylogenetic trees. Bioinformatics 21, 456–63 (2005). 15608047 10.1093/bioinformatics/bti191

[b119] Wehe A., Bansal M. S., Burleigh J. G. & Eulenstein O. DupTree: a program for large-scale phylogenetic analyses using gene tree parsimony. Bioinformatics 24, 1540–1 (2008). 18474508 10.1093/bioinformatics/btn230

[b120] Gabaldon T. Comparative genomics-based prediction of protein function. Methods Mol Biol 439, 387–401 (2008). 18370117 10.1007/978-1-59745-188-8_26

[b121] Wu T. D. & Nacu S. Fast and SNP-tolerant detection of complex variants and splicing in short reads. Bioinformatics 26, 873–81 (2010). 20147302 10.1093/bioinformatics/btq057 PMC2844994

[b122] Anders S., Pyl P. T. & Huber W. HTSeq - A Python framework to work with high-throughput sequencing data. bioRxiv, 10.1101/002824 (2014). PMC4287950 25260700

