# Supplementary material for: Differential gene retention as an evolutionary mechanism to generate biodiversity and adaptation in yeasts
Source: Sci Rep. 2015 Jun 25;5:11571. doi: 10.1038/srep11571 (PMC4479816; doi:10.1038/srep11571)
Supplement: Supplementary Information [file srep11571-s1.pdf]

Differential gene retention as an evolutionary mechanism to generate biodiversity and adaptation in yeasts

Guillaume Morel, Lieven Sterck, Dominique Swennen, Marina Marcet-Houben, Djamila Onesime, Anthony Levasseur, Noémie Jacques, Sandrine Mallet, Arnaux Couloux., Karine Labadie, Joëlle Amselem, Jean-Marie Beckerich, Bernard Henrissat, Yves Van de Peer, Patrick Wincker, Jean-Luc Souciet, Toni Gabaldón, Colin R. Tinsley & Serge Casaregola

Supplementary figures

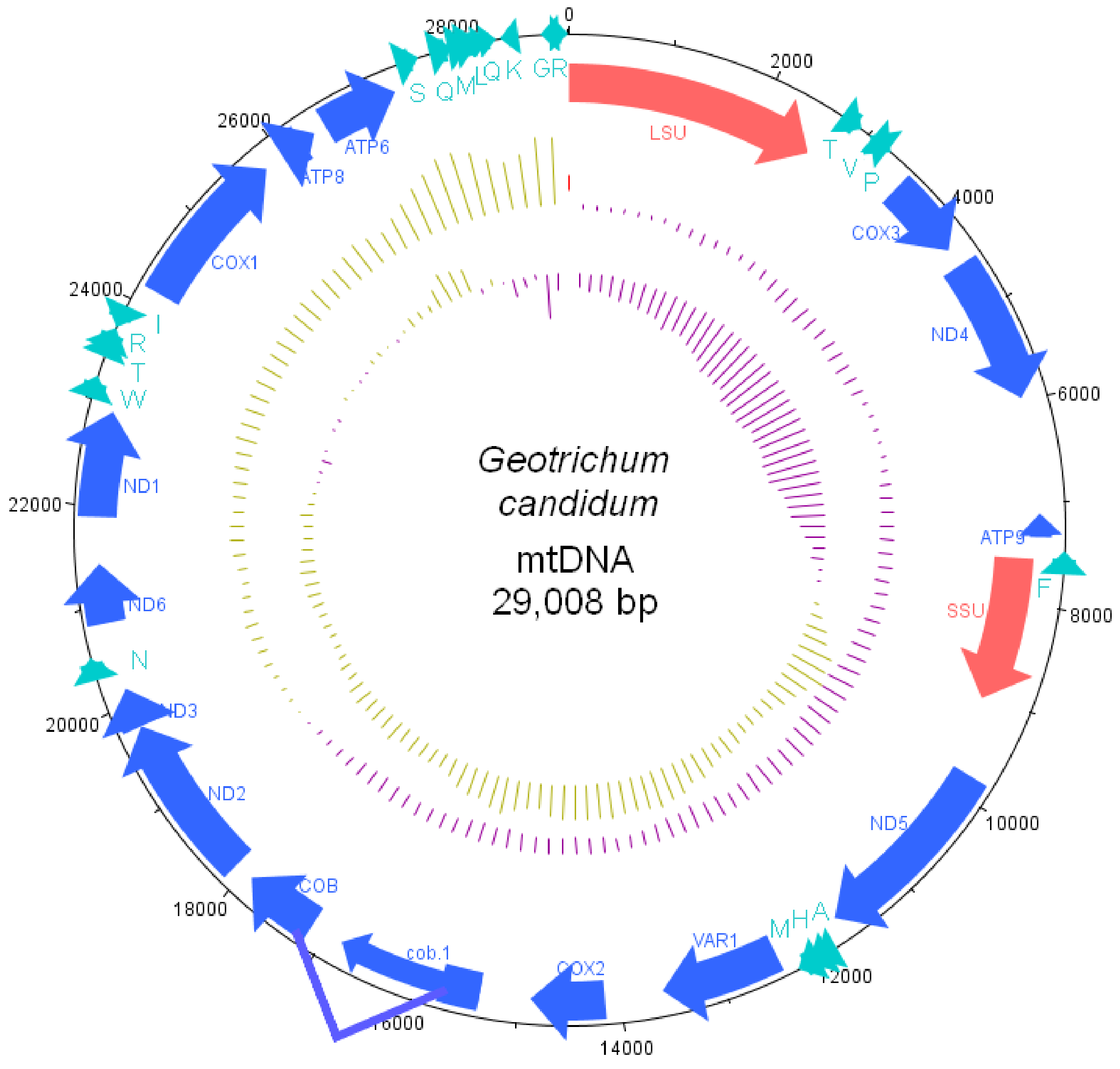

Supplementary Figure S1. **Circular map of *Geotrichum candidum* mtDNA.** Map was drawn using dnaplotter (https://www.sanger.ac.uk/.../dnaplotter/). Protein coding gene and ribosomal gene models were detected using blastX against the available Saccharomycotina mtDNAs. tRNA genes were detected using tRNAscan-SE. Protein coding genes are indicated by large blue arrows, rDNA genes by red arrows, tRNAs by green arrows with charged amino acid indicated in one letter code. The intronic ORFcob.1 is indicated by a thin blue arrow and the link between the two COB exons is shown in blue. GC% variations (outer bars) and GC skew (inner bars) are indicated in mauve (below average) and green (above average).

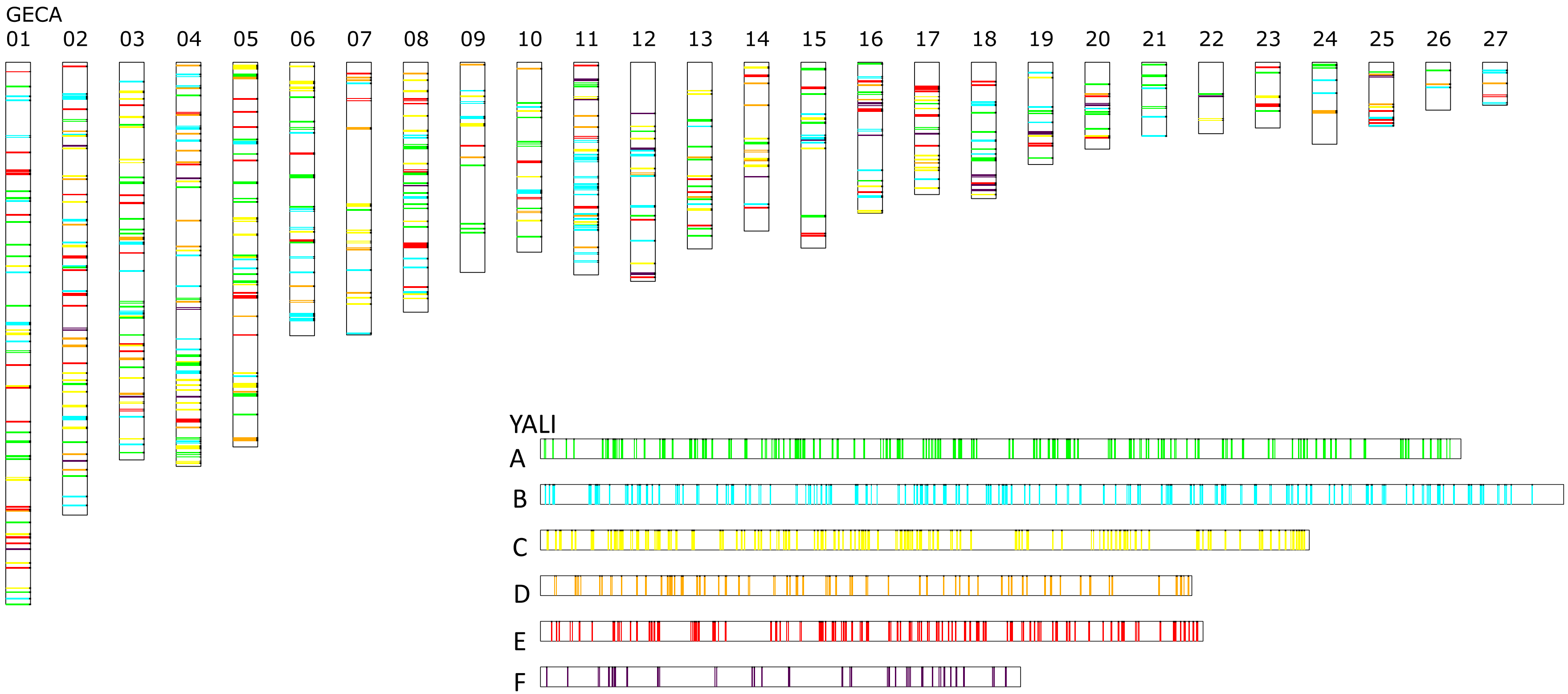

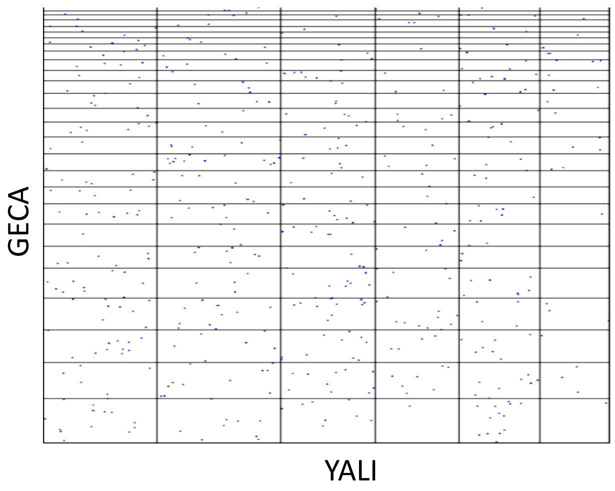

Supplementary Figure S2a. **Synteny between *G. candidum* (GECA) and *Y. lipolytica* (YALI)**. Top: distribution in the GECA scaffolds of YALI orthologs defined on the basis of bidirectional hits in blastp comparison. Each color is attributed to the genes of one YALI chromosome. Right: dotplot representation.

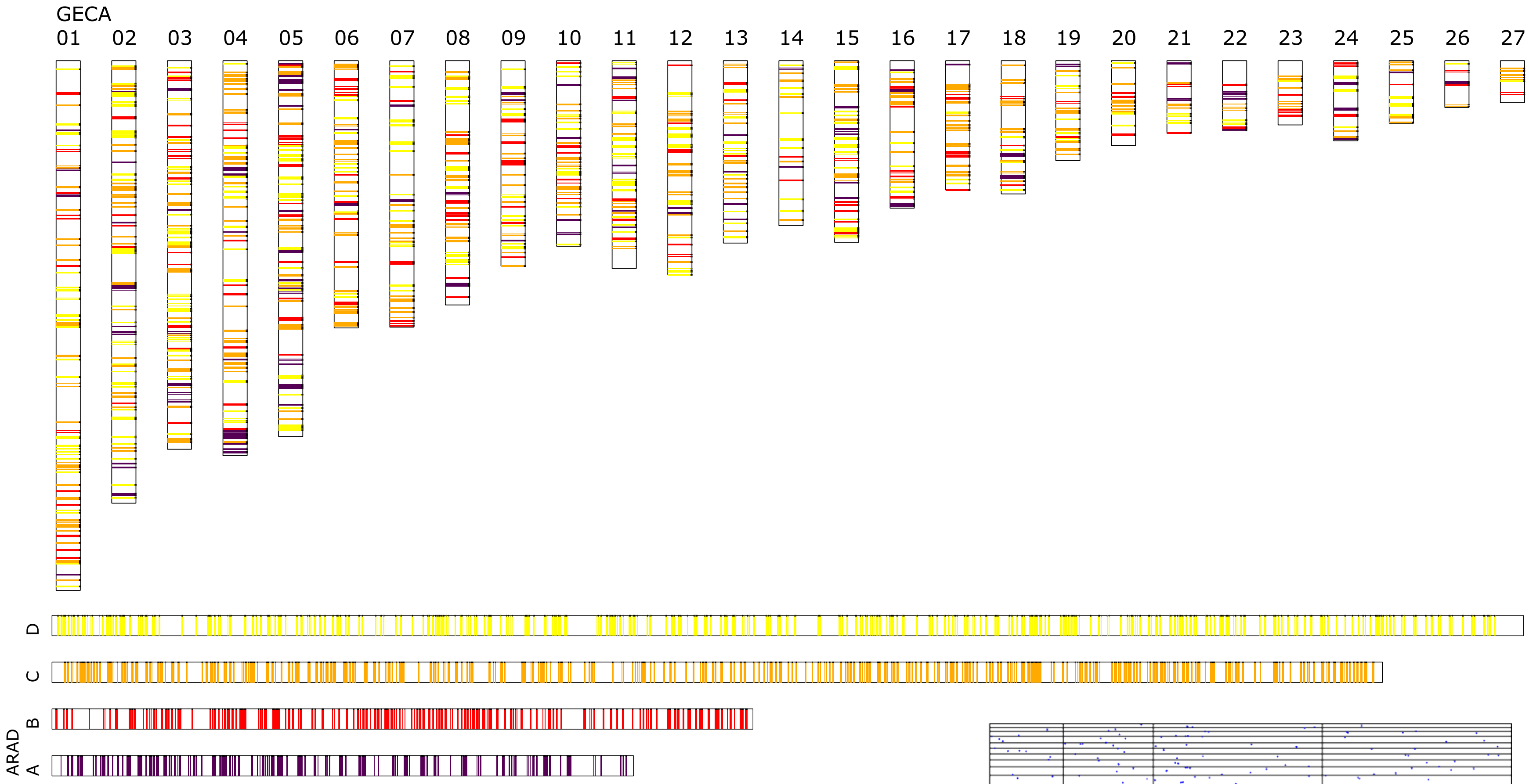

Supplementary Figure S2b. **Synteny between *G. candidum* (GECA) and *B. adeninivorans* (ARAD)**. Top: distribution in the GECA scaffolds of ARAD orthologs defined on the basis of bidirectional hits in blastp comparison Each color is attributed to the genes of one ARAD chromosome. Right: dotplot representation.

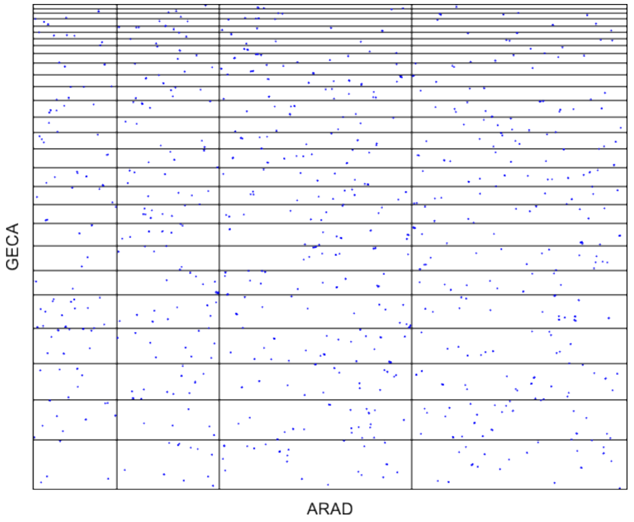

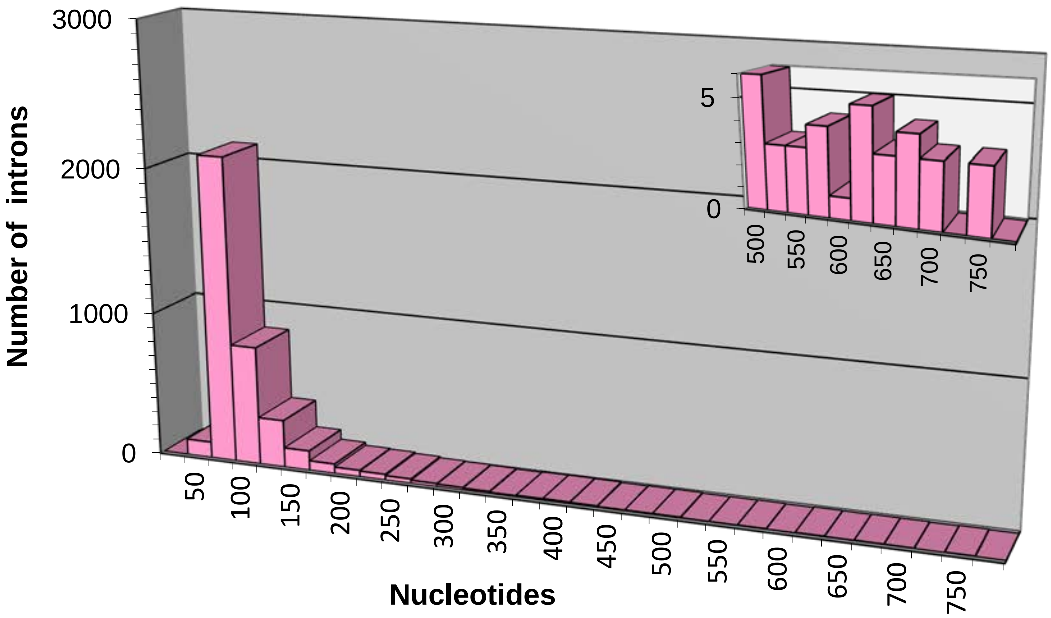

Supplementary Figure S3. **Distribution of intron length in *G. candidum*.** The number of introns is plotted against their length in nucleotides. The inset is an expansion of the higher end of the intron length distribution, demonstrating a continuation of the distribution by a small number of unusually long introns.

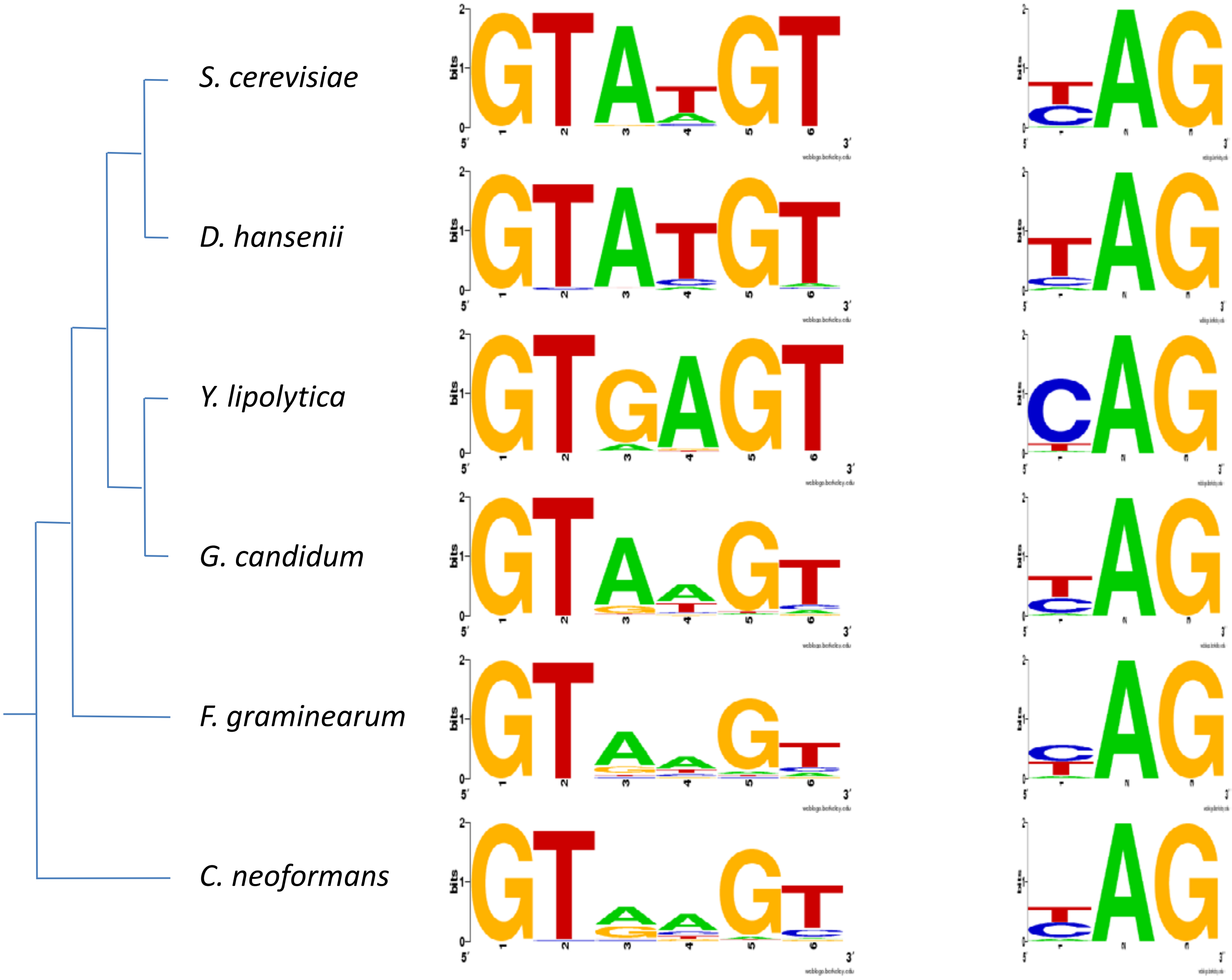

Supplementary Figure S4. **Conservation of 5’ (left) and 3’ (right) splice sites in *G. candidum* and various yeasts and fungi**. The consensus sequences were obtained using weblogo (http://weblogo.berkeley.edu/) version 2.8.2 . The sequences from S. cerevisiae were extracted from the Saccharomyces Genome Database (http://www.yeastgenome.org/) and those from *Y. lipolytica*, *D. hansenii*, *F. graminearum* and *C. neoformans* from NCBI (http://www.ncbi.nlm.nih.gov/genome/).

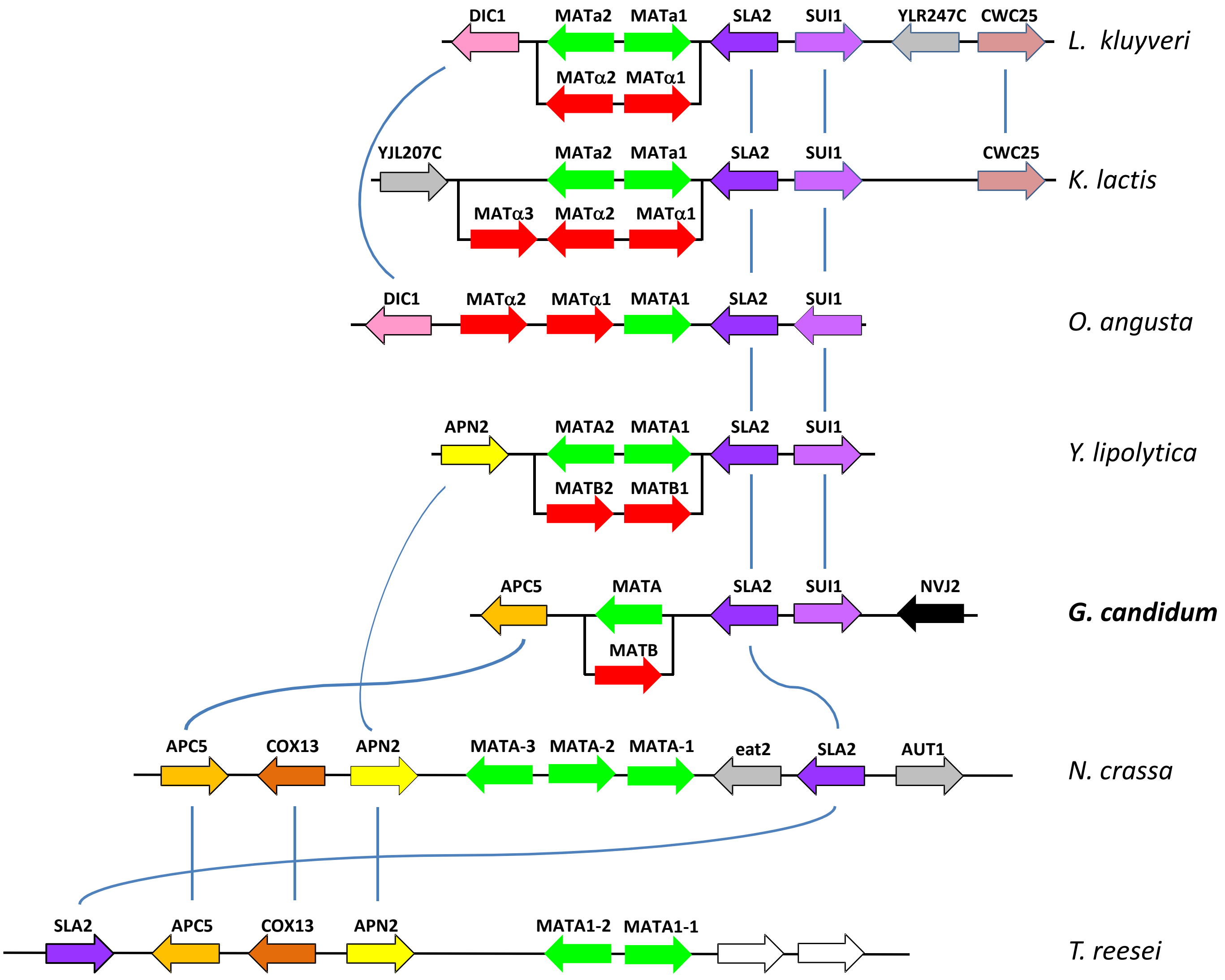

Supplementary Figure S5. **Organization of the mating type loci and their surroundings in *G. candidum* and in six fungal species.** Gene names are indicated above the genes. Orthologs are represented with the same color. MATA(a) genes are represented in green, MATB(α) genes are represented in red. Orientation of genes on chromosomes (black lines) are indicated. Data are from Butler *et al.* (2004) and Seidl *et al.* (2009).

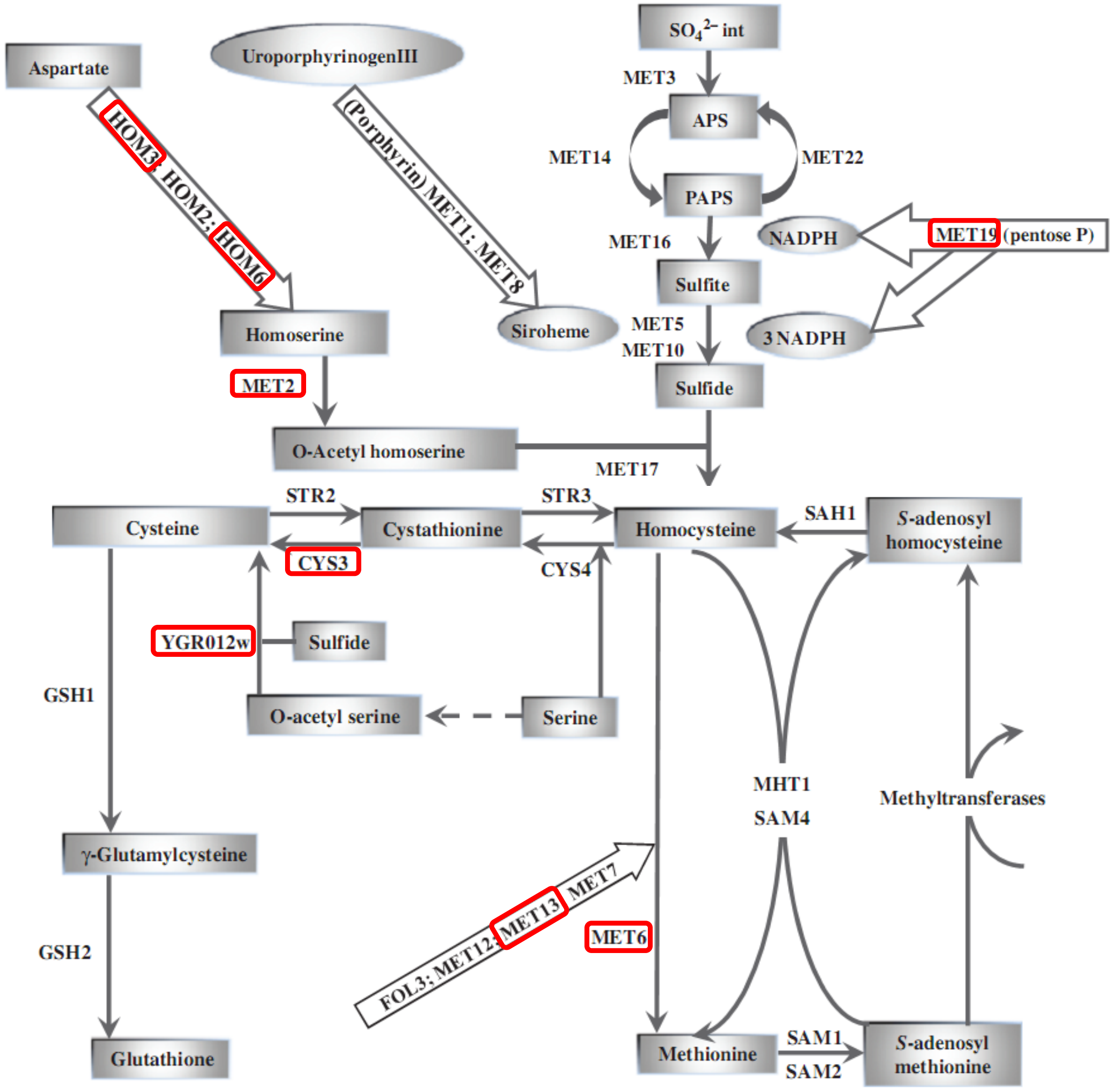

Supplementary Figure S6. **Duplicated genes of the sulfur metabolism pathway in *G. candidum*.** The scheme was taken from Hebert *et al.* (2011). Duplicated genes in *G. candidum* are boxed in red.

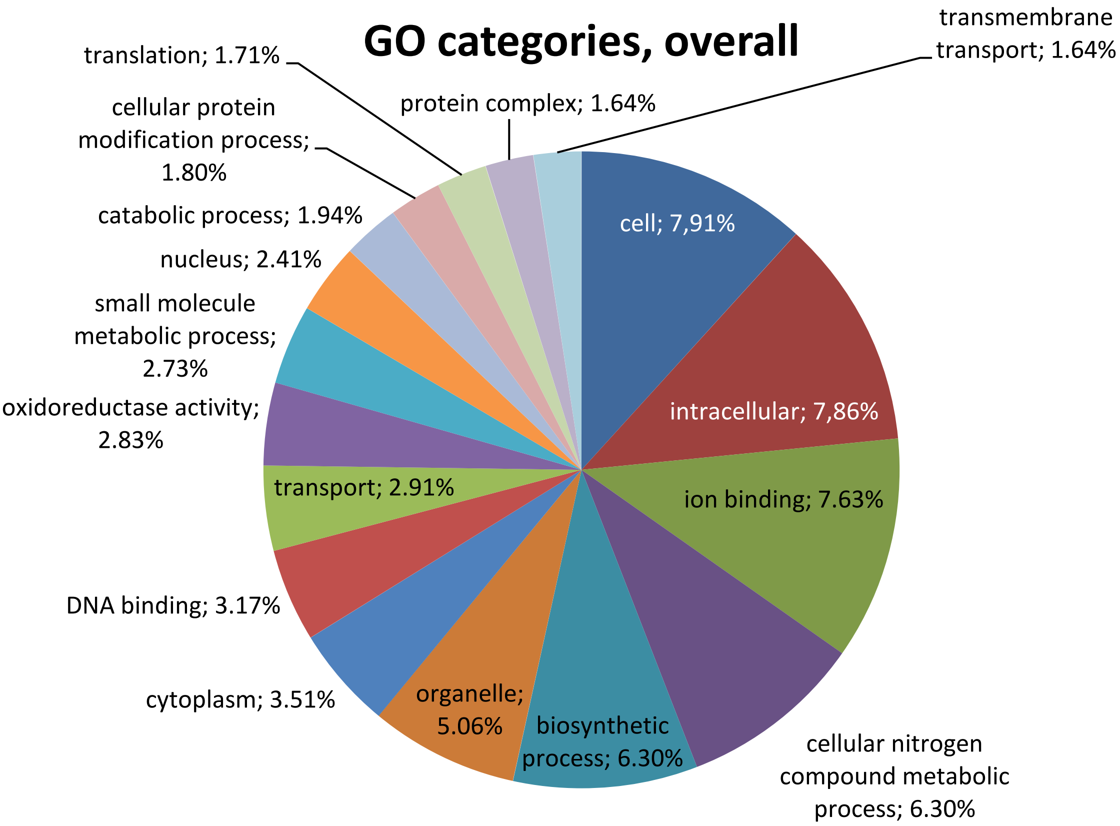

Supplementary Figure S7a. **Overall distribution of predicted functions in the genome of *G. candidum* in Gene ontology categories**.

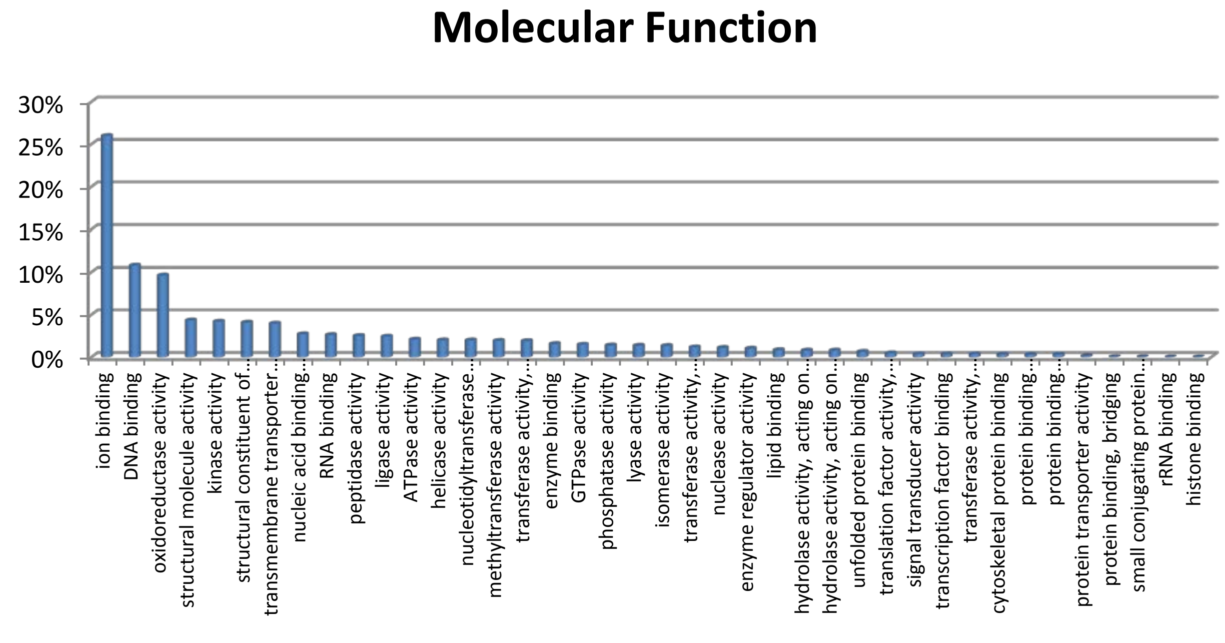

Supplementary Figure S7b. **Distribution of predicted molecular functions in the genome of *G. candidum* in Gene ontology categories.**

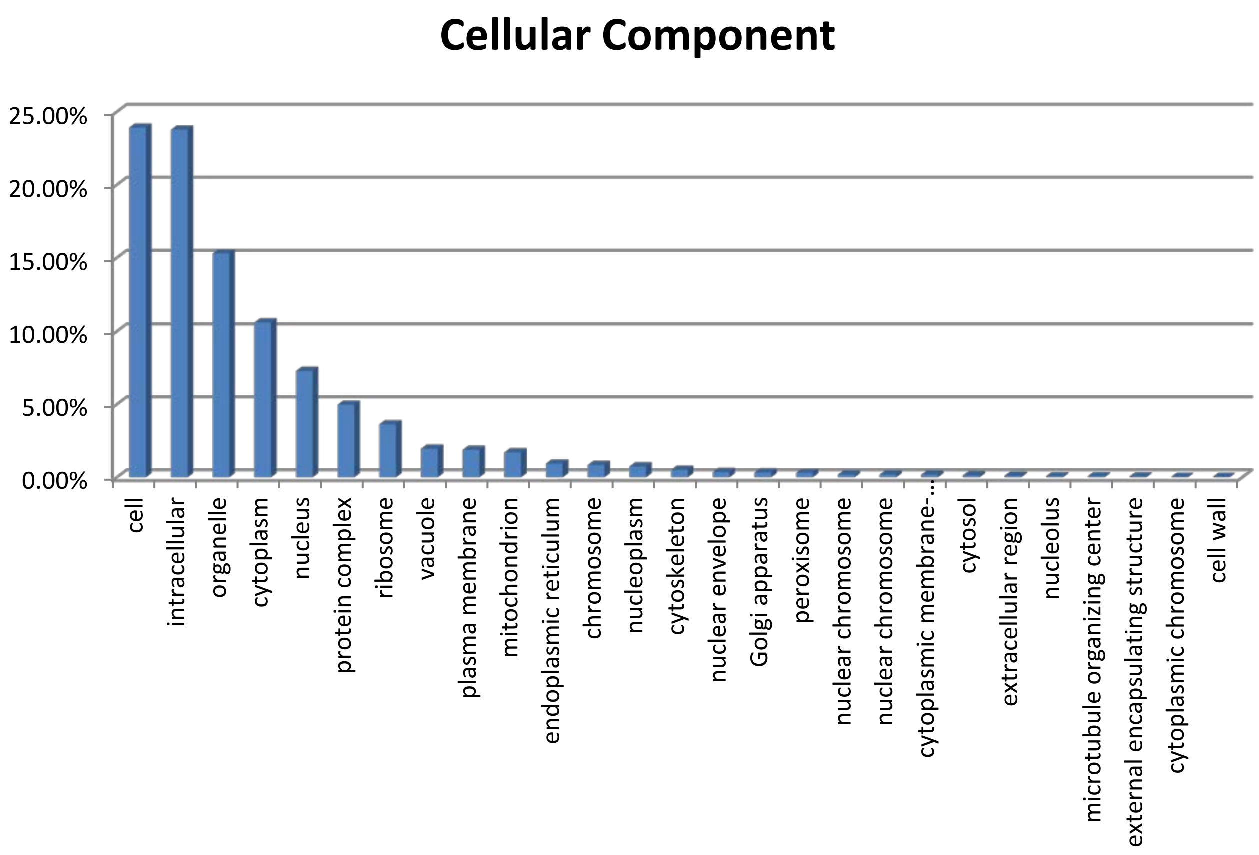

Supplementary Figure S7c. **Distribution of predicted functions in the genome of *G. candidum* in Gene ontology Cellular component categories**.

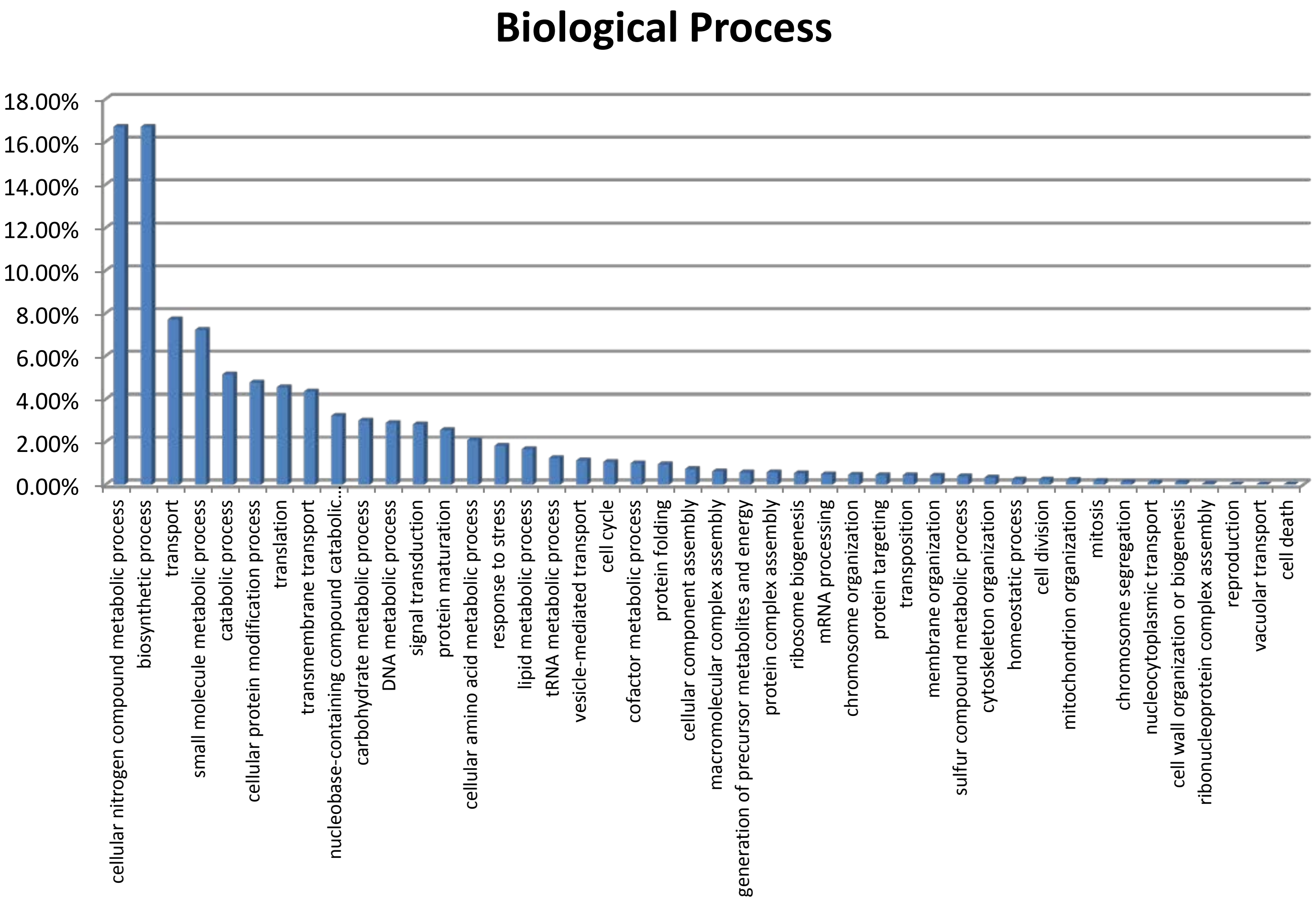

Supplementary Figure S7d. **Distribution of predicted functions in the genome of *G. candidum* in Gene ontology Biological process categories**.

Suplementary Fig S8. **Phylogenetic trees from possible cases of HGT extracted from the G. candidum phylum**.

Fig S8a

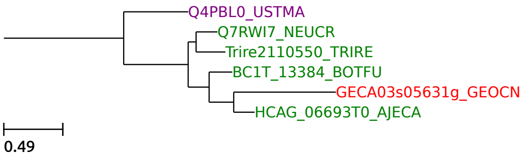

Fig S8b

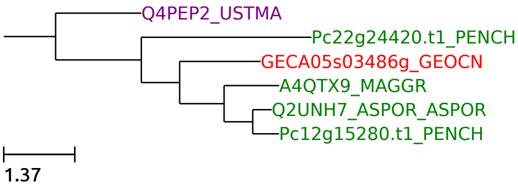

Fig S8c

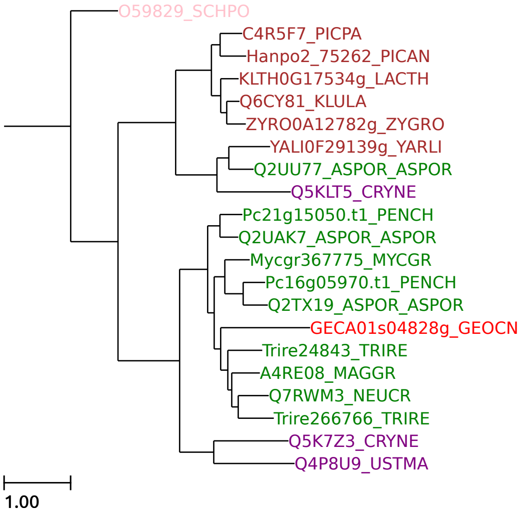

Fig S8d

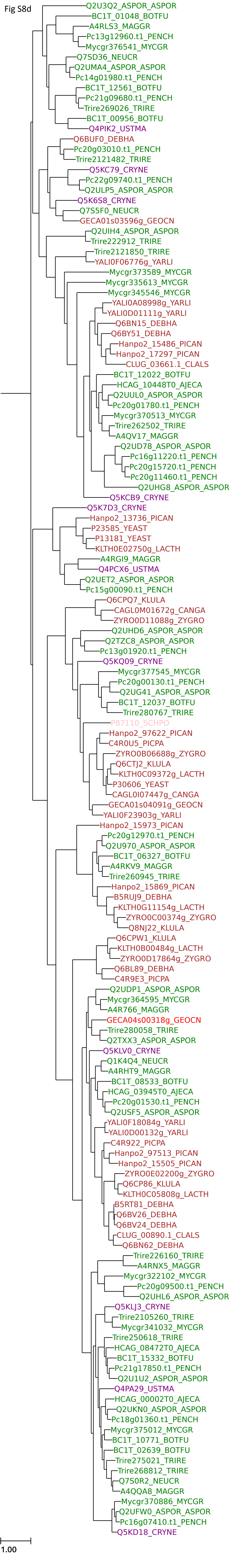
Q2U3Q2_ASPOR_ASPOR
BC1T_01048_BOTFU
A4RLS3_MAGGR
Pc13g12960.t1_PENCH
Mycgr376541_MYCGR
Q7SD36_NEUCR
Q2UMA4_ASPOR_ASPOR
Pc14g01980.t1_PENCH
BC1T_12561_BOTFU
Pc21g09680.t1_PENCH
Trire269026_TRIRE
BC1T_00956_BOTFU
Q4PIK2_USTMA
Q6BUF0_DEBHA
Pc20g03010.t1_PENCH
Trire2121482_TRIRE
Q5KC79_CRYNE
Pc22g09740.t1_PENCH
Q2ULP5_ASPOR_ASPOR
Q5K6S8_CRYNE
Q7S5F0_NEUCR
GECA01s03596g_GEOCN
Q2UIH4_ASPOR_ASPOR
Trire222912_TRIRE
Trire2121850_TRIRE
YALI0F06776g_YARLI
Mycgr373589_MYCGR
Mycgr335613_MYCGR
Mycgr345546_MYCGR
YALI0A08998g_YARLI
YALI0D01111g_YARLI
Q6BN15_DEBHA
Q6BY51_DEBHA
Hanpo2_15486_PICAN
Hanpo2_17297_PICAN
CLUG_03661.1_CLALS
BC1T_12022_BOTFU
HCAG_10448T0_AJECA
Q2UUL0_ASPOR_ASPOR
Pc20g01780.t1_PENCH
Mycgr370513_MYCGR
Trire262502_TRIRE
A4QV17_MAGGR
Q2UD78_ASPOR_ASPOR
Pc16g11220.t1_PENCH
Pc20g15720.t1_PENCH
Pc20g11460.t1_PENCH
Q2UHG8_ASPOR_ASPOR
Q5KCB9_CRYNE
Q5K7D3_CRYNE
Hanpo2_13736_PICAN
P23585_YEAST
P13181_YEAST
KLTH0E02750g_LACTH
A4RGI9_MAGGR
Q4PCX6_USTMA
Q2UET2_ASPOR_ASPOR
Pc15g00090.t1_PENCH
Q6CPQ7_KLULA
CAGL0M01672g_CANGA
ZYRO0D11088g_ZYGRO
Q2UHD6_ASPOR_ASPOR
Q2TZC8_ASPOR_ASPOR
Pc13g01920.t1_PENCH
Q5KQ09_CRYNE
Mycgr377545_MYCGR
Pc20g00130.t1_PENCH
Q2UG41_ASPOR_ASPOR
BC1T_12037_BOTFU
Trire280767_TRIRE
P87110_SCHPO
Hanpo2_97622_PICAN
C4R0U5_PICPA
ZYRO0B06688g_ZYGRO
Q6CTJ2_KLULA
KLTH0C09372g_LACTH
P30606_YEAST
CAGL0I07447g_CANGA
GECA01s04091g_GEOCN
YALI0F23903g_YARLI
Hanpo2_15973_PICAN
Pc20g12970.t1_PENCH
Q2U970_ASPOR_ASPOR
BC1T_06327_BOTFU
A4RKV9_MAGGR
Trire260945_TRIRE
Hanpo2_15869_PICAN
B5RUJ9_DEBHA
KLTH0G11154g_LACTH
ZYRO0C00374g_ZYGRO
Q8NJ22_KLULA
Q6CPW1_KLULA
KLTH0B00484g_LACTH
ZYRO0D17864g_ZYGRO
Q6BL89_DEBHA
C4R9E3_PICPA
Q2UDP1_ASPOR_ASPOR
Mycgr364595_MYCGR
A4R766_MAGGR
GECA04s00318g_GEOCN
Trire280058_TRIRE
Q2TXX3_ASPOR_ASPOR
Q5KLV0_CRYNE
Q1K4Q4_NEUCR
A4RHT9_MAGGR
BC1T_08533_BOTFU
HCAG_03945T0_AJECA
Pc20g01530.t1_PENCH
Q2USF5_ASPOR_ASPOR
YALI0F18084g_YARLI
YALI0D00132g_YARLI
C4R922_PICPA
Hanpo2_97513_PICAN
Hanpo2_15505_PICAN
ZYRO0E02200g_ZYGRO
Q6CP86_KLULA
KLTH0C05808g_LACTH
B5RT81_DEBHA
Q6BV26_DEBHA
Q6BV24_DEBHA
CLUG_00890.1_CLALS
Q6BN62_DEBHA
Trire226160_TRIRE
A4RNX5_MAGGR
Mycgr322102_MYCGR
Pc20g09500.t1_PENCH
Q2UHL6_ASPOR_ASPOR
Q5KLJ3_CRYNE
Trire2105260_TRIRE
Mycgr341032_MYCGR
Trire250618_TRIRE
HCAG_08472T0_AJECA
BC1T_15332_BOTFU
Pc21g17850.t1_PENCH
Q2U1U2_ASPOR_ASPOR
Q4PA29_USTMA
HCAG_00002T0_AJECA
Q2UKN0_ASPOR_ASPOR
Pc18g01360.t1_PENCH
Mycgr375012_MYCGR
BC1T_10771_BOTFU
BC1T_02639_BOTFU
Trire275021_TRIRE
Trire268812_TRIRE
Q7S0R2_NEUCR
A4QQA8_MAGGR
Mycgr370886_MYCGR
Q2UFW0_ASPOR_ASPOR
Pc16g07410.t1_PENCH
Q5KD18_CRYNE
1.00

Fig S8e

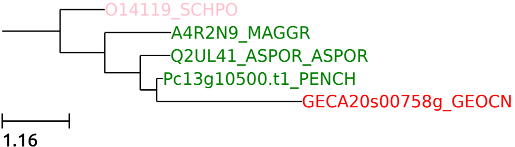
O14119_SCHPO
A4R2N9_MAGGR
Q2UL41_ASPOR_ASPOR
Pc13g10500.t1_PENCH
GECA20s00758g_GEOCN
1.16

Fig S8f

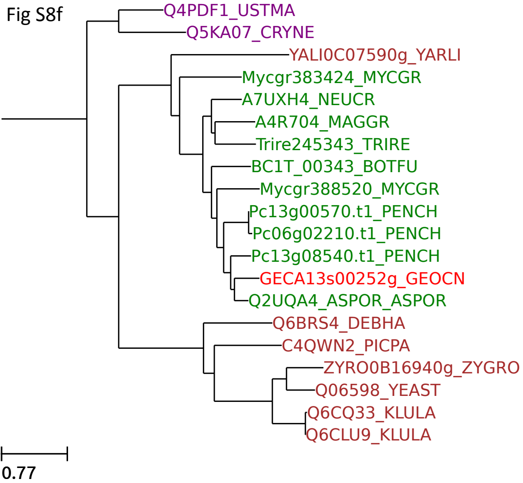
Q4PDF1_USTMA
Q5KA07_CRYNE
YALI0C07590g_YARLI
Mycgr383424_MYCGR
A7UXH4_NEUCR
A4R704_MAGGR
Trire245343_TRIRE
BC1T_00343_BOTFU
Mycgr388520_MYCGR
Pc13g00570.t1_PENCH
Pc06g02210.t1_PENCH
Pc13g08540.t1_PENCH
GECA13s00252g_GEOCN
Q2UQA4_ASPOR_ASPOR
Q6BRS4_DEBHA
C4QWN2_PICPA
ZYRO0B16940g_ZYGRO
Q06598_YEAST
Q6CQ33_KLULA
Q6CLU9_KLULA
0.77

Fig S8g

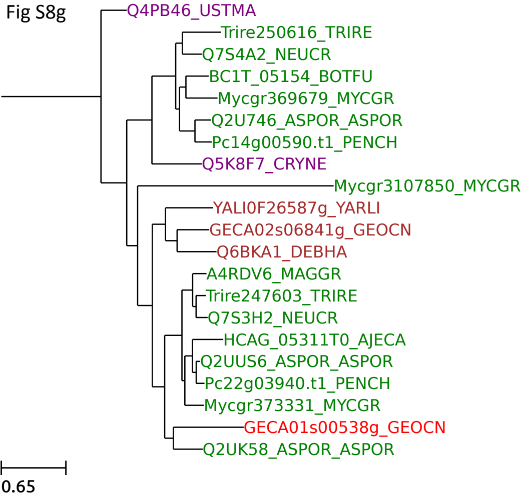
Q4PB46_USTMA
Trire250616_TRIRE
Q7S4A2_NEUCR
BC1T_05154_BOTFU
Mycgr369679_MYCGR
Q2U746_ASPOR_ASPOR
Pc14g00590.t1_PENCH
Q5K8F7_CRYNE
Mycgr3107850_MYCGR
YALI0F26587g_YARLI
GECA02s06841g_GEOCN
Q6BKA1_DEBHA
A4RDV6_MAGGR
Trire247603_TRIRE
Q7S3H2_NEUCR
HCAG_05311T0_AJECA
Q2UUS6_ASPOR_ASPOR
Pc22g03940.t1_PENCH
Mycgr373331_MYCGR
GECA01s00538g_GEOCN
Q2UK58_ASPOR_ASPOR
0.65

Fig S8h

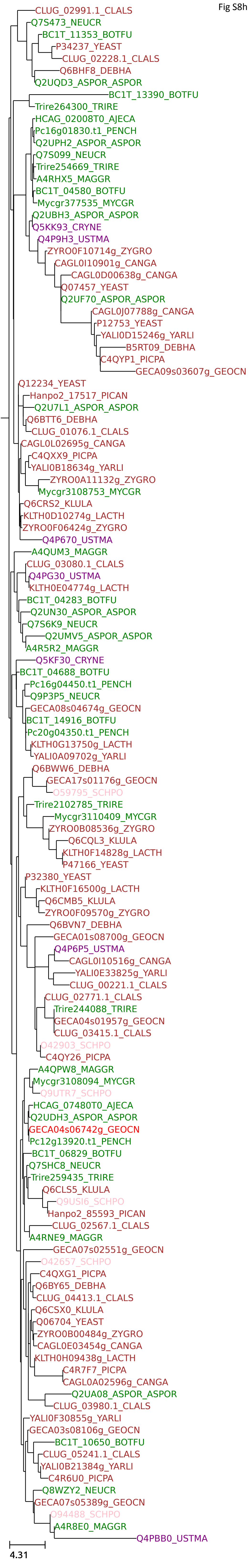
CLUG_02991.1_CLALS
Q7S473_NEUCR
BC1T_11353_BOTFU
P34237_YEAST
CLUG_02228.1_CLALS
Q6BHF8_DEBHA
Q2UQD3_ASPOR_ASPOR
BC1T_13390_BOTFU
Trire264300_TRIRE
HCAG_02008T0_AJECA
Pc16g01830.t1_PENCH
Q2UPH2_ASPOR_ASPOR
Q7S099_NEUCR
Trire254669_TRIRE
A4RHX5_MAGGR
BC1T_04580_BOTFU
Mycgr377535_MYCGR
Q2UBH3_ASPOR_ASPOR
Q5KK93_CRYNE
Q4P9H3_USTMA
ZYRO0F10714g_ZYGRO
CAGL0I10901g_CANGA
CAGL0D00638g_CANGA
Q07457_YEAST
Q2UF70_ASPOR_ASPOR
CAGL0J07788g_CANGA
P12753_YEAST
YALI0D15246g_YARLI
B5RT09_DEBHA
C4QYP1_PICPA
GECA09s03607g_GEOCN
Q12234_YEAST
Hanpo2_17517_PICAN
Q2U7L1_ASPOR_ASPOR
Q6BTT6_DEBHA
CLUG_01076.1_CLALS
CAGL0L02695g_CANGA
C4QXX9_PICPA
YALI0B18634g_YARLI
ZYRO0A11132g_ZYGRO
Mycgr3108753_MYCGR
Q6CRS2_KLULA
KLTH0D10274g_LACTH
ZYRO0F06424g_ZYGRO
Q4P670_USTMA
A4QUM3_MAGGR
CLUG_03080.1_CLALS
Q4PG30_USTMA
KLTH0E04774g_LACTH
BC1T_04283_BOTFU
Q2UN30_ASPOR_ASPOR
Q7S6K9_NEUCR
Q2UMV5_ASPOR_ASPOR
A4R5R2_MAGGR
Q5KF30_CRYNE
BC1T_04688_BOTFU
Pc16g04450.t1_PENCH
Q9P3P5_NEUCR
GECA08s04674g_GEOCN
BC1T_14916_BOTFU
Pc20g04350.t1_PENCH
KLTH0G13750g_LACTH
YALI0A09702g_YARLI
Q6BWW6_DEBHA
GECA17s01176g_GEOCN
O59795_SCHPO
Trire2102785_TRIRE
Mycgr3110409_MYCGR
ZYRO0B08536g_ZYGRO
Q6CQL3_KLULA
KLTH0F14828g_LACTH
P47166_YEAST
P32380_YEAST
KLTH0F16500g_LACTH
Q6CMB5_KLULA
ZYRO0F09570g_ZYGRO
Q6BVN7_DEBHA
GECA01s08700g_GEOCN
Q4P6P5_USTMA
CAGL0I10516g_CANGA
YALI0E33825g_YARLI
CLUG_00221.1_CLALS
CLUG_02771.1_CLALS
Trire244088_TRIRE
GECA04s01957g_GEOCN
CLUG_03415.1_CLALS
O42903_SCHPO
C4QY26_PICPA
A4QPW8_MAGGR
Mycgr3108094_MYCGR
Q9UTR7_SCHPO
HCAG_07480T0_AJECA
Q2UDH3_ASPOR_ASPOR
GECA04s06742g_GEOCN
Pc12g13920.t1_PENCH
BC1T_06829_BOTFU
Q7SHC8_NEUCR
Trire259435_TRIRE
Q6CLS5_KLULA
Q9USI6_SCHPO
Hanpo2_85593_PICAN
CLUG_02567.1_CLALS
A4RNE9_MAGGR
GECA07s02551g_GEOCN
O42657_SCHPO
C4QXG1_PICPA
Q6BY65_DEBHA
CLUG_04413.1_CLALS
Q6CSX0_KLULA
Q06704_YEAST
ZYRO0B00484g_ZYGRO
CAGL0E03454g_CANGA
KLTH0H09438g_LACTH
C4R7F7_PICPA
CAGL0A02596g_CANGA
Q2UA08_ASPOR_ASPOR
CLUG_03980.1_CLALS
YALI0F30855g_YARLI
GECA03s08106g_GEOCN
BC1T_10650_BOTFU
CLUG_05241.1_CLALS
YALI0B21384g_YARLI
C4R6U0_PICPA
Q8WZY2_NEUCR
GECA07s05389g_GEOCN
O94488_SCHPO
A4R8E0_MAGGR
Q4PBB0_USTMA
4.31

Fig S8i

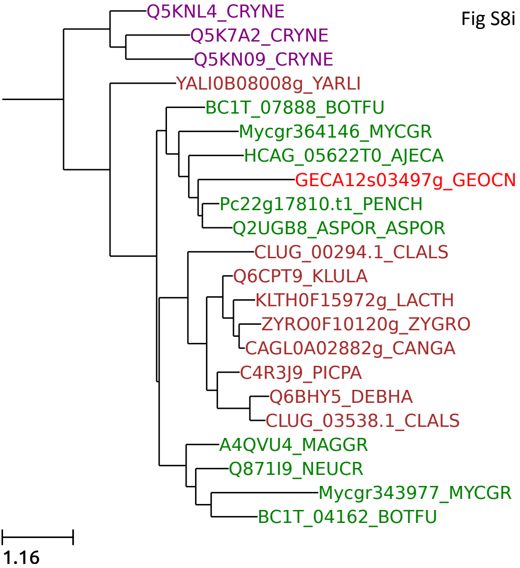
Q5KNL4_CRYNE
Q5K7A2_CRYNE
Q5KN09_CRYNE
YALI0B08008g_YARLI
BC1T_07888_BOTFU
Mycgr364146_MYCGR
HCAG_05622T0_AJECA
GECA12s03497g_GEOCN
Pc22g17810.t1_PENCH
Q2UGB8_ASPOR_ASPOR
CLUG_00294.1_CLALS
Q6CPT9_KLULA
KLTH0F15972g_LACTH
ZYRO0F10120g_ZYGRO
CAGL0A02882g_CANGA
C4R3J9_PICPA
Q6BHY5_DEBHA
CLUG_03538.1_CLALS
A4QVU4_MAGGR
Q871I9_NEUCR
Mycgr343977_MYCGR
BC1T_04162_BOTFU
1.16

Fig S8j

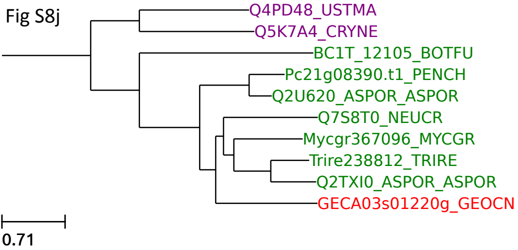
Q4PD48_USTMA
Q5K7A4_CRYNE
BC1T_12105_BOTFU
Pc21g08390.t1_PENCH
Q2U620_ASPOR_ASPOR
Q7S8T0_NEUCR
Mycgr367096_MYCGR
Trire238812_TRIRE
Q2TXI0_ASPOR_ASPOR
GECA03s01220g_GEOCN
0.71

Fig S8k

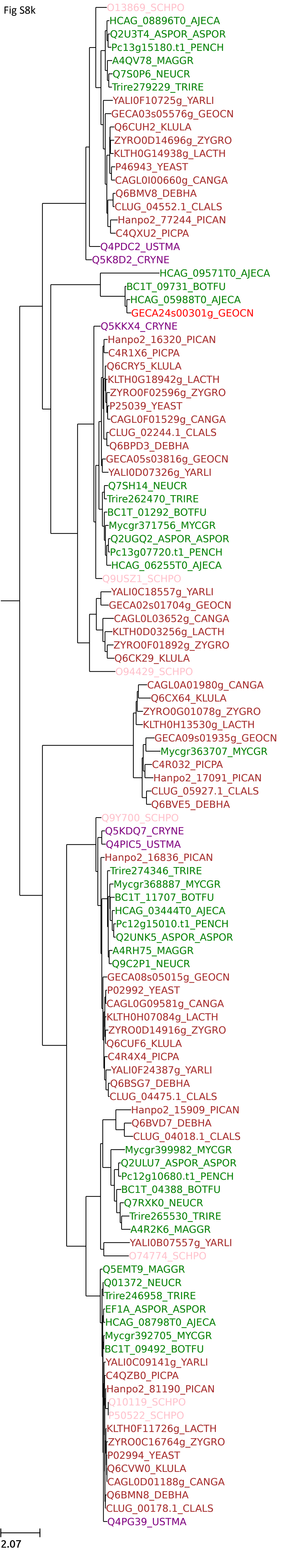

O13869_SCHPO
HCAG_08896T0_AJECA
Q2U3T4_ASPOR_ASPOR
Pc13g15180.t1_PENCH
A4QV78_MAGGR
Q7S0P6_NEUCR
Trire279229_TRIRE
YALI0F10725g_YARLI
GECA03s05576g_GEOCN
Q6CUH2_KLULA
ZYRO0D14696g_ZYGRO
KLTH0G14938g_LACTH
P46943_YEAST
CAGL0I00660g_CANGA
Q6BMV8_DEBHA
CLUG_04552.1_CLALS
Hanpo2_77244_PICAN
C4QXU2_PICPA
Q4PDC2_USTMA
Q5K8D2_CRYNE
HCAG_09571T0_AJECA
BC1T_09731_BOTFU
HCAG_05988T0_AJECA
GECA24s00301g_GEOCN
Q5KKX4_CRYNE
Hanpo2_16320_PICAN
C4R1X6_PICPA
Q6CRY5_KLULA
KLTH0G18942g_LACTH
ZYRO0F02596g_ZYGRO
P25039_YEAST
CAGL0F01529g_CANGA
CLUG_02244.1_CLALS
Q6BPD3_DEBHA
GECA05s03816g_GEOCN
YALI0D07326g_YARLI
Q7SH14_NEUCR
Trire262470_TRIRE
BC1T_01292_BOTFU
Mycgr371756_MYCGR
Q2UGQ2_ASPOR_ASPOR
Pc13g07720.t1_PENCH
HCAG_06255T0_AJECA
Q9USZ1_SCHPO
YALI0C18557g_YARLI
GECA02s01704g_GEOCN
CAGL0L03652g_CANGA
KLTH0D03256g_LACTH
ZYRO0F01892g_ZYGRO
Q6CK29_KLULA
O94429_SCHPO
CAGL0A01980g_CANGA
Q6CX64_KLULA
ZYRO0G01078g_ZYGRO
KLTH0H13530g_LACTH
GECA09s01935g_GEOCN
Mycgr363707_MYCGR
C4R032_PICPA
Hanpo2_17091_PICAN
CLUG_05927.1_CLALS
Q6BVE5_DEBHA
Q9Y700_SCHPO
Q5KDQ7_CRYNE
Q4PIC5_USTMA
Hanpo2_16836_PICAN
Trire274346_TRIRE
Mycgr368887_MYCGR
BC1T_11707_BOTFU
HCAG_03444T0_AJECA
Pc12g15010.t1_PENCH
Q2UNK5_ASPOR_ASPOR
A4R75_MAGGR
Q9C2P1_NEUCR
GECA08s05015g_GEOCN
P02992_YEAST
CAGL0G09581g_CANGA
KLTH0H07084g_LACTH
ZYRO0D14916g_ZYGRO
Q6CUF6_KLULA
C4R4X4_PICPA
YALI0F24387g_YARLI
Q6BSG7_DEBHA
CLUG_04475.1_CLALS
Hanpo2_15909_PICAN
Q6BVD7_DEBHA
CLUG_04018.1_CLALS
Mycgr399982_MYCGR
Q2ULU7_ASPOR_ASPOR
Pc12g10680.t1_PENCH
BC1T_04388_BOTFU
Q7RXK0_NEUCR
Trire265530_TRIRE
A4R2K6_MAGGR
YALI0B07557g_YARLI
O74774_SCHPO
Q5EMT9_MAGGR
Q01372_NEUCR
Trire246958_TRIRE
EF1A_ASPOR_ASPOR
HCAG_08798T0_AJECA
Mycgr392705_MYCGR
BC1T_09492_BOTFU
YALI0C09141g_YARLI
C4QZB0_PICPA
Hanpo2_81190_PICAN
Q10119_SCHPO
P50522_SCHPO
KLTH0F11726g_LACTH
ZYRO0C16764g_ZYGRO
P02994_YEAST
Q6CVW0_KLULA
CAGL0D01188g_CANGA
Q6BMN8_DEBHA
CLUG_00178.1_CLALS
Q4PG39_USTMA
2.07

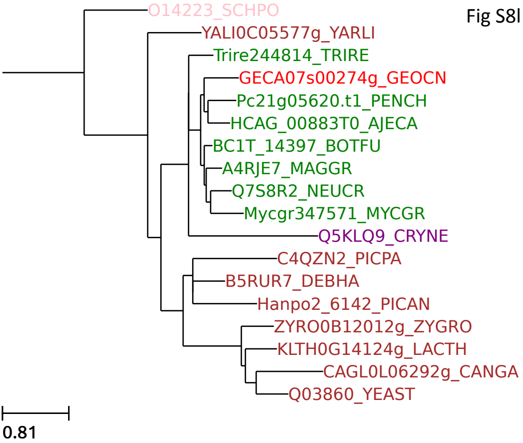
Fig S8l
O14223_SCHPO
YALI0C05577g_YARLI
Trire244814_TRIRE
GECA07s00274g_GEOCN
Pc21g05620.t1_PENCH
HCAG_00883T0_AJECA
BC1T_14397_BOTFU
A4RJE7_MAGGR
Q7S8R2_NEUCR
Mycgr347571_MYCGR
Q5KLQ9_CRYNE
C4QZN2_PICPA
B5RUR7_DEBHA
Hanpo2_6142_PICAN
ZYRO0B12012g_ZYGRO
KLTH0G14124g_LACTH
CAGL0L06292g_CANGA
Q03860_YEAST
0.81

Fig S8m

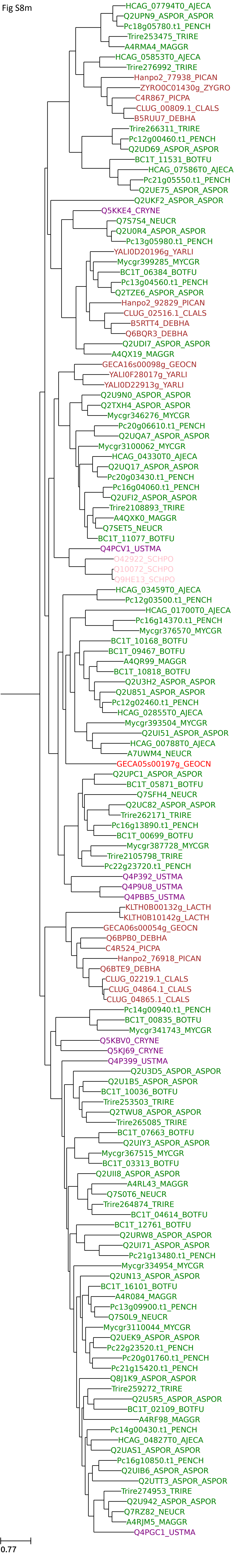
HCAG_07794T0_AJECA
Q2UPN9_ASPOR_ASPOR
Pc18g05780.t1_PENCH
Trire253475_TRIRE
A4RMA4_MAGGR
HCAG_05853T0_AJECA
Trire276992_TRIRE
Hanpo2_77938_PICAN
ZYRO0C01430g_ZYGRO
C4R867_PICPA
CLUG_00809.1_CLALS
B5RUU7_DEBHA
Trire266311_TRIRE
Pc12g00460.t1_PENCH
Q2UD69_ASPOR_ASPOR
BC1T_11531_BOTFU
HCAG_07586T0_AJECA
Pc21g05550.t1_PENCH
Q2UE75_ASPOR_ASPOR
Q2UKF2_ASPOR_ASPOR
Q5KKE4_CRYNE
Q7S7S4_NEUCR
Q2U0R4_ASPOR_ASPOR
Pc13g05980.t1_PENCH
YALI0D20196g_YARLI
Mycgr399285_MYCGR
BC1T_06384_BOTFU
Pc13g04560.t1_PENCH
Q2TZE6_ASPOR_ASPOR
Hanpo2_92829_PICAN
CLUG_02516.1_CLALS
B5RTT4_DEBHA
Q6BQR3_DEBHA
Q2UDI7_ASPOR_ASPOR
A4QX19_MAGGR
GECA16s00098g_GEOCN
YALI0F28017g_YARLI
YALI0D22913g_YARLI
Q2U9N0_ASPOR_ASPOR
Q2TXH4_ASPOR_ASPOR
Mycgr346276_MYCGR
Pc20g06610.t1_PENCH
Q2UQA7_ASPOR_ASPOR
Mycgr3100062_MYCGR
HCAG_04330T0_AJECA
Q2UQ17_ASPOR_ASPOR
Pc20g03430.t1_PENCH
Pc16g04060.t1_PENCH
Q2UFI2_ASPOR_ASPOR
Trire2108893_TRIRE
A4QXK0_MAGGR
Q7SET5_NEUCR
BC1T_11077_BOTFU
Q4PCV1_USTMA
O42922_SCHPO
Q10072_SCHPO
Q9HE13_SCHPO
HCAG_03459T0_AJECA
Pc12g03500.t1_PENCH
HCAG_01700T0_AJECA
Pc16g14370.t1_PENCH
Mycgr376570_MYCGR
BC1T_10168_BOTFU
BC1T_09467_BOTFU
A4QR99_MAGGR
BC1T_10818_BOTFU
Q2U3H2_ASPOR_ASPOR
Q2U851_ASPOR_ASPOR
Pc12g02460.t1_PENCH
HCAG_02855T0_AJECA
Mycgr393504_MYCGR
Q2UI51_ASPOR_ASPOR
HCAG_00788T0_AJECA
A7UWM4_NEUCR
GECA05s00197g_GEOCN
Q2UPC1_ASPOR_ASPOR
BC1T_05871_BOTFU
Q7SFH4_NEUCR
Q2UC82_ASPOR_ASPOR
Trire262171_TRIRE
Pc16g13890.t1_PENCH
BC1T_00699_BOTFU
Mycgr387728_MYCGR
Trire2105798_TRIRE
Pc22g23720.t1_PENCH
Q4P392_USTMA
Q4P9U8_USTMA
Q4PBB5_USTMA
KLTH0B00132g_LACTH
KLTH0B10142g_LACTH
GECA06s00054g_GEOCN
Q6BPB0_DEBHA
C4R524_PICPA
Hanpo2_76918_PICAN
Q6BTE9_DEBHA
CLUG_02219.1_CLALS
CLUG_04864.1_CLALS
CLUG_04865.1_CLALS
Pc14g00940.t1_PENCH
BC1T_00835_BOTFU
Mycgr341743_MYCGR
Q5KBV0_CRYNE
Q5KJ69_CRYNE
Q4P399_USTMA
Q2U3D5_ASPOR_ASPOR
Q2U1B5_ASPOR_ASPOR
BC1T_10036_BOTFU
Trire253503_TRIRE
Q2TWU8_ASPOR_ASPOR
Trire265085_TRIRE
BC1T_07663_BOTFU
Q2UIY3_ASPOR_ASPOR
Mycgr367515_MYCGR
BC1T_03313_BOTFU
Q2UII8_ASPOR_ASPOR
A4RL43_MAGGR
Q7S0T6_NEUCR
Trire264874_TRIRE
BC1T_04614_BOTFU
BC1T_12761_BOTFU
Q2URW8_ASPOR_ASPOR
Q2UI71_ASPOR_ASPOR
Pc21g13480.t1_PENCH
Mycgr334954_MYCGR
Q2UN13_ASPOR_ASPOR
BC1T_16101_BOTFU
A4R084_MAGGR
Pc13g09900.t1_PENCH
Q7S0L9_NEUCR
Mycgr3110044_MYCGR
Q2UEK9_ASPOR_ASPOR
Pc22g23520.t1_PENCH
Pc20g01760.t1_PENCH
Pc21g15420.t1_PENCH
Q8J1K9_ASPOR_ASPOR
Trire259272_TRIRE
Q2U5R5_ASPOR_ASPOR
BC1T_02109_BOTFU
A4RF98_MAGGR
Pc14g00430.t1_PENCH
HCAG_04827T0_AJECA
Q2UAS1_ASPOR_ASPOR
Pc16g10850.t1_PENCH
Q2UIB6_ASPOR_ASPOR
Q2UTT3_ASPOR_ASPOR
Trire274953_TRIRE
Q2U942_ASPOR_ASPOR
Q7RZ82_NEUCR
A4RJM5_MAGGR
Q4PGC1_USTMA
0.77

Fig S8n

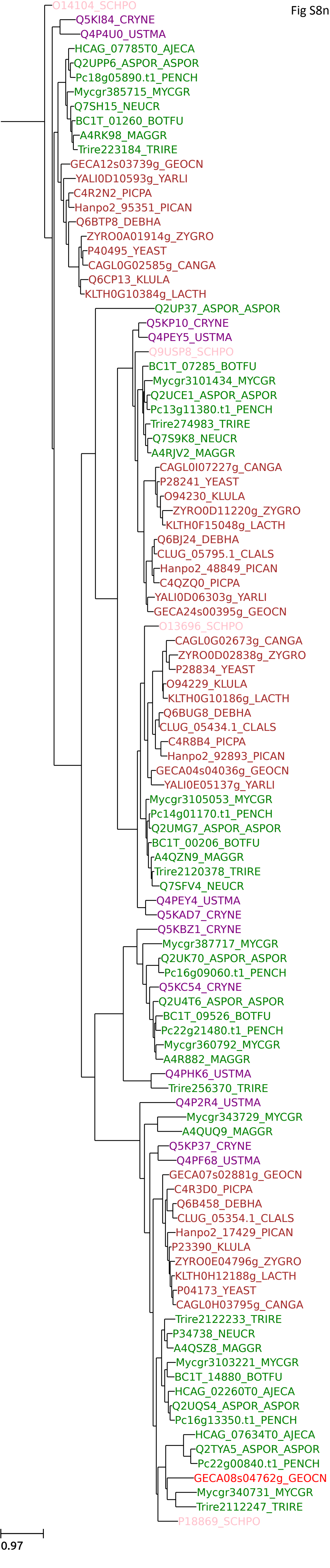
O14104_SCHPO
Q5KI84_CRYNE
Q4P4U0_USTMA
HCAG_07785T0_AJECA
Q2UPP6_ASPOR_ASPOR
Pc18g05890.t1_PENCH
Mycgr385715_MYCGR
Q7SH15_NEUCR
BC1T_01260_BOTFU
A4RK98_MAGGR
Trire223184_TRIRE
GECA12s03739g_GEOCN
YALI0D10593g_YARLI
C4R2N2_PICPA
Hanpo2_95351_PICAN
Q6BTP8_DEBHA
ZYRO0A01914g_ZYGRO
P40495_YEAST
CAGL0G02585g_CANGA
Q6CP13_KLULA
KLTH0G10384g_LACTH
Q2UP37_ASPOR_ASPOR
Q5KP10_CRYNE
Q4PEY5_USTMA
Q9USP8_SCHPO
BC1T_07285_BOTFU
Mycgr3101434_MYCGR
Q2UCE1_ASPOR_ASPOR
Pc13g11380.t1_PENCH
Trire274983_TRIRE
Q7S9K8_NEUCR
A4RJV2_MAGGR
CAGL0I07227g_CANGA
P28241_YEAST
O94230_KLULA
ZYRO0D11220g_ZYGRO
KLTH0F15048g_LACTH
Q6BJ24_DEBHA
CLUG_05795.1_CLALS
Hanpo2_48849_PICAN
C4QZQ0_PICPA
YALI0D06303g_YARLI
GECA24s00395g_GEOCN
O13696_SCHPO
CAGL0G02673g_CANGA
ZYRO0D02838g_ZYGRO
P28834_YEAST
O94229_KLULA
KLTH0G10186g_LACTH
Q6BUG8_DEBHA
CLUG_05434.1_CLALS
C4R8B4_PICPA
Hanpo2_92893_PICAN
GECA04s04036g_GEOCN
YALI0E05137g_YARLI
Mycgr3105053_MYCGR
Pc14g01170.t1_PENCH
Q2UMG7_ASPOR_ASPOR
BC1T_00206_BOTFU
A4QZN9_MAGGR
Trire2120378_TRIRE
Q7SFV4_NEUCR
Q4PEY4_USTMA
Q5KAD7_CRYNE
Q5KBZ1_CRYNE
Mycgr387717_MYCGR
Q2UK70_ASPOR_ASPOR
Pc16g09060.t1_PENCH
Q5KC54_CRYNE
Q2U4T6_ASPOR_ASPOR
BC1T_09526_BOTFU
Pc22g21480.t1_PENCH
Mycgr360792_MYCGR
A4R882_MAGGR
Q4PHK6_USTMA
Trire256370_TRIRE
Q4P2R4_USTMA
Mycgr343729_MYCGR
A4QUQ9_MAGGR
Q5KP37_CRYNE
Q4PF68_USTMA
GECA07s02881g_GEOCN
C4R3D0_PICPA
Q6B458_DEBHA
CLUG_05354.1_CLALS
Hanpo2_17429_PICAN
P23390_KLULA
ZYRO0E04796g_ZYGRO
KLTH0H12188g_LACTH
P04173_YEAST
CAGL0H03795g_CANGA
Trire2122233_TRIRE
P34738_NEUCR
A4QSZ8_MAGGR
Mycgr3103221_MYCGR
BC1T_14880_BOTFU
HCAG_02260T0_AJECA
Q2UQS4_ASPOR_ASPOR
Pc16g13350.t1_PENCH
HCAG_07634T0_AJECA
Q2TYA5_ASPOR_ASPOR
Pc22g00840.t1_PENCH
GECA08s04762g_GEOCN
Mycgr340731_MYCGR
Trire2112247_TRIRE
P18869_SCHPO
0.97

Fig S8o

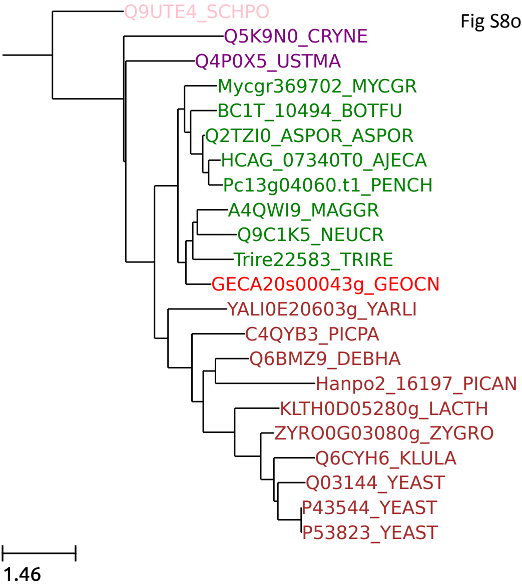
Q9UTE4_SCHPO
Q5K9N0_CRYNE
Q4P0X5_USTMA
Mycgr369702_MYCGR
BC1T_10494_BOTFU
Q2TZI0_ASPOR_ASPOR
HCAG_07340T0_AJECA
Pc13g04060.t1_PENCH
A4QWI9_MAGGR
Q9C1K5_NEUCR
Trire22583_TRIRE
GECA20s00043g_GEOCN
YALI0E20603g_YARLI
C4QYB3_PICPA
Q6BMZ9_DEBHA
Hanpo2_16197_PICAN
KLTH0D05280g_LACTH
ZYRO0G03080g_ZYGRO
Q6CYH6_KLULA
Q03144_YEAST
P43544_YEAST
P53823_YEAST
1.46

Fig S8p

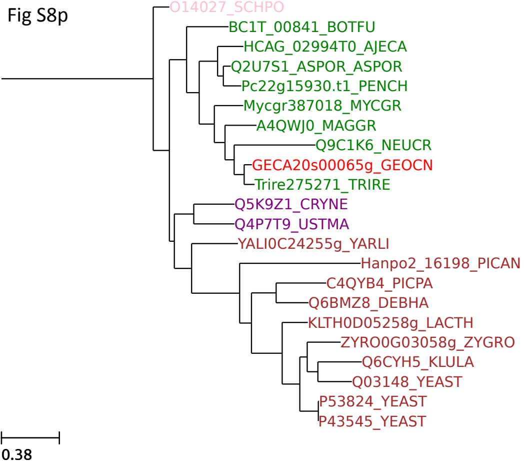

O14027_SCHPO
BC1T_00841_BOTFU
HCAG_02994T0_AJECA
Q2U7S1_ASPOR_ASPOR
Pc22g15930.t1_PENCH
Mycgr387018_MYCGR
A4QWJ0_MAGGR
Q9C1K6_NEUCR
GECA20s00065g_GEOCN
Trire275271_TRIRE
Q5K9Z1_CRYNE
Q4P7T9_USTMA
YALI0C24255g_YARLI
Hanpo2_16198_PICAN
C4QYB4_PICPA
Q6BMZ8_DEBHA
KLTH0D05258g_LACTH
ZYRO0G03058g_ZYGRO
Q6CYH5_KLULA
Q03148_YEAST
P53824_YEAST
P43545_YEAST
0.38

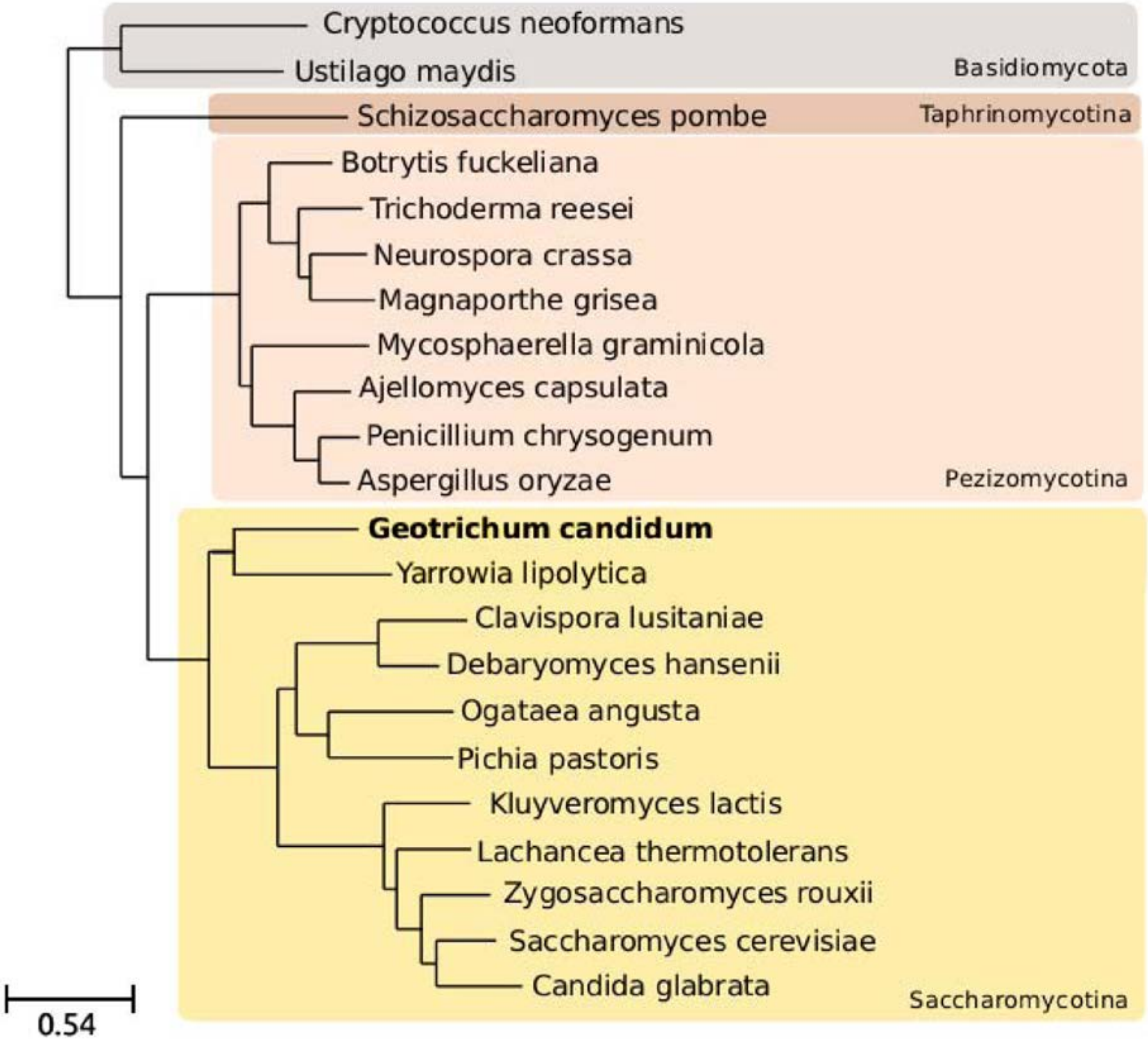

Supplementary Figure S9. **Species tree reconstruction from the *G. candidum* phylome**. The tree species was reconstructed using the RAxML program as described in Materials and Methods. Proteins with a one-to-one orthology relationship to all the considered species were selected from the *G. candidum* phylome. A total of 302 protein alignments were concatenated into a multiple sequence alignment. The final alignment contained 170,787 amino acids. The bar indicates the scale of branch length.

Differential gene retention as an evolutionary mechanism to generate biodiversity in yeasts

Guillaume Morel, Lieven Sterck, Dominique Swennen, Marina Marcet-Houben, Djamila Onesime, Anthony Levasseur, Noémie Jacques, Sandrine Mallet, Arnaux Couloux., Karine Labadie, Joëlle Amselem, Jean-Marie Beckerich, Bernard Henrissat, Yves Van de Peer, Patrick Wincker, Jean-Luc Souciet, Toni Gabaldón, Colin R. Tinsley & Serge Casaregola

Supplementary tables

Supplementary Table 1. **Genome overview**

Scaffold	Size (bp)	Number of gaps	Total gap size (bp)	Number of protein coding genes (*)	Number of pseudogenes	Number of tRNA genes
scaffold 01	2,581,591	134	209,110 (8.10%)	668	13	36
scaffold 02	2,106,585	124	178,429 (8.47%)	558	6	20
scaffold 03	1,796,091	87	143,169 (7.97%)	492	4	33
scaffold 04	1,686,461	74	110,146 (6.53%)	498	5	18
scaffold 05	1,653,926	83	92,238 (5.57%)	474	1	16
scaffold 06	1,264,337	60	108,269 (8.56%)	338	0	15
scaffold 07	1,211,666	64	104,510 (8.62%)	336	2	22
scaffold 08	1,159,651	53	91,539 (7.89%)	309	5	15
scaffold 09	1,063,388	50	73,588 (6.92%)	259	4	11
scaffold 10	918,757	61	121,292 (13.20%)	234	2	22
scaffold 11	909,630	50	76,909 (8.45%)	262	2	9
scaffold 12	890,415	43	56,095 (6.29%)	270	2	5
scaffold 13	871,522	38	59,148 (6.78%)	230	5	5
scaffold 14	795,198	46	74,252 (9.33%)	208	2	12
scaffold 15	711,411	28	49,387 (6.94%)	229	2	13
scaffold 16	665,493	32	49,807 (7.48%)	186	3	12
scaffold 17	608,101	36	57,666 (9.48%)	163	2	7
scaffold 18	562,025	20	41,863 (7.44%)	168	3	5
scaffold 19	507,124	22	38,528 (7.59%)	126	2	12
scaffold 20	368,852	14	19,981 (5.41%)	107	1	4
scaffold 21	313,304	13	17,857 (5.69%)	91	2	5
scaffold 22	313,014	13	17,044 (5.44%)	88	4	1
scaffold 23	307,402	13	22,984 (7.47%)	81	1	6
scaffold 24	271,541	13	16,510 (6.08%)	101	2	8
scaffold 25	237,622	17	22,898 (9.63%)	79	0	3
scaffold 26	220,840	10	15,595 (7.06%)	59	1	2
scaffold 27	219,481	12	13,837 (6.30%)	53	2	7
scaffold32	620,629	206	61,001 (9.82%)	137	2	25
**Total**	**24,836,057**	**1416**	**1,929,829 (7.77%)**	**6804**	**80**	**349**

(*) excluding pseudogenes and transposons

Supplementary Table 2. **List of *G. candidum* genes encoding NAD dehydrogenase complex I proteins and their corresponding orthologs in *Y. lipolytica***

**Complex I proteins**	***Geotrichum candidum***	***Yarrowia lipolytica***
NUAM	GECA17s02276g/GECA08s04113g	YALI0D05467g
NUBM	GECA04s04729g	YALI0B20372g
NUCM	GECA09s04641g	YALI0F17248g
NUGM	GECA02s07666g	YALI0F02123g
NUHM	GECA02s06929g	YALI0D00737g
NUIM	GECA10s03684g	YALI0F00924g
NUKM	GECA01s00098g	YALI0F06050g
NUEM	GECA12s01440g	YALI0D24585g
ST1	GECA04s07567g	YALI0F23551g
NESM	GECA04s01517g	YALI0E29095g
NUJM	GECA01s05532g	YALI0E11891g
NUZM	GECA18s02386g	YALI0A02651g
NUPM	GECA05s05884g	YALI0A20680g
NUXM	GECA10s02782g	YALI0E28424g
N7BM	GECA02s04916g	YALI0B00792g
NUYM	GECA07s00098g	YALI0B14861g
NUFM	GECA03s07314g	YALI0E23089g
NIAM	GECA03s00571g	YALI0D04939g
NB4M	GECA13s02562g	YALI0A01419g
NUMM	GECA04s05059g	YALI0D19030g
NB6M	GECA01s02859g	YALI0E28930g
NUNM	No hit	YALI0F14003g
NI2M	GECA09s00604g	YALI0D07216g
NB8M	GECA11s01715g	YALI0E31766g
NIDM	GECA05s00164g	YALI0A17946g
NB5M	GECA07s00719g	YALI0F06061g
ACPM1	GECA03s01275g	YALI0D24629g
ACPM2	GECA07s04509g	YALI0D14850g
NIPM	GECA20s01044g	YALI0F18359g
NIMM	GECA08s03189g	YALI0C21789g
NI8M	GECA02s07908g/GECA19s00890g	YALI0C03201g
NI9M	GECA06s05081g	YALI0E23749g
NB2M	GECA11s01638g	YALI0D10274g

The *Y. lipolytica* data are from Morgner *et al*, 2008 and Dröse *et al*, 2011.

Supplementary Table 3**. Synteny blocks**

	Number of syntenic blocks		
**Nb of genes per syntenic block**	**ARAD/YALI**	**ARAD/GECA**	**YALI/GECA**
2	424	390	328
3	199	241	134
4	104	79	31
5	38	44	12
6	20	16	6
7	8	3	0
8	1	4	0
9	2	1	0
10	0	0	0
11	1	0	0
**Total nb of synthenic blocks**	797	778	511
**Nb of genes in synthenic blocks**	2264	2197	1278

Supplementary Table 4**. Distribution of spliceosomal introns per *G. candidum* protein coding gene.**

**Genome content (excluding transposons)**	**Nb introns/gene**	**Nb genes**
Number of protein coding genes		6804
Total number of spliceosomal introns		3830
Number of genes with	0	2414
	1	1526
	2	549
	3	227
	4	68
	5	27
	6	8
	7	5
	8	2
	9	1
	10	1

Supplementary Table 5. **Chitin metabolism genes in *G. candidum*.**

**Gene name in *S. cerevisiae***	**Function**	**Copy number in *S. cerevisiae***	**Copy number in *G. candidum***	**Copy number in *N. crassa***	**Copy number in *A. oryzae***
CTS1	endochitinase	1	4	2	2
CTS2	chitinase	1	2	3	2
CHS1	chitin synthase I	1	2	1	1
CHS2	chitin synthase II	1	2	1	1
CHS3	chitin synthase III	1	6	4	3
CHS7	chitin biosynthesis through the regulation of chitin synthase III	1	2	1	1
np	filamentous fungal-type chitin synthase	np	2*	1	3
CYK3	chitin synthase activator	1	2	1	1
SKT5	chitin synthase III activator	1	5 (+ 1 pseudogene)	1	2
CDA1	chitin deacetylase	1	2	1	0
CRH1	chitin transglycosylase	1	2	2	2
GFA1	glutamine-fructose-6-phosphate amidotransferases	1	2	1	2
UTR2	chitin transglycosylase	1	2	1	1

np: not present

*GECA04s06797g and GECA14s02386g

Supplementary Table 6. **Lipases and β-oxydation genes in *G. candidum***

	***S. cerevisiae* gene name**	***G. candidum* genes with orthologs in Saccharomycotina**	***G. candidum* genes with orthologs in Pezizomycotina**	**Annotation**
**Lipase genes**				
	-		GECA03s04685g*	Lipase: extracellular triglyceride lipase
	-		GECA03s04652g	//
	-		GECA03s04718g	//
	-		GECA03s04212g	//
**B-oxidation genes**				
	POX1	GECA14s03310g		Fatty-acyl coenzyme A oxidase
	CTA1	GECA27s00395g	GECA01s02936g	Catalase A
	POT1	GECA17s00318g		3-ketoacyl-CoA thiolase with broad chain length specificity
	FOX2	GECA16s01759g		3-hydroxyacyl-CoA dehydrogenase and enoyl-CoA hydratase
	FOX3	GECA02s07259g		3-ketoacyl-CoA thiolase with broad chain length specificity
	//	GECA11s01451g		//
	ECI1	GECA07s05048g		Peroxisomal delta3,delta2-enoyl-CoA isomerase
	SPS19	GECA07s03772g		Peroxisomal 2,4-dienoyl-CoA reductase

*Similar lipases were found in *Candida rugosa*, *Candida cylindracea* and an undescribed species *Candida* sp. ACITM

Supplementary Table 7. **List of putative *G. candidum* genes encoding proteins involved in intron splicing**.

	Saccharomycotina*	Pezizomycotina**	Pezizomycotina and *Y. lipolytica****
**snRNP proteins**			
Core Sm proteins			
SMB1	GECA10s03244g	GECA02s09393g	
SMD1	GECA01s11065g		
SMD2	GECA08s04608g		
SMD3	GECA19s01594g		
SME1	GECA03s06555g		
SMX2	GECA03s04850g		
SMX3	GECA08s03530g		
Lsm proteins			
LSM1	GECA13s00054g		
LSM2	GECA02s09470g		
LSM3	GECA08s03838g		
LSM4	GECA11s03486g		
LSM5	-	-	GECA03s02815g
LSM6	GECA01s06225g		
LSM7	GECA05s02749g		
LSM8	GECA05s00901g		
U1			
SNP1	GECA16s02694g		
MUD1	GECA17s02001g		
YHC1	GECA15s02848g		
LUC7	GECA08s03079g		
NAM8	GECA15s02716g		
PRP39	GECA06s05158g		
PRP40	GECA05s06247g		
PRP42	GECA26s00285g		
SNU56	No hit		
SNU71	GECA02s02903g		
U2			
LEA1	GECA03s04113g		
MSL1	GECA17s02001g		
//	GECA02s04234g		
CUS2	GECA05s00626g		
SF3a			
PRP9	GECA10s03420g		
PRP11	GECA06s00087g		
PRP21	GECA06s02518g		
SF3b			
CUS1	GECA01s06764g		
RSE1	GECA14s00109g		

HSH49	GECA03s03684g
HSH155	GECA07s00175g
RDS3	GECA02s01990g
YSF3	GECA18s02463g
RES	
IST3	GECA15s01913g
BUD13	GECA05s07358g
PML1	GECA11s03662g
U4	
PRP3	GECA23s00538g
PRP4	GECA08s03464g
SNU13	GECA14s01429g
**U5**	
DIB1	GECA02s09492g
PRP8	GECA07s01220g
PRP28	GECA05s02265g
BRR2	GECA23s00197g
LIN1	GECA02s08832g
SNU114	GECA07s02419g
PRP6	GECA03s04201g
AAR2	GECA08s05169g
**U6**	
PRP24	GECA01s06819g
**U4/U6**	
PRP3	GECA23s00538g
PRP4	GECA08s03464g
SNU13	GECA14s01429g
PRP31	GECA23s00802g
**U4/U6/U5**	
PRP3	GECA23s00538g
PRP4	GECA08s03464g
SNU13	GECA14s01429g
PRP31	GECA23s00802g
DIB1	GECA02s09492g
PRP8	GECA07s01220g
PRP28	GECA05s02265g
BRR2	GECA23s00197g
SNU114	GECA07s02419g
PRP6	GECA03s04201g
PRP38	No hit
SNU23	GECA12s01682g
SNU66	GECA01s06786g
SPP381	No hit
SAD1	GECA05s02463g

Non-snRNP proteins

Assembly

MSL5		GECA07s00318g
MUD2	GECA01s01143g	

SUB2	GECA19s00604g		
PRP5	GECA06s04410g		
Activation			
PRP19	GECA01s10471g		
SNT309	No hit		
CEF1	GECA10s03651g		
SYF1	GECA02s02914g		
CLF1	GECA20s01055g		
SYF2	GECA05s03035g		
ISY1	GECA02s06742g		
NTC20	No hit		
CWC2	GECA05s02254g		
PRP45	GECA11s00032g		
PRP46	GECA16s01440g		
ECM2	GECA18s00736g		
CWC21	GECA03s00285g		
First reaction			
CWC22	GECA09s01143g		
SPP2	No hit		
PRP2	GECA17s02375g		
	GECA32s02001g		
CWC25	GECA05s02705g		
YJU2	GECA08s04630g	GECA15s01110g	
Second reaction			
PRP17	GECA07s00538g		
PRP16	No hit		
SLU7	GECA05s02892g		
PRP18	GECA05s02848g		
PRP22	GECA18s00395g		
Disassembly			
SPP382/NTR1	GECA17s00054g		
NTR2	GECA03s07039g		
PRP43	GECA20s01264g	GECA16s02188g	
Debranching enzyme			
DBR1	GECA04s02529g		
Unknown			
BUD31	GECA07s03739g		
CWC15	-	-	GECA04s02056g
CWC24	GECA01s05400g		
CWC27	GECA26s00703g		
URN1	GECA05s06346g		

* genes which can also be found in other Saccharomycotina
**genes with Pezizomycotina orthologs only
***genes with Pezizomycotina and *Y. lipolytica* orthologs

Supplementary Table 8. **Comparison of the distribution of the homologous genes involved in hyphal growth in *Neurospora crassa*, *G. candidum* and *Y. lipolytica***

***Neurospora crassa* functions and nomenclature**	***Neurospora crassa***	***Geotrichum candidum***	***Yarrowia lipolytica***
**Cell wall**			
Glucan synthase regulator gs-1	NCU04189	GECA01s04894g	YALI0B20570g
Chitin synthase chs-1, chs-2, chs-3, chs-4, chs-5, chs-6, chs-7	NCU03611<br>NCU05239<br>NCU04251<br>NCU09324<br>NCU04352<br>NCU05268<br>NCU05350	GECA04s06797g<br>GECA14s02386g<br>GECA09s01715g<br>GECA05s00582g<br>GECA06s02375g<br>GECA01s05081g<br>GECA01s06676g<br>GECA01s06687g<br>GECA05s05191g<br>GECA06s01830g<br>GECA09s02287g<br>GECA20s01385g<br>GECA11s03596g<br>GECA13s00978g<br>GECA32s00472g	YALI0D03179g<br>YALI0B16324g<br>YALI0D25938g<br>YALI0C24354g<br>YALI0D17006g<br>YALI0E10417g<br>YALI0E22198g<br>YALI0F28655g<br>YALI0E16170g
**Cytoskeleton genes**			
Beta-tubulin bml	NCU04054	GECA32s02265g<br>GECA04s07127g	YALI0E00726g<br>YALI0E26961g
Fimbrin, an actin-binding protein fim	NCU003992	GECA02s02650g	YALI0B17622g
Tropomyosin, an actin-binding protein tpm-1	NCU001204	GECA18s02606g<br>GECA01s02771g	YALI0F27049g
Subunit of the Arp2/3 complex arp-3	NCU001756	GECA12s01121g	YALI0E34170g
**Nuclei**			
NDR protein kinase dbf-2	NCU09071	GECA05s06973g	YALI0B14201g
Histone hh-1	NCU06863	GECA04s00890g	YALI0B16280g
Heterochromatin protein HP1 Hpo	NCU04018		
Nucleoporin son-1	NCU04288	GECA16s01198g	YALI0E23265g
**Endoplasmic reticulum**			
ER associated HSP grp-78	NCU03982	GECA07s00527g	YALI0E13706g
Dolichol-phosphate mannosyltransferase dpm	NCU07965	GECA14s02067g	YALI0D06281g
**Vacuole**			
Subunit A of vacuolar ATPase vma-1	NCU01207	GECA02s07754g	YALI0A09900g
Vacuole-associated SNARE protein vma-3	NCU06777	GECA06s02727g<br>GECA01s05279g	YALI0E11825g<br>YALI0C22275g
Subunit C of vacuolar ATPase vma-5	NCU09897	GECA12s01572g	YALI0A11143g

**Mitochondria**			
Mitochondrial acetylornithine-glutamate transacetylase. Arginine biosynthesis arg-4	NCU10468	GECA02s00604g	YALI0E13057g
**Golgi**			
Component of Golgi body-associated retrograde protein complex vps-52	NCU05273	GECA01s04157g	YALI0F07381g
**Calcium transporters**			
Ca2+/H+-ATPase nca-1, nca-2, nca-3	NCU03305<br>NCU04736<br>NCU05154	GECA06s05246g<br>GECA09s01066g<br>GECA03s01000g	YALI0E09471g<br>YALI0D04873g
Ca2+/H+ exchange protein cax	NCU07075	GECA06s00263g<br>GECA12s00120g	YALI0B09273g
**Exocyst**			
Exocyst complex sec-3, sec-5, sec-6, sec-8, sec-15, exo-70	NCU09869<br>NCU07698<br>NCU03341<br>NCU04190<br>NCU00117<br>NCU08012<br>NCU06631	GECA17s01242g<br>GECA06s03123g<br>GECA25s00681g<br>GECA02s05917g<br>GECA16s00263g<br>GECA01s07512g<br>GECA06s00604g<br>GECA02s04586g	YALI0F21681g<br>YALI0E33759g<br>YALI0A19052g<br>YALI0F12969g<br>YALI0C01595g<br>YALI0D08492g<br>YALI0F11143g<br>YALI0C11946g
**Polarity**			
MAP kinase activator bem-1	NCU06593	GECA04s04883g<br>GECA21s00131g	YALI0F27643g
Involved in actin organization bni-1	NCU01431	GECA12s01407g<br>GECA04s04894g	YALI0D10879g
Putative involved in septum formation cla-4	NCU00406	GECA11s02155g<br>GECA04s06214g	YALI0C22770g<br>YALI0F00572g
NDR kinase, essential for polar cell extension cot-1	NCU07296	GECA02s00043g<br>GECA10s01297g	YALI0B04268g
RHO-1-specific GAP. Involved in coordinating apical tip growth lrg-1	NCU02689	GECA06s02760g<br>GECA03s04938g	YALI0F13211g
MAP-kinase2, required for cell fusion mak-2	NCU02393	GECA01s10548g	YALI0E23496g
NDR kinase pod-6	NCU02537	GECA13s03233g	YALI0E34001g
Subunit of the polarisome complex spa-2	NCU03115	GECA09s03684g<br>GECA05s03904g	YALI0F16665g
**Septation**			
Involved in septum formation bud-3	NCU06579	GECA18s01121g<br>GECA23s00054g	YALI0C16346g
Involved in septum formation and putative landmark protein bud-4	NCU00152	GECA03s02749g<br>GECA13s02529g	YALI0D11880g

Putatively involved in septum formation cdc-12	NCU03795	GECA22s01297g<br>GECA03s00736g	YALI0D27148g<br>YALI0F26873g
Involved in septum formation and putative landmark protein rgf-3	NCU02131	GECA15s00659g	YALI0E12155g
Rho GTPase rho-4	NCU03407	GECA03s01451g	YALI0E23001g<br>YALI0D01045g
Contributes to septal plugging so	NCU02794	GECA07s04663g	YALI0E10549g
**Hyphal fusion**			
Pheromone-regulated membrane protein 1-like, involved in cell fusion events prm-1	NCU09337	GECA09s00516g	YALI0E08580g

Supplementary Table 9. **List of genomes used in this study**

Species	Strain	Sequence source
Eremothecium gossypi	ATCC 10895^T	Ashbya Genome Database
Aspergillus fumigatus	Af293	NCBI
Aspergillus nidulans	FGSC A4	Broad
Candida glabrata	CBS 138^T	Genolevures
Candida lusitaniae	ATCC 42720	Broad
Coccidioides immitis	RS	Broad
Cryptococcus neoformans	JEC21	NCBI
Debaryomyces hansenii	CBS 767^T	Genolevures
Fusarium graminearum	PH-1	Broad
Geotrichum candium	CLIB 918 (ATCC 204307)	ORCAE
Kluyveromyces lactis	CLIB 210	Genolevures
Komagataella pastoris	CBS 7435	ORCAE
Lachancea kluyveri	CBS 3082^T	Genolevures
Lachancea thermotolerans	CBS 6340^T	Genolevures
Magnaporthe grisea	70-15	Broad
Millerozyma (sorbitophila) farinosa	CBS 7064	Genolevures
Neurospora crassa	OR74A	Broad
Ogataea parapolymorpha	CBS 4732^T	JGI
Penicillium chrysogenum	Wisconsin 54-1255	JGI
Phanerochaete chrysosporium	RP-78	JGI
Saccharomyces (uvarum) bayanus	623-6C	Broad
Saccharomyces cerevisiae	S288c	SGD
Saccharomyces paradoxus	NRRL Y-17217T	Broad
Scheffersomyces stipitis	CBS 6054^T	JGI
Schizosaccharomyces pombe	972h	NCBI
Sclerotinia sclerotiorum	1980 UF-70	Broad
Trichoderma reesei	QM6a	JGI
Ustilago maydis	521	Broad
Yarrowia lipolytica	CLIB 122	Genolevures
Zygosaccharomyces rouxii	CBS 732^T	Genolevures

The addresses of the databases are: Ashbya Genome Database, http://agd.vital-it.ch, Broad, http://www.broadinstitute.org/annotation/fungi ; Genolevures, http://www.genolevures.org; JGI, http://genome.jgi-psf.org/programs/fungi; NCBI, http://www.ncbi.nlm.nih.gov/genome ; ORCAE, http://bioinformatics.psb.ugent.be/orcae; SGD, http://yeastgenome.org.

Differential gene retention as an evolutionary mechanism to generate biodiversity in yeasts

Guillaume Morel, Lieven Sterck, Dominique Swennen, Marina Marcet-Houben, Djamila Onesime, Anthony Levasseur, Noémie Jacques, Sandrine Mallet, Arnaux Couloux., Karine Labadie, Joëlle Amselem, Jean-Marie Beckerich, Bernard Henrissat, Yves Van de Peer, Patrick Wincker, Jean-Luc Souciet, Toni Gabaldón, Colin R. Tinsley & Serge Casaregola

Supplementary note

Transposable elements

Long terminal repeat (LTR) retrotransposons and LINEs were found as remnants, in the assembly. At least five families of DNA transposons closely related to Tc elements; two families of Mutator-like elements that comprise four entire copies, one partial copy and three pseudogenes were detected. The Mutator-like elements were found to be closely related to Pezizomycotina elements, but not to the *Yarrowia lipolytica* Mutator-like Mutyl element [1]. In addition, analysis of reads using the REPET program [2] was performed; it suggested the presence of the first MITE elements (Miniature Inverted-repeat Transposable Element) to be found in a Saccharomycotina species. This 452 bp element is represented in the assembly by five full-length copies and several degenerate copies. Like other known MITEs, the *G. candidum* element is able to form a stable hairpin-like secondary structure (not shown), but it lacks the conserved terminal inverted repeats.

A solo LTR of 382 bp was also found in 66 copies in 12 of the scaffolds. The detectable copies of this repeated element were all at the ends of the scaffolds, except for internal clusters found in scaffold 3 and in scaffold 6 (11 and 8 copies, respectively). In these two scaffolds, the solo LTRs are located in regions covering over 20 kb and containing large gaps, suggesting that these regions also contain entire transposons. The presence of these clusters in the ends of 10 scaffolds very likely prevented the assembly into larger scaffolds. The presence of a number of transposable element hot-spots is reminiscent of the situation described in two yeasts, *Scheffersomyces stipitis*[3] and *D. hansenii* (Genolevures consortium, unpublished; [4]), where the Tps5 and Tdh5 LTR-retrotransposons, respectively, of each species were all found clustered in one locus per chromosome. These regions were proposed to contain the centromeres [4], as already seen in *N. crassa* [5,6]. A similar observation was recently made for the yeast *Hansenula* (*Ogataea parapolymorpha*) *polymorpha* [7]. Although pulsed-field gel electrophoresis failed to separate the chromosomes of *G. candidum* to estimate the chromosome number, a cytological analysis by [8] found eight chromosomes in this species. The number of LTR clusters that we identified (two internally and 10 at the ends of other scaffolds) fits the presence of eight chromosomes in this strain.

Various features of the *G. candidum* genome

Sequence homology using fungal sequences in the Rfam database (http://rfam.sanger.ac.uk/) detected TPP riboswitch candidates in the 5' sequence of the *THI4* and the *DUR3* homologs.

An intein of 1,237 bp was detected in GECA09s00186g encoding a subunit of the translation factor eIF2. It is a full-length intein presenting around 40% sequence similarity with VMA1-derived endonucleases from Saccharomicotina over 485 aa. In addition to the intein in the vacuolar ATPase gene VMA, inteins have previously been found in the glutamate synthase gene (GLT1) and in the threonyl-tRNA synthetase gene (ThrRS) in various Saccharomycotina species [9]. Various inteins have been found in filamentous fungi [9]. This is the first to be found in a fungal gene encoding a translation factor.

The mitochondrial DNA sequence is 28,008 bp long and has a GC content of 28%, placing it midway between those of *S. cerevisiae* (20%) and *D. hansenii* (38%) (Supplementary Figure 1). It carries 14 protein-coding genes: *COB*, *COXI*, *COXII*, *COXIII*, *ATP6*, *ATP8*, *ATP9*, VAR1, plus six ubiquinone oxidoreductase complex I protein genes *NADH1, NADH2, NDH3, NADH4, NADH5* and *NADH6*. A total of 23 tRNA genes and SSU and LSU rRNA genes were found (Supplementary Fig. 1). The ATP9 gene, the downstream tRNA-Phe gene and a tRNA-Arg gene are oriented counterclockwise. Interestingly, there is only one intron, (carrying an endonuclease, in the *COB* gene), in the mtDNA, rendering *G. candidum* the Saccharomycotina species with the lowest number of introns. *Candida phangngensis* carries two introns splitting the *COB* gene [10].

Sliceosomal introns and spliceosome

A total of 14 introns departed from the consensus GT in 5' with 12 introns starting with GC, one with GA and one with GG. In addition only one intron did not end with AG. These results were verified using RNA sequencing. A maximum of 10 introns was found in GECA13s02177g, a gene coding for an *MRE11* homolog which has functions in DNA double-strand break repair and telomere stability. An intron in GECA18s01957g encoding a mitochondrial isocitrate dehydrogenase reaches the unusual size for *G. candidum* of 1428 nt, whereas the rest of the introns have a size between 44 nt and 750 nt, the median being 71 nt. Intron size distribution is overwhelmingly centered between 60 nt and 80 nt (Supplementary Figure 3). Finally, the most striking feature of the spliceosomal introns in *G. candidum* is the poor conservation of the 5' splice site and the branch point. In previously-sequenced yeast genomes, this sequence is relatively well conserved. The consensus is GTATGT in *S. cerevisiae* and the large majority of the Saccharomycotina yeasts, whereas *Y. lipolytica* stands out with a GTGAGT consensus. However, *G. candidum* presents a mix of these two patterns. Outside the first two bases [11], the other bases are less conserved than in other yeasts (Supplementary Figure 4). Indeed, this pattern is closer to that observed in the Pezizomycotina/Basidiomycota than to other Saccharomycotina. Examples of 5' splice site patterns for a Pezizomycotina species, *Fusarium graminearum,* and a Basidiomycota species, *Cryptoccocus neoformans*, are shown in Supplementary Figure 4.

Similarly, the branch point sequence is also less conserved than is the case in the other Saccharomycotina yeasts. The most common sequence in *G. candidum* was found to be NNCTAAC (72% of the total), followed by NNCTAAT (12%), NNTTAAC (7%) and NNCTGAC (5%). By comparison, the consensus sequence NNCTAAC is found in 95% of *Y. lipolytica*, 92% of *D. hansenii* and 93% of *S. cerevisiae* introns. The median S2 distance separating the branch point sequence from the 3' splice site (AG) was 12 nucleotides.

The spliceosome is a large ribonucleoprotein complex that catalyzes intron removal from precursor mRNAs via two trans-esterification steps. The spliceosome comprises five small nuclear RNAs (snRNAs) organized in small nuclear ribonucleoprotein particles (snRNPs) and numerous proteins [12]. *G. candidum* homologous snRNP and non-snRNP protein coding genes are listed in Supplementary Table 7. Among these genes, three (GECA02s09393g, GECA15s01110g and GECA16s02188g) do not have counterparts in other Saccharomycotina and have orthologs only in filamentous fungi. Two other *G. candidum* genes have orthologs only in *Y. lipolytica*

and in filamentous fungi (GECA03s02815g and GECA04s02056g). Interestingly, *G. candidum* has two paralogs coding for Yju2p and for Prp43p with, in each family, one paralog of the filamentous fungal type (GECA15s01110g for Yju2p and GECA16s02188g for Prp43p). In *S. cerevisiae,* Yju2p functions in the first step of trans-esterification and has a possible role in positioning the branch point during the reaction [13]. Prp43p codes for a DExD/H-box RNA helicase involved in spliceosome disassembly together with the two cofactors Ntr1p and Ntr2p [14]. *G. candidum* has only one of these two cofactors; it displays low sequence similarity with the Saccharomycotina counterparts but it is well conserved compared to the filamentous fungal orthologs.

Mating type

The *GcMATA* coding sequence presented similarity with the previously-described HMG-box proteins in yeasts and is located between the *APC5* and *SLA2* orthologs in *G. candidum* (Supplementary Figure 6). To confirm that this gene corresponded to a mating type gene, we ran a search for the other idiomorphs by PCR amplification of different parts of this region in a set of *G. candidum* stains of various origins. First, primers were chosen to amplify a fragment of 279 bp within the *MATA* gene and were subsequently used in PCR on 62 *G. candidum* strains preserved at the CIRM-Levures (http://www6.inra.fr/cirm/Levures). PCR products of the expected size were obtained for 37 strains. No PCR product was observed for 25 strains, suggesting that these strains may not carry the *MATA* gene but the opposite mating type. Hence, primers located in the regions flanking the *MATA* gene were designed and used to amplify the corresponding region in three strains that failed to give a positive signal with the *MATA*-specific primers. A 1.5 kb region was successfully amplified from the three strains including the *G. candidum* type strain CBS 615.84^{NT}, and was entirely sequenced: it contained a CDS of 339 aa, different from the Gc*MATA* gene, in an otherwise identical environment. The product of this gene presented sequence similarity with various MATα proteins in the region of the alpha box (not shown). This gene was therefore called *MATB*. We thus confirmed that *G. candidum* is heterothallic.

Comparison of the structure of the mating type loci in several yeast and fungal species (Supplementary Figure 6) indicated that the *G. candidum* locus contains only two genes—Gc*MATA* and Gc*MATB*—and thus resembles some filamentous fungi such as *Aspergillus* species [15], but not Saccharomycotina yeasts. A roughly 2 kb region separates the *MATA* gene from the gene upstream of *MATA*, but we did not identify a valid CDS in this region. The *SLA2* and the *SUI1* genes are the immediate neighbors to the right of the sexual locus in the examples of Saccharomycotina species shown in Supplementary Figure 6. However, the neighboring genes on the left-end side of the mating type loci showed more variability: an ortholog of *DIC1* is found in *Lachancea kluyveri* and *Ogatea angusta* and an ortholog of *APN2* in *Y. lipolytica*, *Neurospora crassa* and *Trichoderma reesei*. Interestingly, the left flanking region of the *G. candidum* sexual locus is not conserved and has very likely been rearranged. Indeed, the neighboring gene of the *G. candidum* sexual locus is *APC5,* which is found near the sexual locus of some Pezizomycotina species but separated from this locus by two genes, *COX13* and *APN2*. A search for *G. candidum COX13* and *APN2* indicated that they are localized on different scaffolds. Genomic rearrangements located at the border of the sexual locus have previously been seen in yeast species that are

able to switch mating type; this switching was proposed as responsible for an erosion of this locus [16]. Our description of the organization of the *G. candidum* sexual locus indicates that rearrangement at the border of the locus may be more widespread, potentially affecting Dipodascaceae as well as other Saccharomycetes family yeasts in which mating type switching takes place.

Cazymes in the *G. candidum* genome

Among the 133 glycoside hydrolase (GH) families integrated in the CAZy database (http://www.cazy.org; [17]), fungal endoglucanases are so far classified into nine GH families. Endo-β-1,4-glucanases (EC 3.2.1.4) act in synergy with other enzymatic activities (including hydrolytic and oxidative enzymes) and help degrade the main plant cell wall component, i.e. cellulose [18]. The endoglucanases of one of the GH families, GH45, randomly cleave glycosidic bonds on cellulose polymers, releasing cello-oligosaccharides as end-products [19]. One GH45 family, comprising four members (not found in Saccharomycotina except one gene belonging to one of these families in *K. pastoris*) was detected in *G. candidum* (Supplementary data 3).

Surprisingly, four members of family AA9 of lytic polysaccharide monooxygenases (LPMOs) were also identified. LPMOs participate in cellulose targeting but via oxidative mechanisms, contrary to the hydrolytic mechanism of the GH counterparts. The presence of four LPMOs is unexpected in *G. candidum* as AA9 members are only found in the wood-decaying fungi, prevalently in white-rot basidiomycete fungi. Comparison with other yeasts (Supplementary Data “CAZy annotation”) showed that AA9 members were exclusively identified in *G. candidum*. Moreover, *G. candidum* is the only Saccharomycotina yeast to possess genes encoding enzymes containing domains of the carbohydrate-binding module family 1 (CBM1), which specifically binds crystalline cellulose [20]. CBM1 are primarily found in the fungal kingdom [21] and are usually restricted to the genome of wood-rot fungi. Previous works showed that carbohydrate binding modules enable increase of the enzyme concentration in the vicinity of the substrate [22]. Moreover, CBMs may also be involved in the destructuration of polysaccharides on the substrate fibrils [23]. Remarkably, *G. candidum* carries eight CBM1 members, and each CBM1 is linked to all the GH45 endoglucanases. Finally, *G. candidum* has two AA1_2-family ferroxidases like other yeasts but only *G. candidum* has one AA1 multicopper oxidase close to laccases. To our knowledge, *G. candidum* is the only yeast to retain this broad lignocellulolytic repertoire with representatives of typical filamentous fungi-associated families (AA1, AA9, CBM1).

HGT from Basidiomycota

Polyamines are involved in numerous processes and are essential for growth [24]. In *S. cerevisiae*, polyamine synthesis is initiated by two reactions: decarboxylation of L-ornithine by the *SPE1* gene results in putrescine, and decarboxylation of S-adenosyl-L-methionine by the *SPE2* gene results in S-adenosyl-methionamine. Transfer of an aminopropyl group from S-adenosyl-methioninamine to putrescine by spermidine synthase (encoded by the *SPE3* gene) results in spermidine. A second aminopropyl group is then incorporated into

spermidine by spermine synthase (*SPE4*) to yield spermine. While *SPE3* is essential for *S. cerevisiae* growth, *SPE4* is not [25]. Most filamentous fungi do not contain spermine, and these organisms contain a spermidine synthase encoded by an ortholog of *SPE3* as well as a second gene encoding a spermidine synthase, phylogenetically unrelated to either *SPE3* or *SPE4*.

G. candidum possesses a gene, GECA15s02364g, which shows a high degree of conservation with *SPE3* and *SPE4*. Interestingly, *G. candidum* also carries another gene, GECA13s02485g, that is similar to the second spermidine synthase of filamentous fungi, and indeed groups with the Basidiomycota sequences in phylogenetic analysis (Figure 4). This indicates that *G. candidum* has a very unusual complement of spermidine synthases, an *SPE3*-like spermidine synthase and a second spermidine synthase, derived from that of the filamentous fungi. This could imply that the *SPE4* gene, very likely derived from a duplication of the *SPE3* gene [25], was lost in *G. candidum* and that a spermidine synthase has been acquired through HGT from a basidiomycete. Therefore, the polyamine synthase gene complement of *G. candidum* is consistent with an involvement of polyamines in hyphal and pseudo-hyphal growth by a mechanism similar to that acting in filamentous fungi [26].

References of supplementary material

1. Neuveglise, C., Chalvet, F., Wincker, P., Gaillardin, C. & Casaregola, S. Mutator-like element in the yeast *Yarrowia lipolytica* displays multiple alternative splicings. *Eukaryot Cell* **4**, 615-24 (2005).
2. Flutre, T., Duprat, E., Feuillet, C. & Quesneville, H. Considering transposable element diversification in de novo annotation approaches. *PLoS One* **6**, e16526 (2011).
3. Jeffries, T.W. et al. Genome sequence of the lignocellulose-bioconverting and xylose-fermenting yeast *Pichia stipitis*. *Nat Biotechnol* **25**, 319-26 (2007).
4. Lynch, D.B., Logue, M.E., Butler, G. & Wolfe, K.H. Chromosomal G + C content evolution in yeasts: systematic interspecies differences, and GC-poor troughs at centromeres. *Genome Biol Evol* **2**, 572-83 (2010).
5. Borkovich, K.A. et al. Lessons from the genome sequence of *Neurospora crassa*: tracing the path from genomic blueprint to multicellular organism. *Microbiol Mol Biol Rev* **68**, 1-108 (2004).
6. Cambareri, E.B., Aisner, R. & Carbon, J. Structure of the chromosome VII centromere region in Neurospora crassa: degenerate transposons and simple repeats. *Mol Cell Biol* **18**, 5465-77 (1998).
7. Ravin, N.V. et al. Genome sequence and analysis of methylotrophic yeast *Hansenula polymorpha* DL1. *BMC Genomics* **14**, 837 (2013).
8. Gente, S. et al. Intra-species chromosome-length polymorphism in *Geotrichum candidum* revealed by pulsed field gel electrophoresis. *Int J Food Microbiol* **76**, 127-34 (2002).
9. Poulter, R.T., Goodwin, T.J. & Butler, M.I. The nuclear-encoded inteins of fungi. *Fungal Genet Biol* **44**, 153-79 (2007).
10. Gaillardin, C., Neuveglise, C., Kerscher, S. & Nicaud, J.M. Mitochondrial genomes of yeasts of the *Yarrowia* clade. *FEMS Yeast Res* **12**, 317-31 (2012).
11. Goodwin, S.B. et al. Finished genome of the fungal wheat pathogen Mycosphaerella graminicola reveals dispensome structure, chromosome plasticity, and stealth pathogenesis. *PLoS Genet* **7**, e1002070 (2011).
12. Chen, H.C. & Cheng, S.C. Functional roles of protein splicing factors. *Biosci Rep* **32**, 345-59 (2012).
13. Chiang, T.W. & Cheng, S.C. A weak spliceosome-binding domain of Yju2 functions in the first step and bypasses Prp16 in the second step of splicing. *Mol Cell Biol* **33**, 1746-55 (2013).
14. Tsai, R.T. et al. Spliceosome disassembly catalyzed by Prp43 and its associated components Ntr1 and Ntr2. *Genes Dev* **19**, 2991-3003 (2005).
15. Dyer, P.S. & O'Gorman, C.M. Sexual development and cryptic sexuality in fungi: insights from Aspergillus species. *FEMS Microbiol Rev* **36**, 165-92 (2012).
16. Gordon, J.L. et al. Evolutionary erosion of yeast sex chromosomes by mating-type switching accidents. *Proc Natl Acad Sci U S A* **108**, 20024-9 (2011).
17. Lombard, V., Golaconda Ramulu, H., Drula, E., Coutinho, P.M. & Henrissat, B. The carbohydrate-active enzymes database (CAZy) in 2013. *Nucleic Acids Res* **42**, D490-5 (2014).
18. Davies, G.J. et al. Structure and function of endoglucanase V. *Nature* **365**, 362-4 (1993).
19. Karlsson, J., Siika-aho, M., Tenkanen, M. & Tjerneld, F. Enzymatic properties of the low molecular mass endoglucanases Cel12A (EG III) and Cel45A (EG V) of *Trichoderma reesei*. *J Biotechnol* **99**, 63-78 (2002).
20. Boraston, A.B., Bolam, D.N., Gilbert, H.J. & Davies, G.J. Carbohydrate-binding modules: fine-tuning polysaccharide recognition. *Biochem J* **382**, 769-81 (2004).
21. Palomares-Rius, J.E. et al. Distribution and evolution of glycoside hydrolase family 45 cellulases in nematodes and fungi. *BMC Evol Biol* **14**, 69 (2014).
22. Bolam, D.N. et al. Pseudomonas cellulose-binding domains mediate their effects by increasing enzyme substrate proximity. *Biochem J* **331 (Pt 3)**, 775-81 (1998).

23. Wang, L., Zhang, Y. & Gao, P. A novel function for the cellulose binding module of cellobiohydrolase I. *Sci China C Life Sci* **51**, 620-9 (2008).
24. Wallace, I.M., O'Sullivan, O., Higgins, D.G. & Notredame, C. M-Coffee: combining multiple sequence alignment methods with T-Coffee. *Nucleic Acids Res* **34**, 1692-9 (2006).
25. Hamasaki-Katagiri, N., Katagiri, Y., Tabor, C.W. & Tabor, H. Spermine is not essential for growth of *Saccharomyces cerevisiae*: identification of the SPE4 gene (spermine synthase) and characterization of a *spe*4 deletion mutant. *Gene* **210**, 195-201 (1998).
26. Cheng, Y. et al. Polyamines stimulate hyphal branching and infection in the early stage of *Glomus etunicatum* colonization. *World J Microbiol Biotechnol* **28**, 1615-1621 (2012).
