# Supplementary material for: Differential gene retention as an evolutionary mechanism to generate biodiversity and adaptation in yeasts
Source: Sci Rep. 2015 Jun 25;5:11571. doi: 10.1038/srep11571 (PMC4479816; doi:10.1038/srep11571)
Supplement: Supplementary Information [file srep11571-s7.doc]

**Figure 3 legend**

Figure 3. **Phylogenetic position of the *G. candidum* gene GECA13s02485g potentially encoding a spermine synthase among Pezizomycotina and Basidiomycota orthologs.** Sequences of the most closely related fungal genes to GECA13s02485g were retrieved from NCBI after a blast comparison and aligned using Muscle. The alignment was curated using Gblocks and the phylogenetic reconstruction was performed using phyml with programs and default settings implemented in phylogeny.fr (<http://www.phylogeny.fr/>). The orthologous sequences were from the following species: 1, Sordaria macrospora k-hell; 2, Myceliophthora thermophila ATCC 42464; 3, Thielavia terrestris NRRL 8126; 4, Chaetomium thermophilum var. thermophilum DSM 1495; 5, Ophiostoma piceae UAMH 11346; 6, Sporothrix schenckii ATCC 58251; 6, Togninia minima UCRPA7; 7, Colletotrichum graminicola M1.001; 8, Verticillium dahliae VdLs.17; 9, Fusarium oxysporum Fo5176; 10, Nectria haematococca mpVI 77-13-4; 11, Metarhizium acridum CQMa 102; 12, Claviceps purpurea 20.1; 13, Trichoderma reesei QM6a; 14, Cordyceps militaris CM01; 15, Sclerotinia sclerotiorum 1980 UF-70; 16, Blumeria graminis f. sp. hordei DH14; 17, Aspergillus nidulans FGSC A4; 18, Penicillium chrysogenum Wisconsin 54-1255; 19, Byssochlamys spectabilis No. 5; 20, Coccidioides immitis RS; 21, Uncinocarpus reesii 1704; 22, Ajellomyces dermatitidis ATCC 18188; 23, Paracoccidioides brasiliensis Pb03; 24, Talaromyces marneffei ATCC 18224; 25, Exophiala dermatitidis NIH/UT8656; 26, Cladophialophora carrionii CBS 160.54; 27, Bipolaris maydis ATCC 48331; 28, Setosphaeria turcica Et28A; 29, Phaeosphaeria nodorum SN15; 30, Macrophomina phaseolina MS6; 31, Neofusicoccum parvum UCRNP2; 32, Coniosporium apollinis CBS 100218; 33, Dothistroma septosporum NZE10; 34, Sphaerulina musiva SO2202; 34, Zymoseptoria tritici IPO323; 35, Pyronema omphalodes CBS 100304; 36, Tuber melanosporum; 37, Dactylellina haptotyla CBS 200.50; 38, Arthrobotrys oligospora ATCC 24927; A, Wallemia sebi CBS 633.66; B, Wallemia ichthyophaga EXF-994; C, Rhodosporidium toruloides NP11; D, Puccinia graminis f. sp. tritici CRL 75-36-700-3; E, Piriformospora indica DSM 11827; F, Fomitiporia mediterranea MF3/22; G, Punctularia strigosozonata HHB-11173 SS5; H, Serpula lacrymans var. lacrymans S7.9; I, Phanerochaete carnosa HHB-10118-sp; J, Fomitopsis pinicola FP-58527 SS1; K, Fibroporia radiculosa; L, Ceriporiopsis subvermispora B; M, Dichomitus squalens LYAD-421 SS1; N, Trametes versicolor FP-101664 SS1; O, Gloeophyllum trabeum ATCC 11539; P, Moniliophthora roreri MCA 2997; Q, Agaricus bisporus var. bisporus H97; R, Laccaria bicolor S238N-H82; S, Schizophyllum commune H4-8; T, Coprinopsis cinerea okayama7#130; U, Stereum hirsutum FP-91666 SS1;
